# Discovery of potent PROTAC degraders of Pin1 for the treatment of acute myeloid leukemia†

**DOI:** 10.1039/d3sc06558h

**Published:** 2024-02-28

**Authors:** Yunkai Shi, Minmin Liu, Mengna Li, Yiwen Mao, Jingkun Ma, Ruikai Long, Miaomiao Xu, Yaxi Yang, Wenlong Wang, Yubo Zhou, Jia Li, Bing Zhou

**Affiliations:** a School of Pharmaceutical Science and Technology, Hangzhou Institute for Advanced Study, University of Chinese Academy of Sciences Hangzhou 310024 China zhoubing2012@hotmail.com; b State Key Laboratory of Drug Research, Shanghai Institute of Materia Medica, Chinese Academy of Sciences 555 Zu Chong Zhi Road Shanghai 201203 China ybzhou@simm.ac.cn jli@simm.ac.cn zhoubing@simm.ac.cn; c University of Chinese Academy of Sciences 19 Yuquan Road Beijing 100049 China; d School of Pharmaceutical Science, Jiangnan University Wuxi 214122 China wenlongwang@jiangnan.edu.cn; e Zhongshan Institute for Drug Discovery, Shanghai Institute of Materia Medica, Chinese Academy of Sciences Zhongshan Tsuihang New District Guangdong 528400 China; f Shandong Laboratory of Yantai Drug Discovery, Bohai Rim Advanced Research Institute for Drug Discovery Yantai Shandong 264117 China; g School of Chinese Materia Medica, Nanjing University of Chinese Medicine Nanjing 210023 China

## Abstract

Peptidyl-prolyl *cis*/*trans* isomerase NIMA-interacting 1 (Pin1) is overexpressed and/or overactivated in many human cancers and has been shown to play a critical role during oncogenesis. Despite the potential of Pin1 as a drug target, its successful targeting has proved to be challenging. We speculate that only blocking the enzymatic function of Pin1 with inhibitors may not be sufficient to lead to a total loss-of-function. Here, we report the discovery of P1D-34, a first-in-class and potent PROTAC degrader of Pin1, which induced Pin1 degradation with a DC_50_ value of 177 nM and exhibited potent degradation-dependent anti-proliferative activities in a panel of acute myeloid leukemia (AML) cell lines. In contrast, Pin1 inhibitor Sulfopin did not show activity. More significantly, P1D-34 could sensitize Bcl-2 inhibitor ABT-199 in Bcl-2 inhibitor-resistant AML cells, highlighting the potential therapeutic value of targeted Pin1 degradation for Bcl-2 inhibitor-resistant AML treatment. Further mechanism study revealed that P1D-34 led to the up-regulation of ROS pathway and down-regulation of UPR pathway to induce cell DNA damage and apoptosis. Notably, we further demonstrated that treatment with the combination formula of glucose metabolism inhibitor 2-DG and P1D-34 led to a notable synergistic anti-proliferative effect, further expanding its applicability. These data clearly reveal the practicality and importance of PROTAC as a preliminary tool compound suitable for assessment of Pin1-dependent pharmacology and a promising strategy for AML treatment.

## Abstract 2

The first PROTAC degrader of Pin1 was developed and exhibited potent degradation-dependent anti-proliferative activities in acute myeloid leukemia cells.

## Graphical abstract

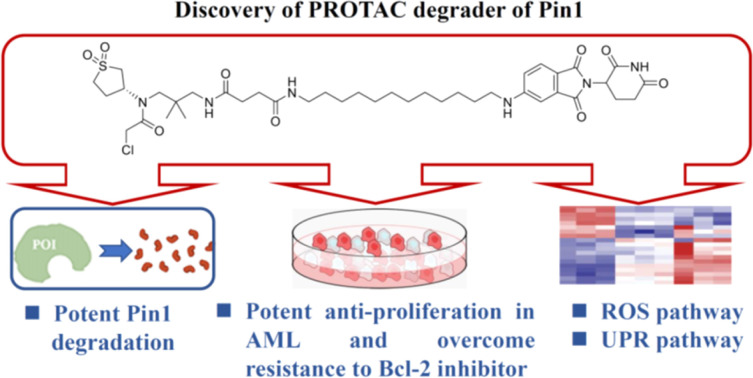

## Introduction

Peptidyl-Prolyl Isomerases (PPIases) catalyze the *cis*–*trans* isomerization of peptide bonds N-terminal to proline and function on protein folding and regulation. Peptidyl-prolyl *cis*–*trans* isomerase NIMA-interacting 1 (Pin1) that is the only known phosphorylation-dependent isomerase among the ∼30 PPIases, specifically recognizes and isomerizes phosphorylated Ser/Thr–Pro amide bonds.^1^

Pin1 is involved in the regulation of kinase signaling processes by altering the ratio of *cis*–/*trans*-conformers of phosphorylated proteins and has been shown to play a critical role during oncogenesis.^2–7^ Moreover, Pin1 is overexpressed and/or overactivated in many human cancers and patients who carry overexpression of Pin1 are associated with poor clinical prognosis.^8,9^ Notably, Pin1 has been shown to simultaneously activate more than 50 oncogenes to promote several cancer-driving signaling pathways, including cyclin D1, c-Myc, β-catenin, NF-κB, ERα, and mutant p53, while it simultaneously inactivates more than 20 tumor suppressors and growth inhibitors.^4,5,10–13^ Collectively, Pin1 is potentially an attractive therapeutic target for human cancers.

Unfortunately, successful development of small-molecule Pin1 inhibitors has proved to be challenging, due to its small and shallow enzymatic pocket, as well as the need of a molecule bearing a negatively charged moiety to interface with its catalytic center.^14–16^ Moreover, Pin1 inhibitors that have been reported to date generally lack desirable potency, selectivity, cell permeability, stability or a clear mechanism of action at the cellular level.^17–23^ We speculate that only blocking the enzymatic function of Pin1 with inhibitors may not be sufficient to lead to a total loss-of-function. Therefore, there is a clear need to develop a new therapeutic approach to targeting Pin1.

Proteolysis Targeting Chimeras (PROTACs) strategy has gained tremendous momentum as a promising approach in the discovery and development of a new type of therapeutics for disease treatment.^24–36^ PROTACs is an advanced technology engineered to degrade pathogenic proteins through the ubiquitin-proteasome system and is particularly attractive for its ability to target traditionally undruggable or difficult-to-drug targets. Moreover, despite its infancy, the recently emerging covalent PROTACs further offer several unique advantages, such as targeting proteins without noncovalent binders, and combination of the benefits of both covalent inhibitors and PROTACs.^37–39^ Generally, covalent PROTACs can covalently bind to the E3 ligases or protein of interest, while the latter is unable to be catalytic. More significantly, unlike classic inhibitors, PROTAC has the advantage of simultaneously regulating the enzymatic and non-enzymatic protein functions, thus providing a potential strategy to compensate for the shortcoming of inhibitors and explore the new therapeutics.^40^

In this context, with our continuing interest in design of PROTACs in exploring non-enzymatic function of target proteins,^41^ we describe the discovery of a first-in-class covalent Pin1 PROTAC molecule P1D-34 that coupled the covalent Pin1 inhibitor Sulfopin to a cereblon ligand and induced potent Pin1 degradation with a DC_50_ value of 177 nM. Significantly, this PROTAC degrader exhibited potent cell growth inhibition against a panel of acute myeloid leukemia (AML) cell lines which was degradation-dependent, with Pin1 inhibitor Sulfopin showing no activity. More significantly, P1D-34 in combination with Bcl-2 inhibitor ABT-199 displayed notable synergistic anti-proliferative activities in ABT-199-resistant AML cell lines, further highlighting the potential therapeutic value of targeted Pin1 degradation. Collectively, this work highlights the ability of Pin1 PROTAC degraders to regulate nonenzymatic and AML-relevant functions of Pin1, and describes Pin1 PROTAC as a chemical tool for the further study of Pin1 biology.

## Results and discussion

### Design and characterization of Pin1 degraders

**Fig. 1:**
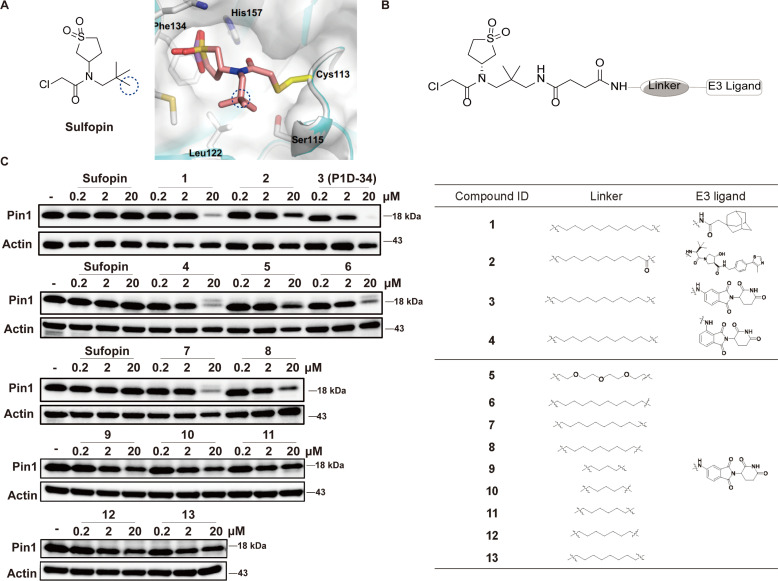
Design of Pin1 degraders and immunoblot analysis of Pin1. (A) Chemical structure of Sulfopin and cocrystal structure (PDB: 6VAJ) of Pin1 in complex with Sulfopin. (B) Structures of Pin1 degraders. (C) Immunoblot analysis for Pin1 in MV-4-11 cells treated with the indicated compounds for 24 h, *n* = 3 independent experiments.

To develop Pin1 PROTACs, Sulfopin, a potent, selective and covalent Pin1 inhibitor, was chosen as a Pin1-targeting ligand.^18^ Cocrystal structure of Pin1 and Sulfopin (Fig. 1A) reveals that the methyl group of the Sulfopin is exposed to solvent and is a suitable tethering site for the design of potential PROTACs. Importantly, the group of London have already established an exit vector strategy and chemistry for linker attachment when they developed their affinity probe.^18^ By connecting London's intermediate with hydrophobic tagging (1) and different E3 ligase ligands such as Von Hippel-Lindau (VHL) ligand (2) and cereblon (CRBN) ligand (3), compounds 1–3 were first synthesized and tested (Fig. 1B). Compounds 1–3 induced an effective degradation of Pin1 at 20 μM in MV-4-11 cells (Fig. 1C and ESI Table S1†) and CRBN-based PROTAC 3 was more effective than degrader 1 with hydrophobic tagging and VHL-based PROTAC 2. Changing the amino group of 3 from *meta* position to *ortho* position gave 4 that was less potent than 3. Next, several CRBN-based degraders 5–13 with different linker length were synthesized. Compounds 5–13 with shorter linkers (4–11 methylene group) were able to induce effective degradation of Pin1 but were less potent than 3 (Fig. 1C and ESI Table S1†). On the basis of the analysis of the western blots, compound 3 (P1D-34) stood out as the most potent degrader and was selected for further evaluation.

### Identification of P1D-34 as a Pin1 PROTAC degrader

**Fig. 2:**
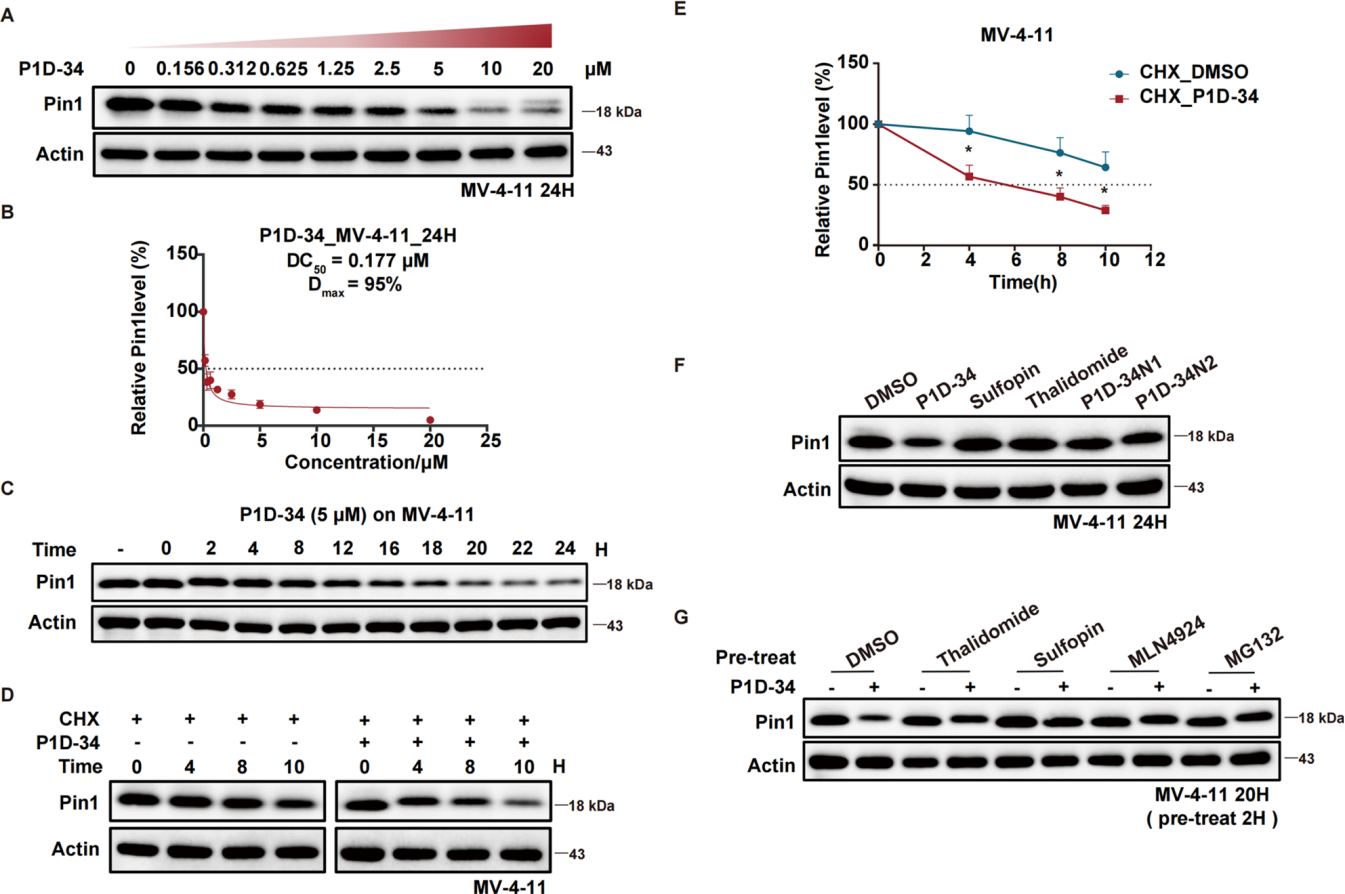
Degradation profiling of Pin1. (A) Immunoblots for Pin1 in MV-4-11 cells after treatment with the indicated concentrations of P1D-34 for 24 h, *n* = 3 independent experiments. (B) Determination of DC_50_ value of P1D-34 in MV-4-11 cells. Relative Pin1 protein level was quantified and normalized over corresponding Actin with Image Lab. *n* = 3 independent experiments. (C) Immunoblots for Pin1 in MV-4-11 cells treated with 5 μM of P1D-34 at indicated time points. The gray values of Pin1 protein bands were calculated with Image Lab. (D) MV-4-11 cells in the presence of cycloheximide (50 μg mL^−1^) were treated with or without P1D-34 (5 μM) and then Pin1 protein levels were detected at the indicated time points. (E) Quantification of relative Pin1 protein level in (D). *n* = 3 independent experiments. Data are means ± SEM. Significance was analyzed by two-tailed *t* test. *p < 0.05, **p < 0.01, ***p < 0.001, ****p < 0.0001. (F) Immunoblot analysis of Pin1 in MV-4-11 cells treated with DMSO, P1D-34 (5 μM), Suofopin (5 μM), Thalidomide (5 μM), P1D-34N1 (5 μM) and P1D-34N2 (5 μM), *n* = 3 independent experiments. (G) Immunoblot analysis of Pin1 in MV-4-11 cells pre-treated with DMSO, Suofopin (10 μM), Thalidomide (10 μM), MLN-4924 (250 nM), or MG132 (5 μM) for 2 h, and then treated with 5 μM of P1D-34 for 20 h.

The efficacy of P1D-34 induced Pin1 degradation in MV-4-11 cells was next evaluated. As shown in Fig. 2A, P1D-34 effectively induced Pin1 degradation in a dose-dependent manner with a DC_50_ (half-maximal degradation) value of 177 nM and a *D*_max_ of 95% (Fig. 2B). The start of a hook effect was observed at 40 μM (ESI Fig. S2B†). A time course experiment was carried out, exhibiting that more than one-half of Pin1 decrease was observed after 16 h of exposure and 79% of Pin1 degradation was achieved at 24 h (Fig. 2C).

To explore the mechanism of Pin1 degradation, several experiments were performed. The Pin1 degradation was accelerated in the presence of protein synthesis inhibitor cycloheximide and a significant Pin1 decrease was observed at 10 h (Fig. 2D and E). Neither Pin1 inhibitor Sulfopin nor CRBN binder thalidomide was able to induce Pin1 degradation (Fig. 2F). Meanwhile, negative controls P1D-34N1 bearing a methyl group in glutarimide NH to reduce its binding affinity to CRBN and P1D-34N2 that is non-covalent to reduce its binding affinity to Pin1, had a very weak effect on the levels of Pin1 protein (Fig. 2F and ESI Fig. S3† for chemical structure). Furthermore, the addition of Sulfopin or thalidomide rescued Pin1 degradation (Fig. 2G). Taken together, these results demonstrated that the binding of P1D-34 to both CRBN and Pin1 is crucial to the degradation. In addition, addition of NEDD8-activating enzyme inhibitor MLN4924 or proteasome inhibitor MG132, effectively prevented the degradation of Pin1 by P1D-34, indicating a proteasomal and neddylation dependent PROTAC-induced substrate degradation (Fig. 2G).

### P1D-34 induces cell cycle arrest and apoptosis in AML cells

**Fig. 3:**
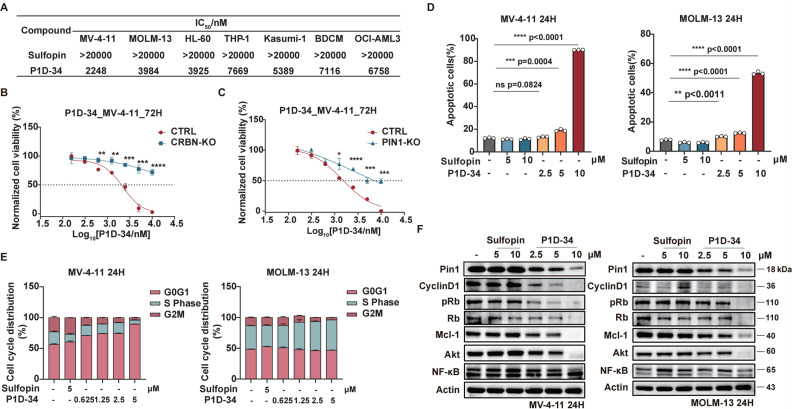
Pin1 degrader induces cell cycle arrest and apoptosis in AML cell lines. (A) Cell growth inhibitory activities of Sulfopin and P1D-34 in AML cells. (B) Growth curves of MV-4-11 CRBN-KO and CTRL cells treated with the indicated concentrations of P1D-34 for 72 h. *n* = 3, significance was analyzed by two-tailed *t* test. *p < 0.05, **p < 0.01, ***p < 0.001, ****p < 0.0001. (C) Growth curves of MV-4-11 PIN1-KO and CTRL cells treated with the indicated concentrations of P1D-34 for 72 h. *n* = 3, significance was analyzed by two-tailed *t* test. *p < 0.05, **p < 0.01, ***p < 0.001, ****p < 0.0001. (D) Cell apoptotic effect on MV-4-11 and MOLM-13 cells induced by the indicated concentrations of Sulfopin and P1D-34. *n* = 3, significance was analyzed by two-tailed *t* test. *p < 0.05, **p < 0.01, ***p < 0.001, ****p < 0.0001. (E) Cell cycle arrest effect on MV-4-11 and MOLM-13 cells induced by the indicated concentrations of Sulfopin and P1D-34. (F) Immunoblots of Pin1 downstream proteins in MV-4-11 cells (left) or MOLM-13 cells (right) treated with the indicated concentrations of Sulfopin and P1D-34.

To probe the biological effects of Pin1 degradation *versus* Pin1 inhibition, we first evaluated the anti-proliferative effect of degrader P1D-34 and inhibitor Sulfopin in a panel of AML cell lines: MV-4-11, MOLM-13, HL-60, THP-1, Kasumi-1, BDCM and OCI-AML3 (Fig. 3A). Although the inhibitor Sulfopin have no effect on cell viability in these seven AML cell lines, P1D-34 effectively inhibits cell growth in all these AML cell lines. To further evaluate whether this cell growth inhibition is extensible to other cancer types, we performed additional experiments in MDA-MB-468 cells that was previously reported to be sensitive to Sulfopin.^18^ The results showed that P1D-34 significantly inhibits the cell growth of MDA-MB-468 cells and is more potent than sulfopin (ESI Fig. S4B, S4C and S4E†). More significantly, P1D-34 exhibited minimal toxicity towards healthy HEK293T cell lines (ESI Fig. S4B and S4C†). Knockout of CRBN led to a significantly reduced P1D-34-mediated degradation of Pin1 (ESI Fig. S5A and S5B†). Consistent with the decreased Pin1 degradation, MV-4-11 CRBN-KO cells were completely insensitive to P1D-34 (Fig. 3B). To further verify that the anti-proliferative activities of P1D-34 is Pin1 degradation dependent and does not come from off-target effect, we obtained MV-4-11 Pin1 knockout cells (ESI Fig. S5C and S5D†) and found that P1D-34 had largely lost its anti-proliferative activities in MV-4-11 Pin1-KO cells (Fig. 3C). Both results indicated that the anti-proliferative activities of P1D-34 is to a large extent Pin1 degradation dependent.

Next, we examined the effects of P1D-34 on apoptosis and cell circle. Compared with inhibitor Supfopin, the degrader P1D-34 significantly increased the percentage of apoptotic cells in MV-4-11 and MOLM-13 cells (Fig. 3D and ESI Fig. S6A†) and obviously induced cell cycle G1/S arrest in a concentration-dependent manner in MV-4-11 cells (Fig. 3E and ESI Fig. S6B†). Pin1 influences the turnover and activity of various proteins, and the effect of P1D-34 on these Pin1 targets was subsequently examined in AML cells. Treatment with P1D-34 led to the downregulation of Pin1 client proteins such as Cyclin D1, pRb, Mcl-1, Akt and c-Myc in a dose-dependent manner in MV-4-11 and MOLM-13 cells, but the level of NF-κB was almost not affected (Fig. 3F and ESI Fig. S7†). In contrast, the inhibitor Sulfopin had minimal effect on the level of these proteins.

### P1D-34 sensitizes ABT-199 against ABT-199-resistant AML cell lines

**Fig. 4:**
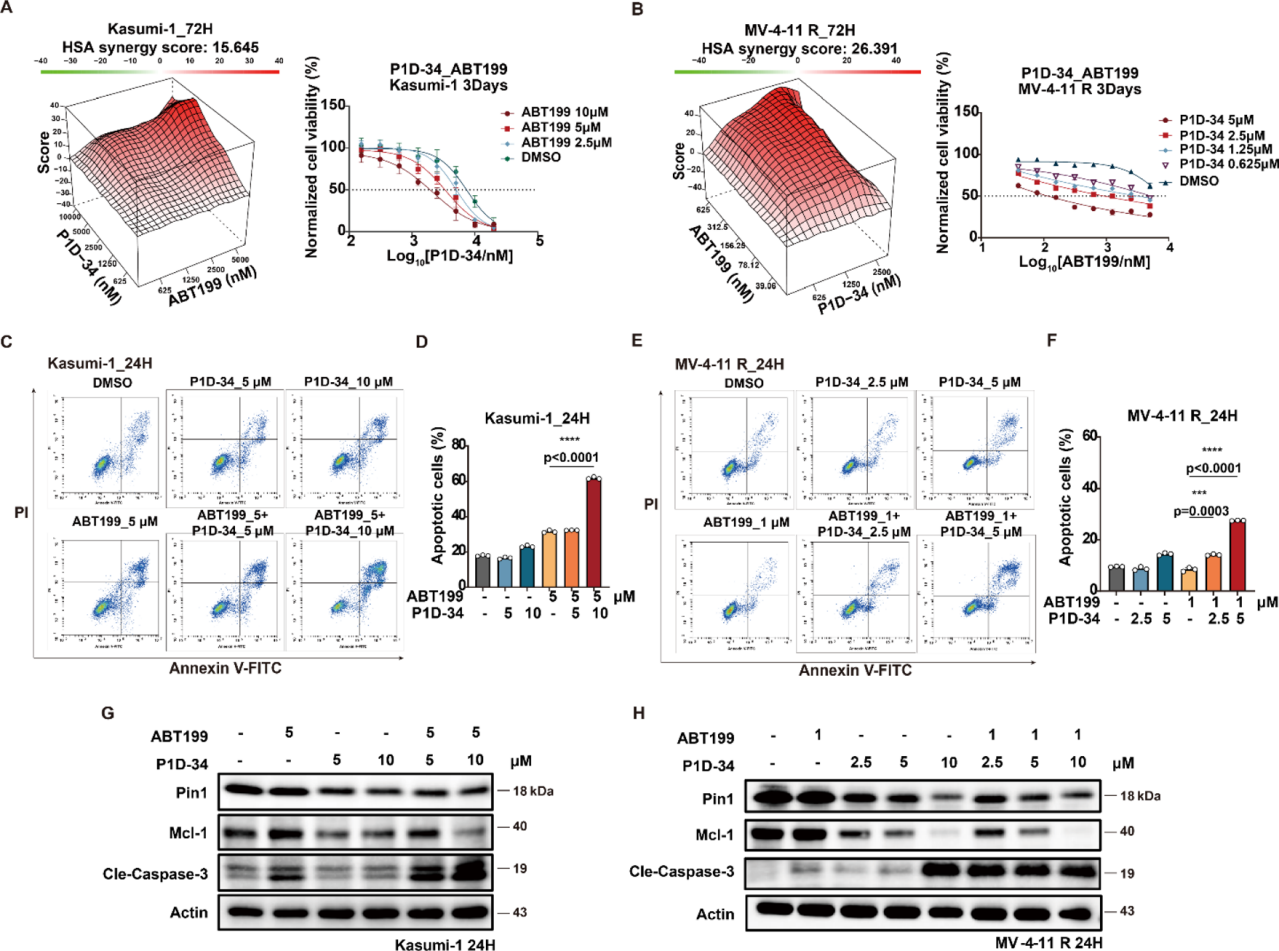
P1D-34 sensitizes ABT-199 in resistant AML cell lines. (A) Excess over HSA synergy plots (left) and growth curves (right) for serial dilutions of P1D-34 in combination with ABT-199 in Kasumi-1 cells. (B) Excess over HSA synergy plots (left) and growth curves (right) for serial dilutions of P1D-34 in combination with ABT-199 in MV-4-11 R cell lines. (C) Apoptotic effect on Kasumi-1 cells induced by co-treatment with P1D-34 and ABT-199. (D) Quantification of apoptosis in (C). *n* = 3, significance was analyzed by two-tailed *t* test. *p < 0.05, **p < 0.01, ***p < 0.001, ****p < 0.0001. (E) Apoptotic effect on MV-4-11 resistant cells induced by co-treatment with P1D-34 and ABT-199. (F) Quantification of apoptosis in (E). *n* = 3, significance was analyzed by two-tailed *t* test. *p < 0.05, **p < 0.01, ***p < 0.001, ****p < 0.0001. Immunoblots for Pin1, Mcl-1 and Cleaved-Caspase3 in Kasumi-1 (G) or MV-4-11 resistant cells (H) after treatment with P1D-34 and/or ABT-199.

The selective Bcl-2 inhibitor ABT-199, also known as venetoclax, is a first-in-class FDA-approved drug, and Mcl-1 is reported to be a significant ABT-199 resistance factor.^42^ Given the observed downregulation of Mcl-1 induced by P1D-34, we wondered whether P1D-34 was able to sensitize ABT-199 in Bcl-2 inhibitor-resistant AML cell lines. To explore the potential drug combination of P1D-34 with ABT-199, primary ABT-199 resistant Kasumi-1 cell line and acquired ABT-199-resistant cell line (MV-4-11 R) (ESI Fig. S8A†) were treated with different concentrations of P1D-34 with ABT-199. The Highest Single Agent (HSA) model was used to determine if the combinated effects on cell growth are synergistic.^43^ As expected, a strong synergistic effect was observed with positive synergy scores in both two ABT-199-resistant cell lines (Fig. 4A and B). Accordingly, the combination of P1D-34 and ABT-199 markedly promoted cell apoptosis in both two ABT-199-resistant cell lines (Fig. 4C–F). In contrast, no synergistic effect was observed in the Sulfopin and ABT-199 combination group in Kasumi-1 cell line (ESI Fig. S8B†).

To understand the mechanism of action underlying the synergistic effect, the level of Mcl-1 and cleaved caspase3 in Kasumi-1 and MV-4-11 R cell lines was examined through immunoblotting (Fig. 4G and H). Treatment with ABT-199 in both two resistant AML cell lines resulted in upregulation of Mcl-1 to a certain extent, while the addition of P1D-34 prominently down-regulated the expression of Mcl-1 and induced significant apoptosis. Taken together, our data demonstrated that P1D-34 was able to sensitize ABT-199-resistant AML cell lines to ABT-199 by down-regulating Mcl-1 and inducing apoptosis.

### P1D-34 up-regulates reactive oxygen species pathway and down-regulates unfolded protein response

**Fig. 5:**
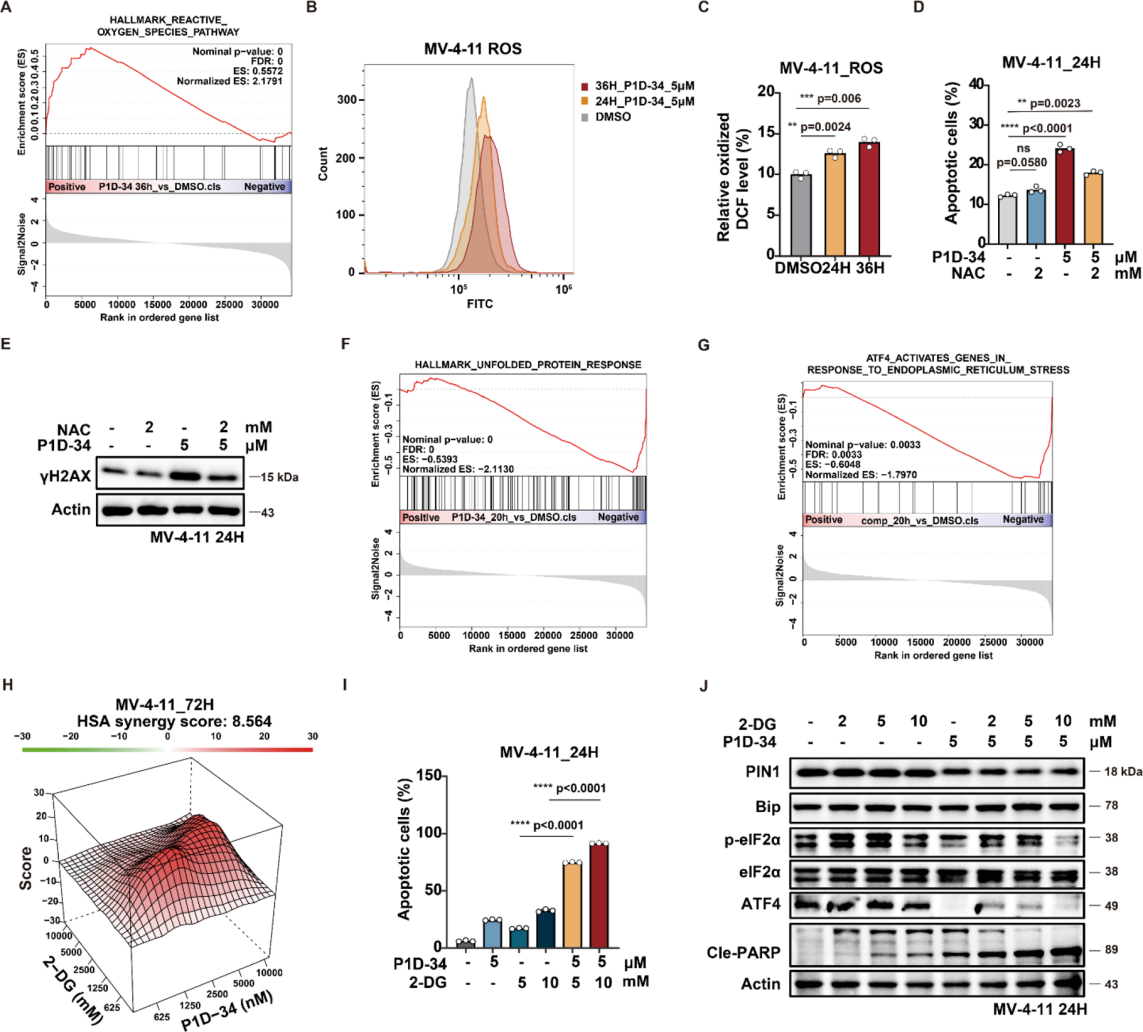
P1D-34 induces DNA damage and apoptosis by releasing ROS generation, and sensitizes 2-DG by down-regulating UPR pathways. (A) GSEA analysis of ROS pathway. (B) Analysis of ROS production by flow cytometry in MV-4-11 cells treated with P1D-34 (5 μM) for 24 and 36 h. (C) Quantification of ROS production in (B). *n* = 3, significance was analyzed by two-tailed *t* test. *p < 0.05, **p < 0.01, ***p < 0.001, ****p < 0.0001. (D) Quantification of cell apoptosis. *n* = 3, significance was analyzed by two-tailed *t* test. *p < 0.05, **p < 0.01, ***p < 0.001, ****p < 0.0001. (E) Immunoblots for γH2AX in MV-4-11 cells treated with P1D-34 and NAC for 24 h. (F) GSEA analysis of UPR pathways. (G) GSEA analysis of AFT4 pathways. (H) Excess over HSA synergy plots for serial dilutions of P1D-34 in combination with 2-DG in MV-4-11 cells. (I) Apoptotic effect on MV-4-11 cells induced by co-treatment with P1D-34 and 2-DG. *n* = 3, significance was analyzed by two-tailed *t* test. *p < 0.05, **p < 0.01, ***p < 0.001, ****p < 0.0001. (J) Immunoblots for ATF4 pathway in MV-4-11 cells after co-treatment of P1D-34 and 2-DG.

To gain further insights into how Pin1 degradation results in antiproliferative activities, transcriptome sequencing was performed (ESI Fig. S9A†). The results showed an up-regulation of reactive oxygen species (ROS) pathway and down-regulation of unfolded protein response (UPR) pathway in MV-4-11 cells after the treatment of P1D-34. Gene set enrichment analysis (GSEA) further revealed the significant up-regulation of ROS pathway in P1D-34 treated group (Fig. 5A).

Moreover, reverse transcription followed by quantitative polymerase chain reaction (RT-qPCR) experiments verified that P1D-34 up-regulated the expression of ROS production related genes in MV-4-11 cells (ESI Fig. S9B†). Importantly, ROS production was also examined and the results demonstrated that P1D-34 significantly increased the ROS production (Fig. 5B and C). In contrast, ROS production was not affected at 24 h and was decreased at 36 h by Sulfopin (ESI Fig. S9C and S9D†).

Addition of ROS scavenger *N*-Acetyl-Cysteine (NAC) decreased apoptosis induced by P1D-34, indicating that P1D-34 induced a ROS-dependent apoptosis (Fig. 5D). ROS can induce DNA damage and affect the DNA damage response (DDR). GSEA revealed a significant enrichment of the DNA damage response pathway upon treatment with P1D-34 (ESI Fig. S9E†). We subsequently examined the phosphorylation of H2AX (γH2AX), a DNA damage marker. As expected, treatment with P1D-34 led to an increased phosphorylation of H2AX and addition of NAC decreased the phosphorylation of H2AX (Fig. 5E). However, Sulfopin had no influence on the phosphorylation of H2AX (ESI Fig. S9F†). Taken together, P1D-34 was able to induce a significant increase of DNA damage and apoptosis by releasing ROS generation.

Unfolded protein response (UPR) is a complex network of signaling pathways which can be activated by endoplasmic reticulum (ER) stress to promote cell survival under stress. GSEA analysis revealed that P1D-34 led to down-regulation of UPR pathways (Fig. 5F). To explore which UPR pathway was affected, we performed GSEA analysis on three gene sets: ATF4, XBP1, and ATF6 (Fig. 5G and ESI Fig. S9G and S9H†). The results demonstrated that ATF4 pathway was mostly affected (Fig. 5G). RT-qPCR further confirmed that P1D-34 significantly down-regulated the expression of ATF4 related genes (ESI Fig. S9I†).

Inhibition of glucose metabolism has recently become an attractive strategy for cancer treatment. 2-Deoxyglucose (2-DG) is an inhibitor of glucose metabolism and currently under clinical evaluation for the treatment of cancer. Meanwhile, 2-DG was reported to induce intracellular ER stress and up-regulate self-protected UPR. We hypothesized that the combination of P1D-34 and 2-DG may have synergistic effects by down-regulating UPR pathways and inducing cancer cell death under stress. As expected, there were notable synergistic antiproliferative effects in MV-4-11 cells when 2-DG was combined with P1D-34 (Fig. 5H). Accordingly, the combination of P1D-34 and 2-DG significantly increased cell apoptosis in MV-4-11 cells (Fig. 5I). To understand the mechanism of action underlying the synergistic effect, the level of p-eIF2α, ATF4, Bip and cleaved PARP was examined through immunoblotting. The combination had no effect on the level of Bip that is a master regulator of UPR. Treatment of 2-DG led to up-regulation of UPR pathway related protein such as p-eIF2α and ATF4, but addition of P1D-34 was able to markedly down-regulate the expression of p-eIF2α and ATF4, and significantly induced apoptosis (Fig. 5J). Taken together, P1D-34 was able to induce a significant increase of DNA damage and apoptosis by releasing ROS generation, and the combination of P1D-34 with 2-DG exhibited obvious synergistic antiproliferative effects by down-regulating UPR pathways.

## Conclusions

Pin1 is a Peptidyl-Prolyl isomerase and its successful targeting has proved to be challenging. Here we reported the development of P1D-34 as the first, potent and covalent Pin1 PROTAC degrader. Pin1 degradation induced by P1D-34 is dose- and time-dependent with a DC_50_ value of 177 nM. We employed AML cell lines to evaluate the therapeutic potential of our Pin1 degrader. Notably, P1D-34 potently inhibits cell growth in seven AML cell lines. In contrast, the Pin1 inhibitor Sulfopin had no effect on cell viability. Further mechanism study revealed that P1D-34 was able to down-regulated Pin1 client proteins such as Cyclin D1, Rb, Mcl-1, Akt, and c-Myc in a dose-dependent manner, with conventional inhibitor Sulfopin showing no effect.

Significantly, our study clearly demonstrated that a strong synergistic effect was observed in two ABT-199-resistant cell lines when ABT-199 was combined with P1D-34, highlighting the potential therapeutic value of targeted Pin1 degradation for the treatment of Bcl-2 inhibitor-resistant AML.

Additionally, further mechanism study revealed that Pin1 PROTAC P1D-34 led to the up-regulation of ROS pathway and down-regulation of UPR pathway to induce cell DNA damage and apoptosis. Considering that glucose metabolism inhibitor 2-DG was reported to induce intracellular ER stress and up-regulate UPR, we examined the combination effect of 2-DG and P1D-34. Significantly, treatment with the combination formula of 2-DG and P1D-34 led to a notable synergistic antiproliferative effect, further expanding its applicability. As Pin1 degraders may have unparalleled benefits over conventional inhibitors in therapeutic treatment, our data suggest that P1D-34 can be a promising leading compounds and chemical probe for Pin1-related disease research, in addition to its potential use for the treatment of AML.

## Data availability

ESI figures, tables, and experimental details as well as ^1^H NMR and ^13^C NMR of compounds are provided in the ESI.†

## Author contributions

Y. S., M. L. and M. L. contributed equally to this work. Y. Z., J. L., and B. Z. designed the study. Y. S., M. L., M. L., Y. M., J. M., R. L., M. X., and Y. Y. carried out *in vitro* experiments and collected the data. Y. S, M. L., Y. Z., and B. Z. wrote the manuscript. W. W. and B. Z. revised the manuscript. All authors have read and approved the article.

## Conflicts of interest

The authors declare that there are no conflicts of interest.

## Supplementary Material

SC-015-D3SC06558H-s001
