# Supplementary material for: Discovery of potent PROTAC degraders of Pin1 for the treatment of acute myeloid leukemia
Source: Chem Sci. 2024 Feb 28;15(13):5027–35. doi: 10.1039/d3sc06558h (PMC10967256; doi:10.1039/d3sc06558h)
Supplement: SC-015-D3SC06558H-s001 [file SC-015-D3SC06558H-s001.pdf]

Electronic Supplementary Material (ESI) for Chemical Science.
This journal is © The Royal Society of Chemistry 2024

Discovery of potent PROTAC degraders of Pin1 for the treatment of acute myeloid leukemia

Yunkai Shi[a,c,h], Minmin Liu[b,d,h], Mengna Li[g,h], Yiwen Mao[a,c], Jingkun Ma[b,c,e], Ruikai Long[a,c], Miaomiao Xu,[b,c] Yaxi Yang[a,b,c,f], Wenlong Wang[d]*, Yubo Zhou[a,b,c,e]*, Jia Li[a,b,c,e,f,g,]*, Bing Zhou[a,b,c,f,g,]*

[a] *School of Pharmaceutical Science and Technology, Hangzhou Institute for Advanced Study, University of Chinese Academy of Sciences, Hangzhou, 310024, China.*

[b] *State Key Laboratory of Drug Research, Shanghai Institute of Materia Medica, Chinese Academy of Sciences, 555 Zu Chong Zhi Road, Shanghai, 201203, China.*

[c] *University of Chinese Academy of Sciences, 19 Yuquan Road, Beijing, 100049, China*

[d] *School of Pharmaceutical Science, Jiangnan University, Wuxi, 214122, China.*

[e] *Zhongshan Institute for Drug Discovery, Shanghai Institute of Materia Medica, Chinese Academy of Sciences, Zhongshan Tsuihang New District, Guangdong, 528400, China.*

[f] *Shandong Laboratory of Yantai Drug Discovery, Bohai Rim Advanced Research Institute for Drug Discovery, Yantai, Shandong, 264117, China*

[g] *School of Chinese Materia Medica, Nanjing University of Chinese Medicine, Nanjing 210023, China.*

[h] *These authors contributed equally to this work.*

* corresponding author: wenlongwang@jiangnan.edu.cn; ybzhou@simm.ac.cn; jli@simm.ac.cn; zhoubing@simm.ac.cn; zhoubing2012@hotmail.com

Contents

1. Supplementary Figures 1
2. Supplementary Tables 8
3. Experimental Section - chemistry 10
    3.1. Chemical Synthesis of compound 1 – 13, P1D-34N1 ,P1D-34N2 and Sulfopin. 13
    3.2. ^{1}H and ^{13}C of compound 1 – 13, P1D-34N1 ,P1D-34N2 and Sulfopin. 23
4. Experimental Section – Biological Assays 39
    4.1. Cell lines and cell culture 39
    4.2. Cell viability assay 39
    4.3. Immunobloting 39
    4.4. Apoptosis assay 40
    4.5. Cell cycle assay 40
    4.6. RT-qPCR 40
    4.7. Reactive oxygen species valuation 40
    4.8. RNA-seq and data analysis 40
    4.9. Statistical analysis 41
5. Raw data 42
References 48

1. Supplementary Figures

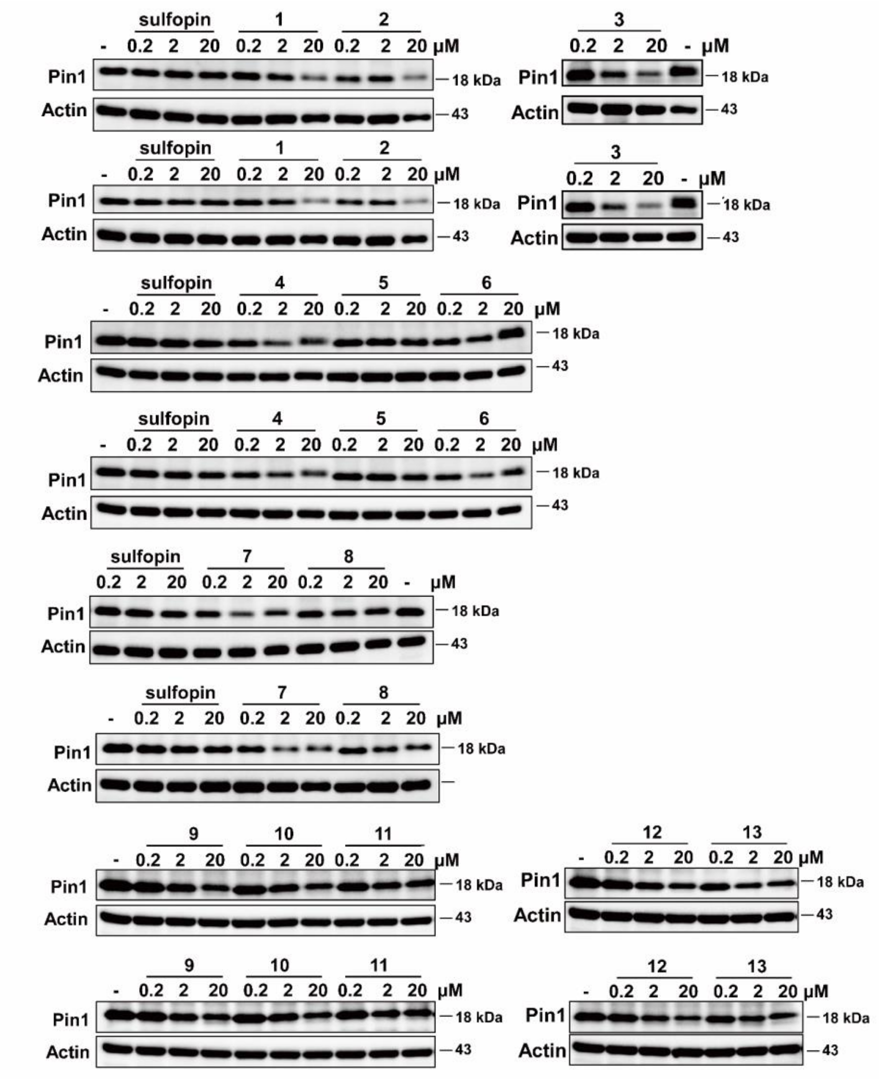

Figure S1. (A) Replicated immunoblot analysis for Pin1 in MV-4-11 cells treated with the indicated compounds for 24 h.

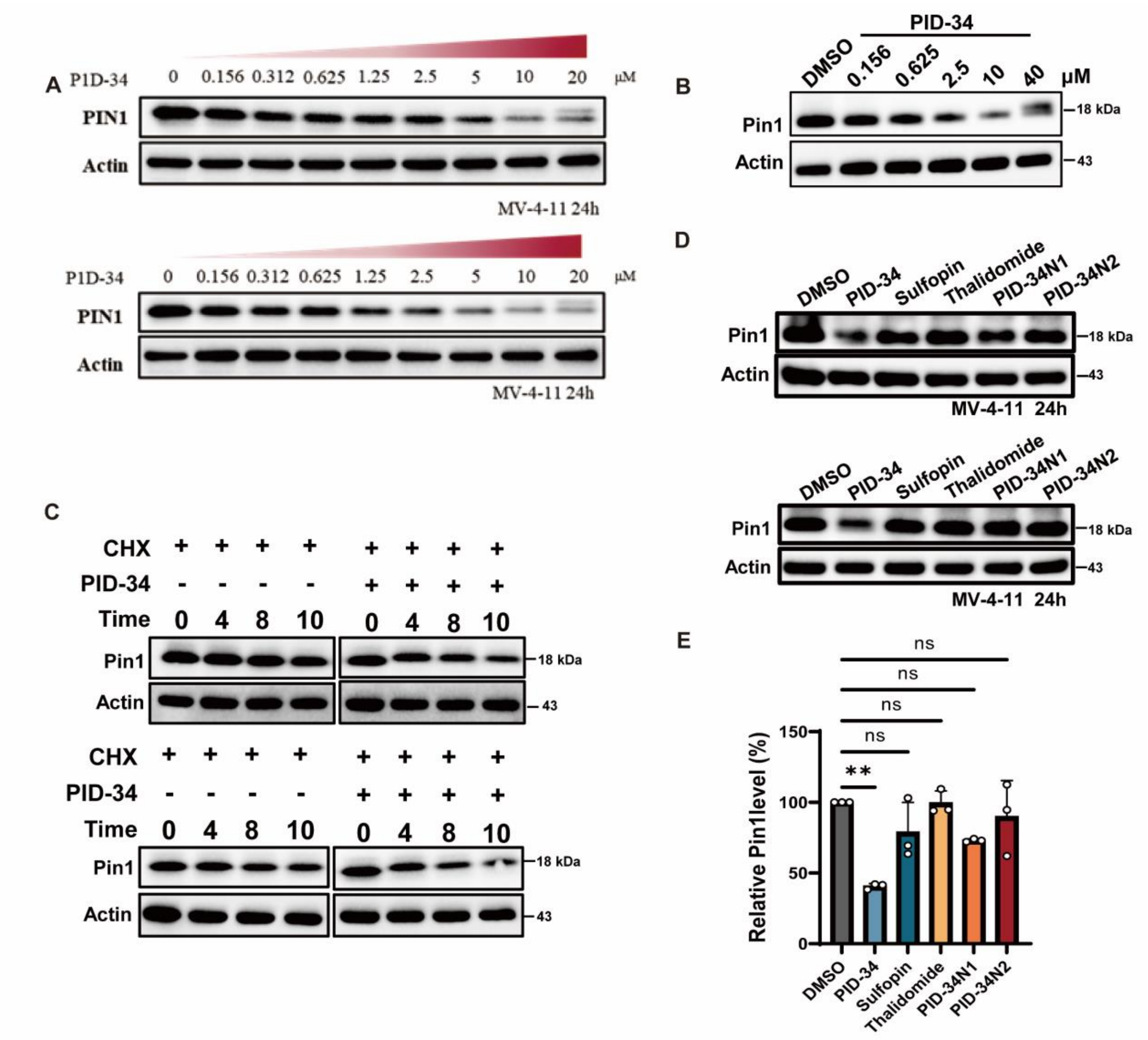

Figure S2. (A) Replicated immunoblot analysis for Pin1 in MV-4-11 cells after treatment with the indicated concentrations of P1D-34 for 24 h. (B) Immunoblot analysis for Pin1 in MV-4-11 cells after treatment with the indicated concentrations of P1D-34 for 24 h. (C) Replicated immunoblot analysis of Pin1 in MV-4-11 cells in the presence of cycloheximide (50 μg/mL) treated with or without P1D-34 (5 μM) and then Pin1 protein levels were detected at the indicated time points. (D) Replicated immunoblot analysis of Pin1 in MV-4-11 cells treated with DMSO, P1D-34 (5 μM), Suofopin (5 μM), Thalidomide (5 μM), P1D-34N1 (5 μM) and P1D-34N2 (5 μM). (E) Quantification of relative Pin1 protein level in (D). n = 3 independent experiments. Data are Means ± SEM. Significance was analyzed by two-tailed t test. *$p < 0.05$, **$p < 0.01$, ***$p < 0.001$, ****$p < 0.0001$.

P1D-34

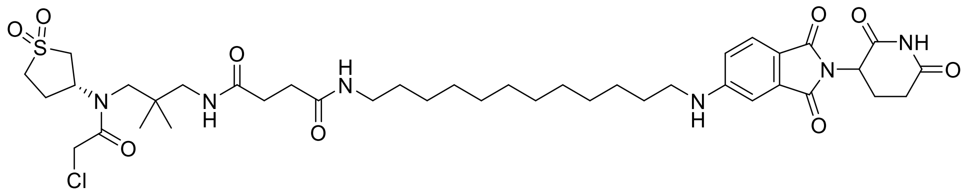

P1D-34N1

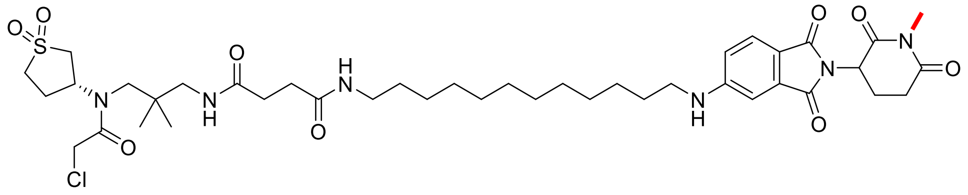

P1D-34N2

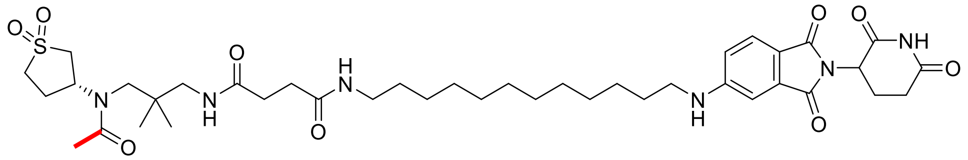

Figure S3. Chemical structures of **P1D-34**, **P1D-34N1** and **P1D-34N2**.

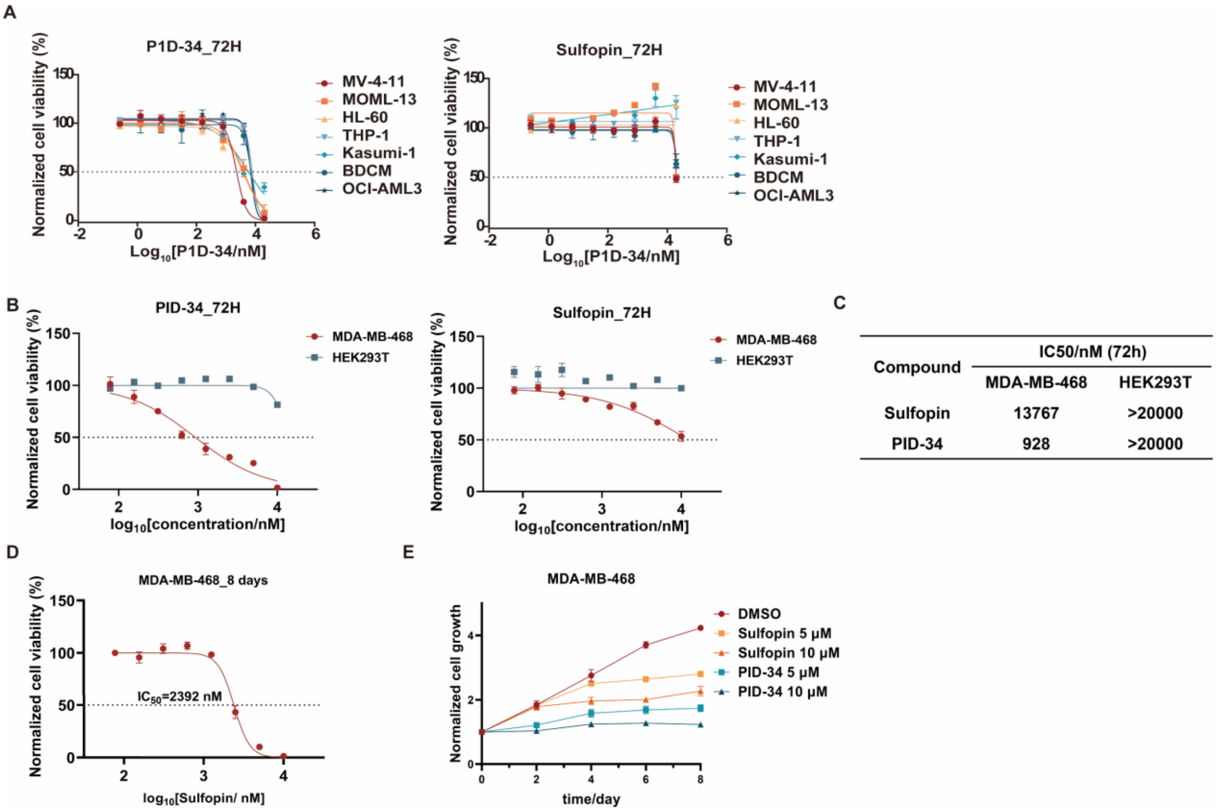

Compound	IC50/nM (72h)	
	MDA-MB-468	HEK293T
Sulfopin	13767	>20000
PID-34	928	>20000

Figure S4. (A) Cell growth inhibitory activities of **P1D-34** and Sulfopin in AML cell-lines. (B) Cell growth inhibitory activities of **P1D-34** and Sulfopin in MDA-MB-468 cells and HEK293T cells for 72 h. (C) Quantification of cell growth inhibitory activities in (B). (D) Cell growth inhibitory

activities of Sulfopin in MDA-MB-468 cells for 8 days. (E) Cell culture growth curves of PID-34 and Sulfopin in MDA-MB-468 cells (day 0-normalized growth rate for n= 3).

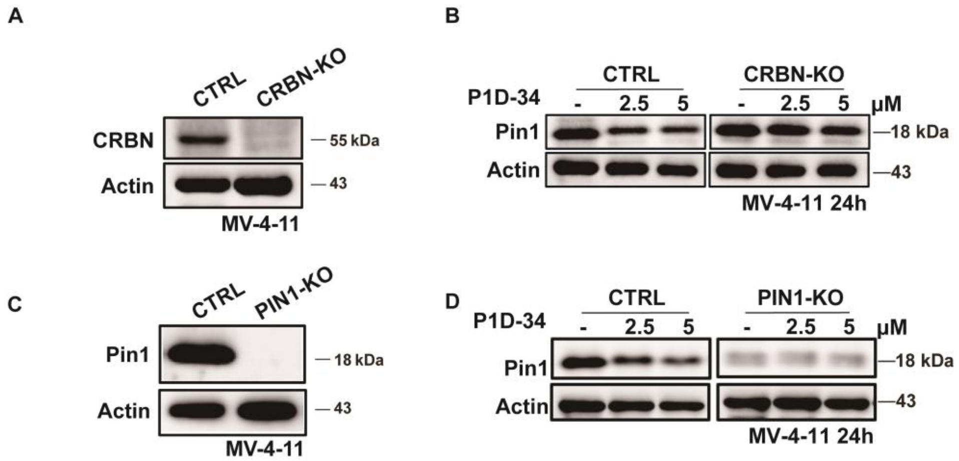

Figure S5. (A) Immunoblots for CRBN and Actin in MV-4-11 WT (CTRL) and CRBN knock-out cells. (B) Immunoblots of Pin1 in MV-4-11 CRBN-KO and CTRL cells treated with the indicated concentrations of P1D-34 for 24 h. (C) Immunoblots for Pin1 and Actin in MV-4-11 WT (CTRL) and Pin1 knock-out cells. (D) Immunoblots of Pin1 in MV-4-11 MV-4-11 WT (CTRL) and Pin1 knock-out cells treated with the indicated concentrations of P1D-34 for 24 h.

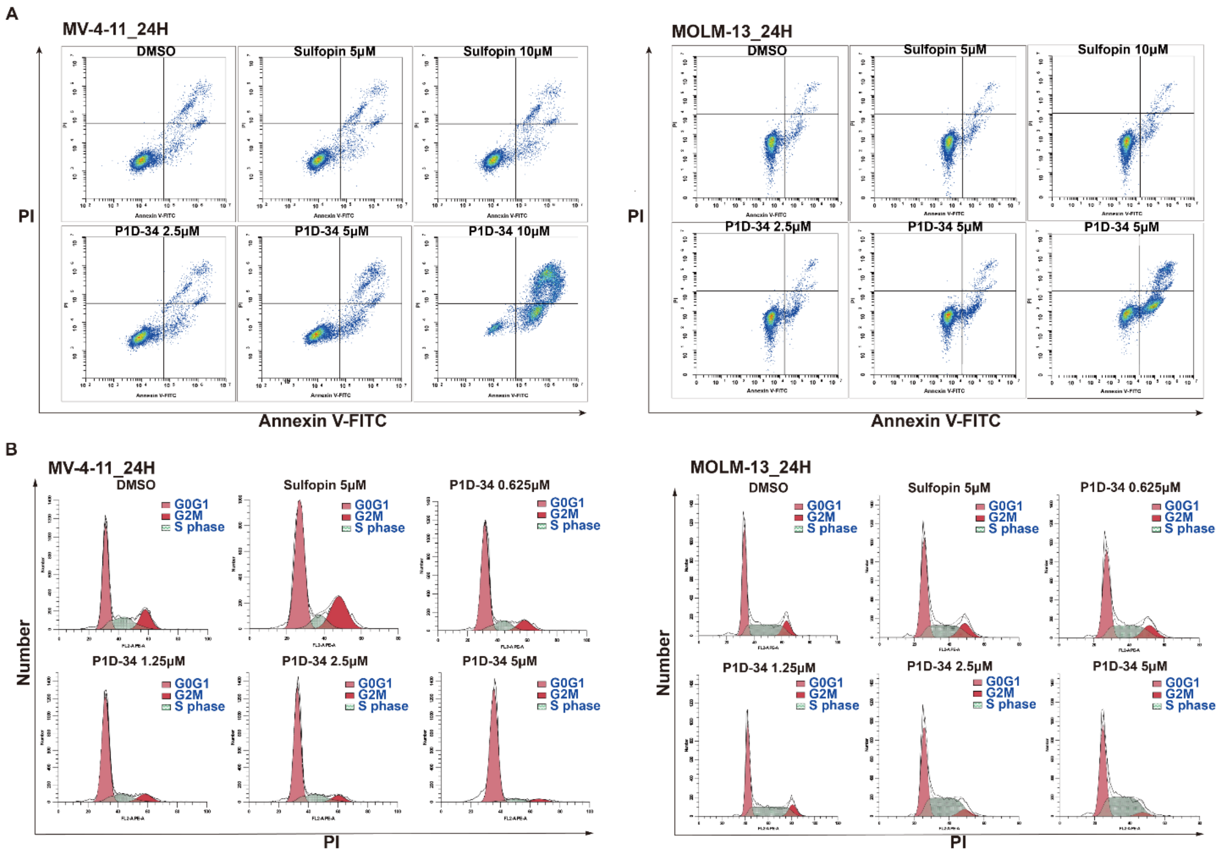

Figure S6. (A) Cell apoptosis and (B) cell cycle arrest effect in MV-4-11 and MOLM-13 cells induced by Sulfopin or **P1D-34**.

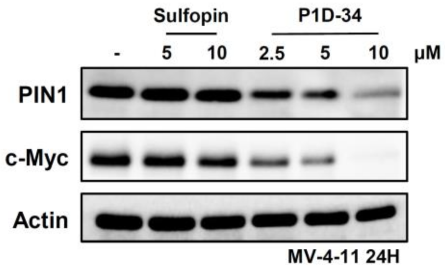

Figure S7. Immunoblots for Pin1 and c-Myc in MV-4-11 cells treated with the indicated concentrations of Sulfopin or P1D-34.

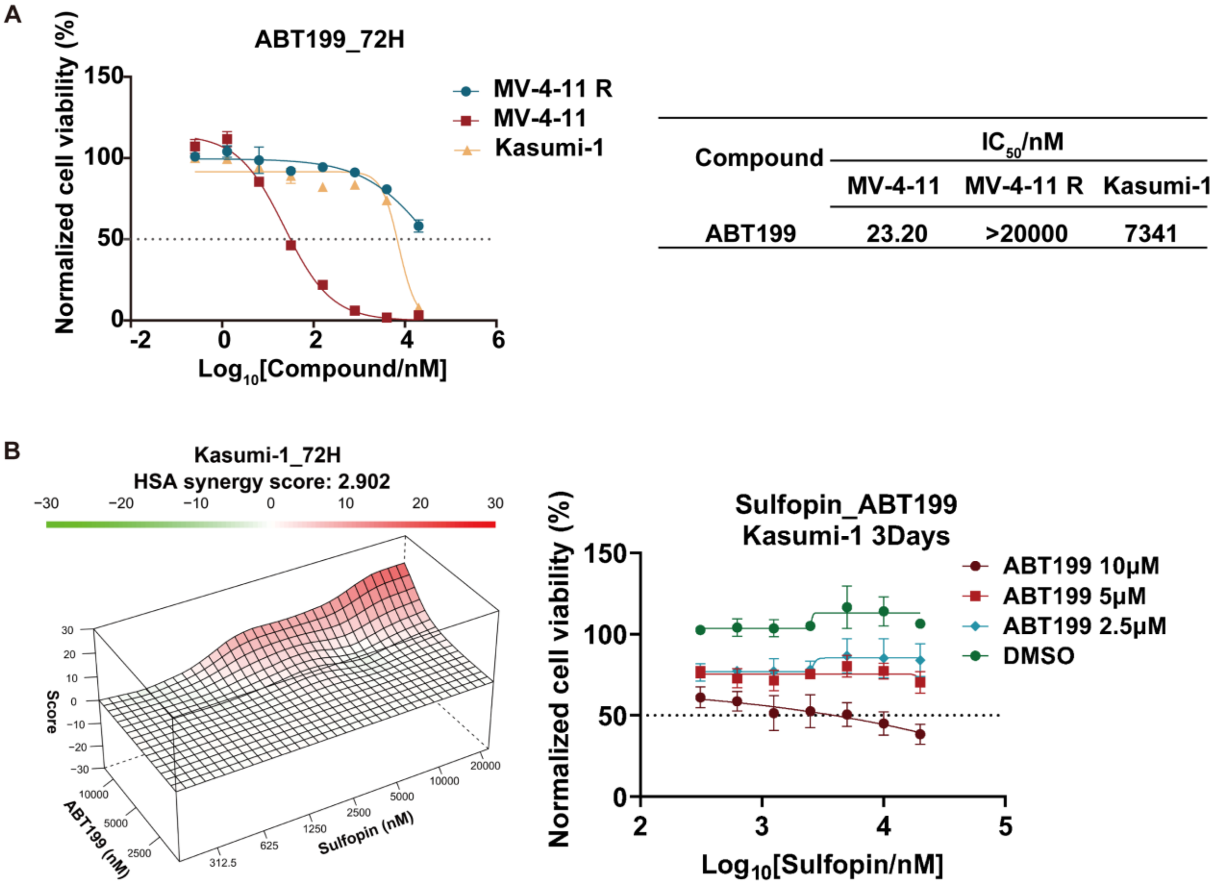

Figure S8. Cell growth inhibitory activities of ABT-199 in MV-4-11, MV-4-11 resistant (MV-4-11 R) and Kasumi-1 cells. (B) Excess over HSA synergy plots (left) and growth curves (right) for serial dilutions of Sulfopin in combination with ABT-199 in Kasumi-1 cell lines.

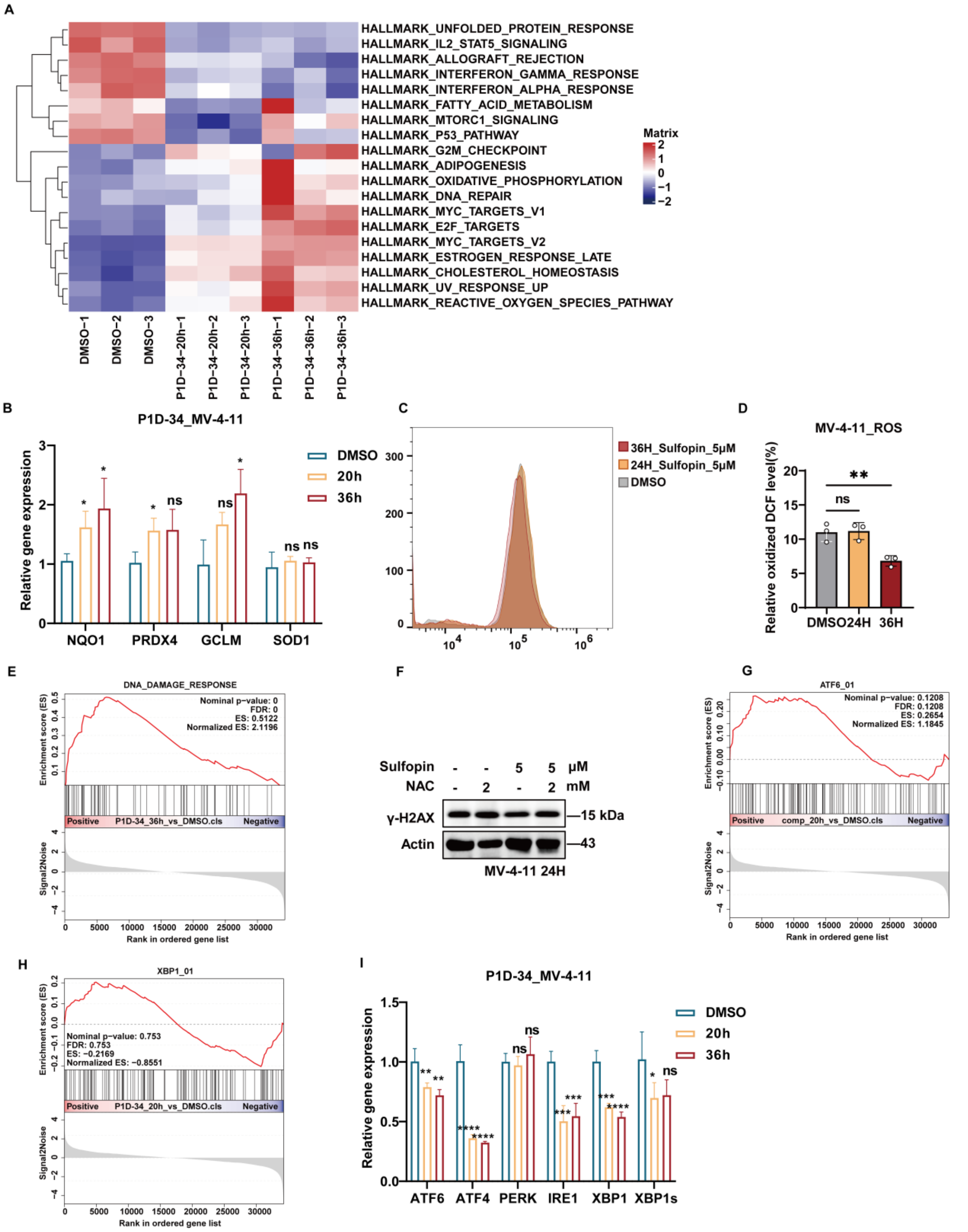

Figure S9. (A) Differentially activated gene ontological pathways by GSVA analysis. Heatmap comparison of DMSO and **P1D-34**. (B) Quantitative PCR was performed to confirm that ROS production related genes in MV-4-11 cells after treatment of **P1D-34** (5 µM). (C) Analysis of ROS production by flow cytometry in MV-4-11 cells treated with Sulfopin (5 µM) for 24 and 36 h. (D) Quantification of ROS production in (C), n = 3. Significance was analyzed by two-tailed t

test. *p < 0.05, **p < 0.01, ***p < 0.001, ****p < 0.0001. (E) Gene sets enrichment of DNA-DAMAGE-RESPONSE. (F) Immunoblots for γH2AX in MV-4-11 cells treated with Sulfopin and NAC for 24 h. (G) Gene sets enrichment of ATF6 gene sets. (H) Gene sets enrichment of XBP1 gene sets. (I) Quantitative PCR was performed to confirm that UPR related genes in MV-4-11 cells after treatment of **P1D-34** (5 μM).

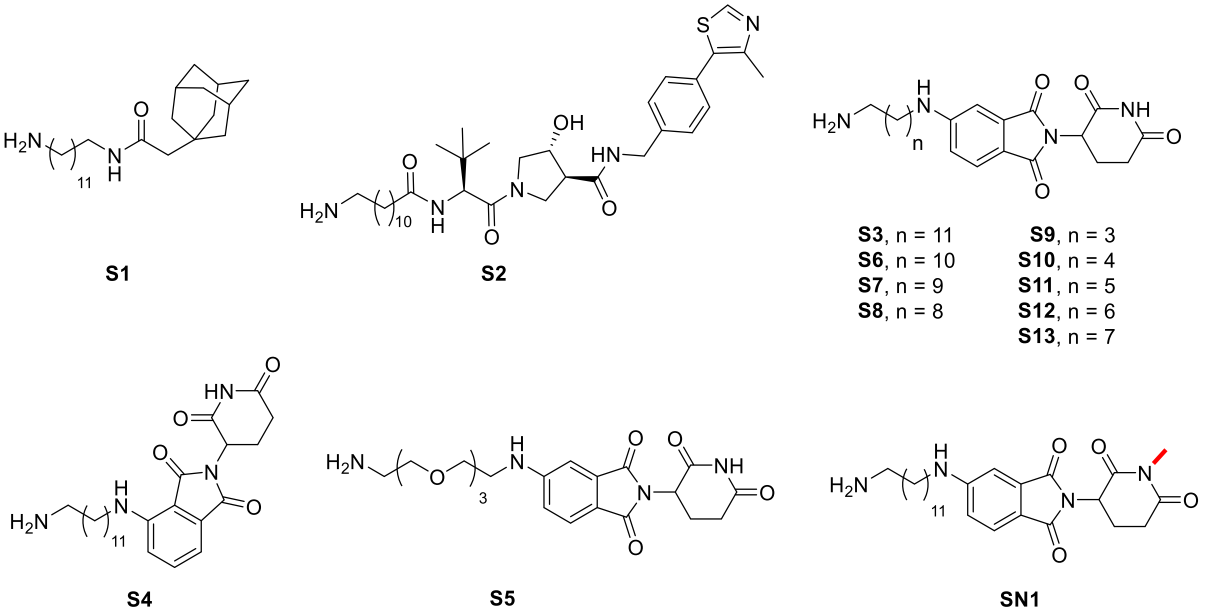

Figure S10. Chemical structures of **S1** – **S13** and **SN1**.

2. Supplementary Tables

Table S1. Pin1 degradation of Pin1 degraders[a].

Compound	Degradation Percentage (%, 24h)		P value (vs DMSO)	
	2 μM	20 μM	2 μM	20 μM
1	28	65	0.31389	0.23112
2	35	55	0.00239	0.01041
3 (PID-34)	72	88	0.00071	0.00015
4	57	60	0.00003	0.00990
5	10	53	0.48063	0.00473
6	63	49	0.00010	0.04718
7	61	70	0.00743	0.00088
8	63	68	0.00036	0.00084
9	51	57	0.00211	0.00795
10	43	63	0.00971	0.00037
11	60	60	0.00009	0.00099
12	46	57	0.00874	0.01165
13	56	69	0.00385	0.00022

a. The data are averages of three independent determinations.

Table S2. Pin1 degradation of the indicated concentration of P1D-34 for 24h.

Compound	Concentration (μM)	Degradation Percentage (%, 24h)
DMSO	-	0
PID-34	0.156	61
PID-34	0.625	72
PID-34	2.5	86
PID-34	10	90
PID-34	40	76

Table S3. Pin1 degradation of 5 μM P1D-34 at indicated time points.

Compound	Time	Degradation Percentage (%, 24h)
PID-34	0	4
PID-34	2	10
PID-34	4	-4
PID-34	8	17
PID-34	12	30
PID-34	16	56
PID-34	18	66
PID-34	20	70
PID-34	22	76
PID-34	24	79

Table S4. Pin1 degradation in MV-4-11 cells pre-treated with DMSO, Suofopin (10 µM), Thalidomide (10 µM), MLN-4924 (250 nM), or MG132 (5 µM) for 2 h, and then treated with 5 µM of P1D-34 for 20 h.

Compound	Degradation Percentage (%, 24h)
DMSO	0
DMSO- PID-34	45
Thalidomide and PID-34	13
Sulfopin and PID-34	-22
MLN4924 and PID-34	-4
MG132 and PID-34	12

Table S5. General analytical methods for degraders[a].

Method	Method
Method A	30% - 95% B (1 - 30 min), 95% - 100% B (30 - 35 min)
Method B	50% - 95% B (1 - 30 min), 95% - 100% B (30 - 35 min)
Method C	5% - 95% B (1 - 30 min), 95% - 100% B (30 - 35 min)
Method D	10% - 95% B (1 - 30 min), 95% - 100% B (30 - 35 min)

a. High-performance liquid chromatography (HPLC) spectra for compounds were acquired using a Shimadzu LC-20ADXR with a DGU-20A5R detector. Chromatography was performed on a Poroshell 120 HPH - C18 4.6 × 250 mm, 5 µm column with water containing 0.1% Trifluoroacetic acid (TFA) as solvent A and acetonitrile as solvent B at a flow rate of 0.600 mL/min.

3. Experimental Section - chemistry

Unless otherwise indicated, all commercial reagents and solvents were used directly without further purification. Reactions were monitored using a Waters Acquity UPLC/MS system (Waters PDA el Detector, QDa Detector, Sample manager - FL, Binary Solvent Manager) using Acquity UPLC® BEH C18 column (2.1 × 50 mm, 1.7 mm particle size): solvent gradient = 90% A at 0 min, 0% A at 4 min; solvent A = 0.1% formic acid in Water; solvent B = 0.1% formic acid in Acetonitrile; flow rate: 0.5 mL/min. Biotage automatic purification system was employed for the preparation of the intermediates. Products were purified by preparative HPLC using Waters SunFireTM Prep C18 column (19 × 100 mm, 5 mm particle size) using a gradient of 20 - 80% acetonitrile in water containing 0.05% TFA over 25 min at a flow of 45 mL/min. High-resolution mass spectra (HRMS) data were acquired in the positive ion mode using Agilent G6520 Q-TOF with an electrospray ionization (ESI) source. ^{1}H NMR and ^{13}C NMR spectra were performed on BRUKER AVANCE II 400M, BRUKER AVANCE III 500M or BRUKER AVANCE III 600M NMR spectrometer. ^{1}H NMR spectra are reported in ppm(δ) downfield from tetramethylsilane (TMS). All ^{13}C NMR spectra are reported in ppm and obtained with ^{1}H decoupling. Coupling constants are reported in Hz and multiplicities are quoted as singlet (s),doublet (d), triplet (t), quartet (q), heptet (h), multiplet (m) and broad signal (bs).

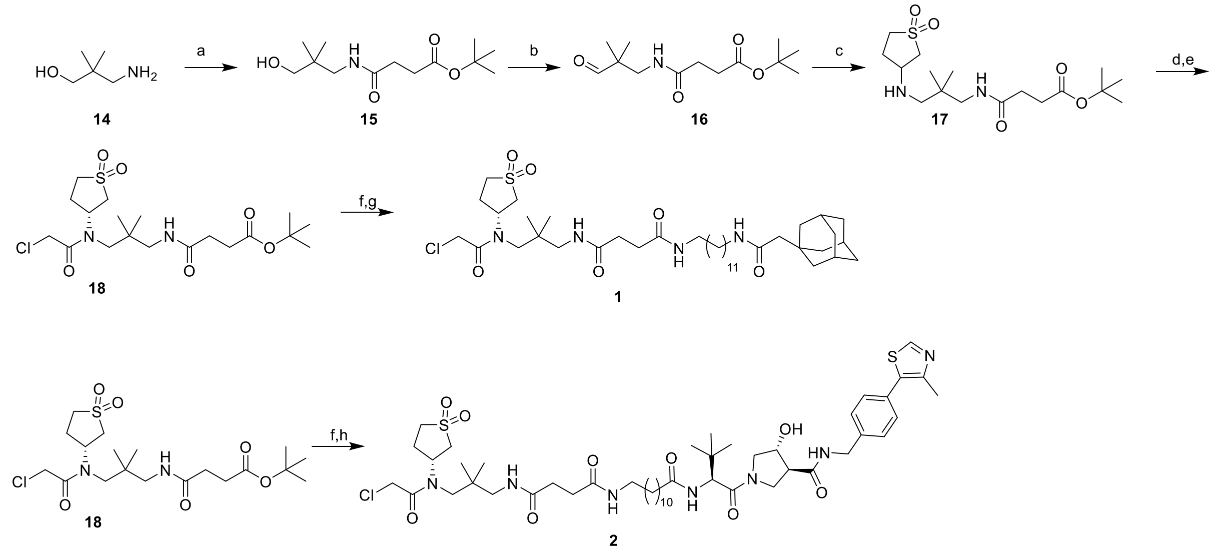

Scheme S1. Synthetic route of **1** and **2**

Reagents and conditions: (a) Mono-tert-butyl succinate, HATU, DIPEA, DMF, RT, 2 h; (b) Dess-Martin periodinane, DCM, RT, 6 h; (c) 3-aminotetrahydrothiopene 1,1-dioxide hydrochloride, $NaBH(OAc)_3$, DCE, RT, 12 h; (d) Chloroacetyl chloride, TEA, DCM, 0 ℃, 1 h; (e) Chiral SFC separation; (f) TFA, DCM, RT, 2 h; (g) **S1**, HATU, DIPEA, DMF, RT, 2 h; (h) **S2**, HATU, DIPEA, DMF, RT, 2 h.

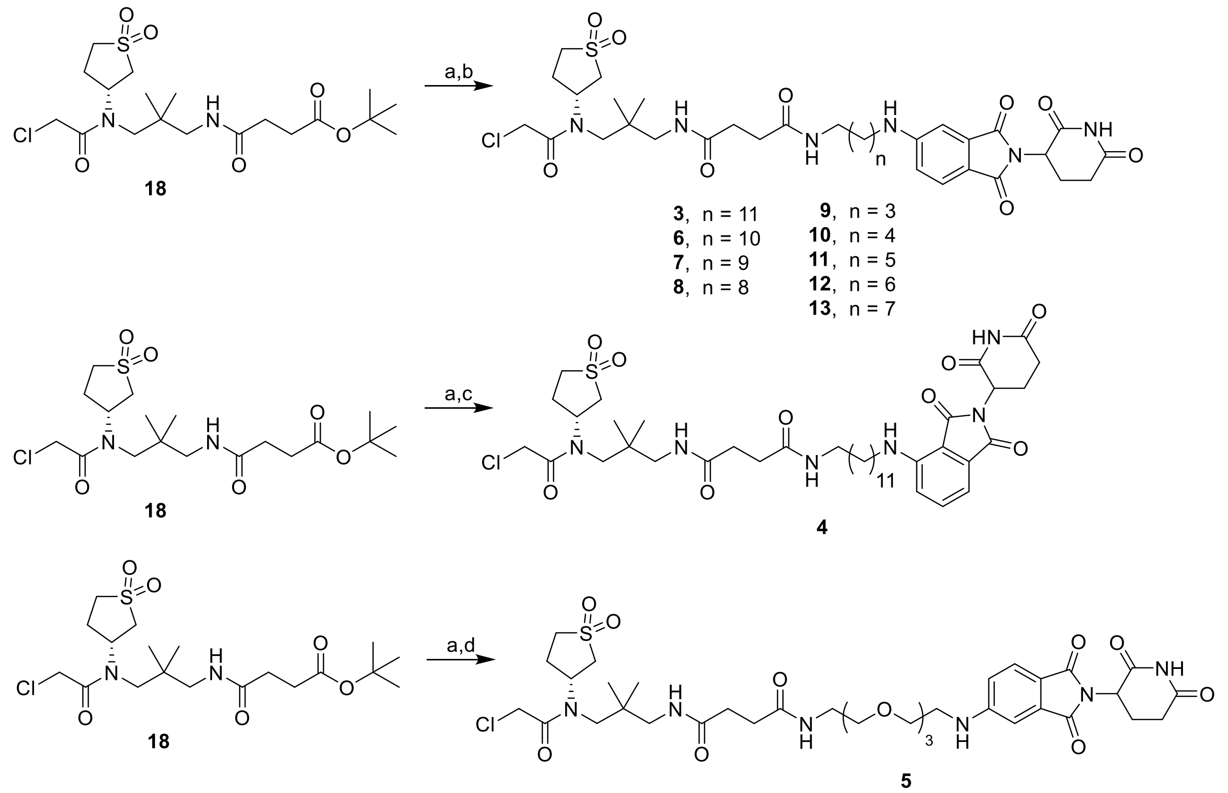

Scheme S2. Synthetic route of **3** - **13**

Reagents and conditions: (a) TFA, DCM, RT, 2 h; (b) **S3** or **S6** or **S7** or **S8** or **S9** or **S10** or **S11** or **S12** or **S13**, HATU, DIPEA, DMF, RT, 2 h; (c) **S4**, HATU, DIPEA, DMF, RT, 2 h; (d) **S5**, HATU, DIPEA, DMF, RT, 2 h.

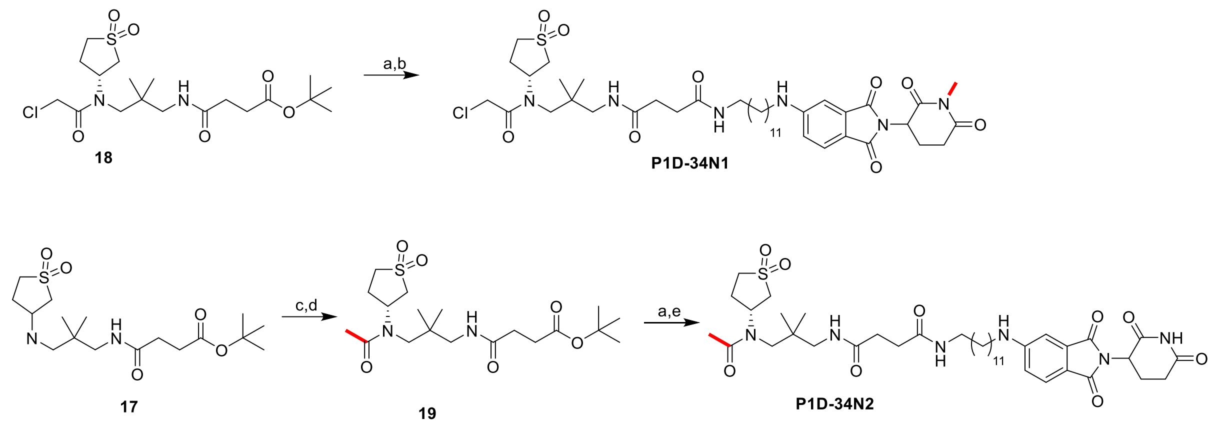

Scheme S3. Synthetic route of **P1D-34N1** and **P1D-34N2**

Reagents and conditions: (a) TFA, DCM, RT, 2 h; (b) **SN1**, HATU, DIPEA, DMF, RT, 2 h; (c) Acetic chloride, TEA, DCM, 0 ℃, 1 h; (d) Chiral SFC separation; (e) **S3**, HATU, DIPEA, DMF, RT, 2 h.

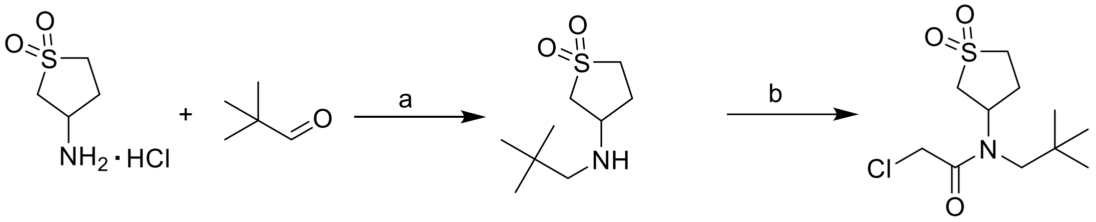

20 **21** **Sulfopin**

Scheme S4. Synthetic route of **Sulfopin**

Reagents and conditions: (a) STAB, TEA, HOAC, DMF, RT, 12 h; (b) Chloroacetyl chloride, TEA, DCM, 0 ℃ - RT, 2 h.

3.1. Chemical Synthesis of compound 1 – 13, P1D-34N1 ,P1D-34N2 and Sulfopin.

Synthesis of the intermediate *tert-butyl 4-((3-hydroxy-2,2-dimethylpropyl)amino)-4-oxobutanoate* (**15**).

A mixture of 3-amino-2,2-dimethyl-1-propanol (1.03 g, 10 mmol), mono-tert-Butyl succinate (1.74 g, 10 mmol) and HATU (3.8 g, 10 mmol) in DMF (10 ml) was stirred at room temperature and then DIPEA (2.6 ml, 15 mmol) was added. The solution was stirred at room temperature for 2 h, the DMF was evaporated and the residue was partitioned between ethyl acetate and aqueous sodium bicarbonate. The organic layer was washed with 0.5 M HCl, water and saturated brine and then dried ($MgSO_4$) and concentrated. The residue was purified by flash chromatography to afford the product **15** as a white solid (2.07 g, 80% yield). ^{1}H NMR (500 MHz, Chloroform-*d*) δ 6.40 – 6.28 (m, 1H), 4.02 – 3.90 (m, 1H), 3.11 (d, *J* = 5.9 Hz, 2H), 3.06 (d, *J* = 6.8 Hz, 2H), 2.57 (t, *J* = 6.6 Hz, 2H), 2.44 (t, *J* = 6.6 Hz, 2H), 1.42 (s, 9H), 0.85 (s, 6H). ^{13}C NMR (126 MHz, Chloroform-*d*) δ 173.69, 172.32, 81.00, 67.95, 46.43, 36.83, 31.21, 30.96, 28.04, 22.61. UPLC-MS (ESI) for $[M+H]^+$ 260.18.

Synthesis of the intermediate *tert-butyl 4-((2,2-dimethyl-3-oxopropyl)amino)-4-oxobutanoate* (**16**).

To a solution of **15** (1.55 g, 6 mmol) in dichloromethane (10 mL) was added solution of Dess−Martin−periodinane (2.5 g, 6 mmol, in 20 mL of dichloromethane). The reaction mixture was stirred at room temperature for 5 h. The solution was treated with sodium thiosulfate (20 mL, 0.5 M; saturated with sodium bicarbonate) until the organic layer become clear, then washed the organic layer with water (20 mL) followed by a saturated aqueous solution of sodium chloride (20 mL). The residue was purified by flash chromatography to afford the product **16** as a white solid (1.33 g, 86% yield). ^{1}H NMR (500 MHz, Chloroform-*d*) δ 9.42 (s, 1H), 3.34 (d, *J* = 6.5 Hz, 2H), 2.53 (t, *J* = 6.7 Hz, 2H), 2.38 (t, *J* = 6.8 Hz, 2H), 1.42 (s, 9H), 1.07 (s, 6H). ^{13}C NMR (126 MHz, Chloroform-*d*) δ 205.30, 172.24, 171.96, 80.80, 47.50, 44.15, 31.25, 30.82, 28.04, 19.61. UPLC-MS (ESI) for $[M+H]^+$ 258.18.

Synthesis of the intermediate *tert-butyl 4-((3-((1,1-dioxidotetrahydrothiophen-3-yl)amino)-2,2-dimethylpropyl)amino)-4-oxobutanoate* (**17**).

To a solution of **16** (1.29 g, 5 mmol) in DCE (8mL) was added 3-aminotetrahydrothiophene1,1-dioxidehydrochloride (858 mg, 5 mmol), sodium

triacetoxyborohydride (3.18 g, 15 mmol). The reaction mixture was stirred at room temperature for 1 day, then was quenched with saturated aqueous $NaHCO_3$ solution (10mL). The aqueous mixture was extracted with EtOAc (3 × 10 mL). The combined organic layers were washed with H_2O (3 × 10 mL) and brine. The residue was purified by flash chromatography to afford the product **17** as a white solid (2.8 g, 67% yield). ^{1}H NMR (500 MHz, Chloroform-*d*) δ 6.53 (s, 1H), 3.55 – 3.47 (m, 1H), 3.36 – 3.30 (m, 1H), 3.23 – 3.14 (m, 2H), 3.09 – 2.98 (m, 3H), 2.60 – 2.53 (m, 2H), 2.44 – 2.37 (m, 3H), 2.36 – 2.31 (m, 2H), 2.20 – 2.08 (m, 1H), 1.43 (s, 9H), 0.90 (s, 6H). ^{13}C NMR (126 MHz, Chloroform-*d*) δ 172.50, 172.28, 80.73, 56.84, 56.14, 55.80, 50.39, 47.58, 35.26, 31.36, 30.96, 29.79, 28.11, 24.33, 24.20. UPLC-MS (ESI) for $[M+H]^+$ 377.21.

Synthesis of the intermediate *tert-butyl (R)-4-((3-(2-chloro-N-(1,1-dioxidotetrahydrothiophen-3-yl)acetamido)-2,2-dimethylpropyl)amino)-4-oxobutanoate* (**18**).

To a solution of **17** (1.88 g, 5 mmol) in DCM (10 mL) was added chloroacetyl chloride (398 µL, 5 mmol), TEA (837 µL, 6 mmol) at 0 ℃. The reaction mixture was stirred at room temperature for 1 h. The aqueous mixture was extracted with DCM (3 × 10mL). The combined organic layers were washed with H_2O (3 × 10mL) and brine (10mL). The residue was purified by flash chromatography to afford the racemic product as a white solid (2.08 g, 92% yield). Then the product **18** was obtained though chiral SFC separation (Column: Chiralpak AD-3 50 x 4.6mm I.D., 3um; Mobile phase: A: CO_2 B: ethanol (0.05% DEA); Gradient: from 5% to 40% of B in 4 min and from 40% to 5% of B in 0.2 min, then hold 5% of B for 1.8 min; Flow rate: 3 mL/min). ^{1}H NMR (500 MHz, Chloroform-*d*) δ 6.26 – 6.13 (m, 1H), 4.17 – 4.06 (m, 2H), 3.97 – 3.86 (m, 1H), 3.74 – 3.62 (m, 2H), 3.34 (d, *J* = 15.8 Hz, 1H), 3.22 (d, *J* = 15.7 Hz, 1H), 3.17 – 3.09 (m, 3H), 3.06 – 2.98 (m, 1H), 2.62 – 2.57 (m, 2H), 2.54 – 2.46 (m, 2H), 2.45 – 2.38 (m, 2H), 1.42 (s, 9H), 0.98 (s, 6H). ^{13}C NMR (126 MHz, Chloroform-*d*) δ 172.77, 172.59, 167.95, 81.10, 58.48, 57.66, 50.40, 49.10, 48.20, 42.29, 38.04, 31.30, 30.63, 28.07, 26.58, 23.87, 23.80. UPLC-MS (ESI) for $[M+H]^+$ 453.18.

Synthesis of the intermediate *tert-butyl (R)-4-((3-(N-(1,1-dioxidotetrahydrothiophen-3-yl)acetamido)-2,2-dimethylpropyl)amino)-4-oxobutanoate* (**19**)

To a solution of **17** (188 mg, 0.5 mmol) in DCM (3 mL) was added acetyl chloride (35.7 µL, 0.5 mmol), TEA (83.7 µL, 0.6 mmol) at 0 ℃. The reaction mixture was stirred at room temperature for 1 h. The aqueous mixture was extracted with DCM (3 × 10mL). The combined

organic layers were washed with H_2O (3 × 10mL) and brine (10mL). The residue was purified by flash chromatography to afford the racemic product as a white solid (188 mg, 90% yield). Then the product **19** was obtained through chiral SFC separation. The separation condition is the same as that used for **18**. ^{1}H NMR (600 MHz, Chloroform-*d*) δ 6.20 – 6.09 (m, 1H), 3.91 – 3.82 (m, 1H), 3.81 – 3.73 (m, 1H), 3.71 – 3.62 (m, 1H), 3.36 – 3.30 (m, 1H), 3.24 – 3.13 (m, 3H), 3.13 – 3.07 (m, 1H), 3.05 – 2.98 (m, 1H), 2.63 – 2.58 (m, 2H), 2.57 – 2.47 (m, 2H), 2.48 – 2.41 (m, 2H), 2.11 (s, 3H), 1.44 (s, 9H), 0.99 (s, 6H). ^{13}C NMR (126 MHz, Chloroform-*d*) δ 172.21, 172.00, 171.83, 80.57, 58.87, 56.57, 50.07, 48.96, 47.90, 37.72, 30.88, 30.23, 27.62, 26.43, 23.38, 23.36, 22.94. UPLC-MS (ESI) for $[M+H]^+$ 419.22.

Compounds **1** - **13**, **P1D-34N1** and **P1D-34N2** were synthesized according to the following procedure: Compound **18** or **19** (0.2 mmol) was dissolved in TFA (1 ml) and DCM (3 ml) and the mixture was stirred at room temperature for 2 h. The reaction was then concentrated under reduced pressure to give the intermediate acid without further purification. Then this intermediate, HATU (0.2 mmol), DIPEA (0.3 mmol), and the corresponding amine (0.3 mmol) was dissolved in DMF (2 ml), and the mixture was stirred at room temperature for 2 h. Then the reaction was partitioned between ethyl acetate and aqueous sodium bicarbonate. The organic layer was washed with 0.5 M HCl, water and saturated brine and then dried ($MgSO_4$) and concentrated. The residue was purified by HPLC (mobile phase: CH_3CN containing 0.1% TFA and H_2O containing 0.1% TFA) to afford the product **1 - 13, P1D-34N1** and **P1D-34N2**.

N^1-(12-(2-((3r,5r,7r)-adamantan-1-yl)acetamido)dodecyl)-N^4-(3-(2-chloro-N-((R)-1,1-dioxidotetrahydrothiophen-3-yl)acetamido)-2,2-dimethylpropyl)succinamide (**1**)

White solid (80% yield). ^{1}H NMR (500 MHz, DMSO-d_6) δ 7.81 (t, *J* = 6.2 Hz, 1H), 7.75 (t, *J* = 5.6 Hz, 1H), 7.59 (t, *J* = 5.7 Hz, 1H), 4.41 – 4.27 (m, 2H), 4.03 – 3.93 (m, 1H), 3.52 – 3.41 (m, 2H), 3.27 – 3.19 (m, 2H), 3.12 – 3.06 (m, 1H), 3.03 – 2.91 (m, 6H), 2.51 – 2.49 (m, 1H), 2.43 – 2.28 (m, 6H), 1.92 – 1.87 (m, 3H), 1.82 – 1.78 (m, 2H), 1.67 – 1.62 (m, 3H), 1.58 – 1.53 (m, 9H), 1.38 – 1.33 (m, 4H), 1.26 – 1.20 (m, 16H), 0.88 (s, 6H). ^{13}C NMR (126 MHz, DMSO-d_6) δ 172.41, 171.58, 170.08, 167.95, 57.74, 57.60, 51.26, 50.58, 50.03, 47.35, 43.43, 42.62, 38.96, 38.67, 37.84, 36.95, 32.61, 31.40, 31.28, 29.64, 29.62, 29.49, 29.43, 29.25, 29.18, 28.54, 26.89, 26.63, 23.79, 23.70. HPLC > 95%, t_R = 19.809 min (method B); HRMS (ESI-TOF) m/z: $[M + H]^+$ calcd for $C_{39}H_{68}ClN_4O_6S^+$, 755.4543; found, 755.4540.

N[1]*-(3-(2-chloro-N-((R)-1,1-dioxidotetrahydrothiophen-3-yl)acetamido)-2,2-dimethylpropyl)-N*[4]*-(12-(((S)-1-((3R,4S)-3-hydroxy-4-((4-(4-methylthiazol-5-yl)benzyl)carbamoyl)pyrrolidin-1-yl)-3,3-dimethyl-1-oxobutan-2-yl)amino)-12-oxododecyl)succinamide* (**2**)

White solid (88% yield). ^{1}H NMR (600 MHz, Chloroform-*d*) δ 8.99 – 8.79 (m, 1H), 7.42 – 7.35 (m, 5H), 7.19 (t, *J* = 6.3 Hz, 1H), 6.37 – 6.29 (m, 1H), 6.28 – 6.21 (m, 1H), 4.74 – 4.68 (m, 1H), 4.61 – 4.50 (m, 3H), 4.40 – 4.32 (m, 1H), 4.19 – 4.09 (m, 2H), 3.99 – 3.90 (m, 2H), 3.75 – 3.58 (m, 3H), 3.39 – 3.29 (m, 1H), 3.26 – 3.07 (m, 6H), 3.06 – 2.99 (m, 1H), 2.56 – 2.50 (m, 8H), 2.48 – 2.43 (m, 1H), 2.23 – 2.13 (m, 3H), 1.64 – 1.52 (m, 2H), 1.49 – 1.42 (m, 2H), 1.28 – 1.24 (m, 12H), 0.99 (d, *J* = 3.6 Hz, 6H), 0.95 (s, 9H). ^{13}C NMR (126 MHz, Chloroform-*d*) δ 173.86, 173.12, 172.32, 171.26, 170.70, 167.61, 151.40, 145.67, 138.59, 132.71, 129.41, 128.99, 127.78, 69.59, 58.51, 57.72, 57.17, 57.10, 56.51, 50.02, 48.61, 47.63, 42.69, 41.83, 39.42, 37.24, 35.90, 34.70, 31.31, 31.01, 29.32, 28.86, 28.79, 28.70, 28.66, 28.61, 28.51, 26.75, 26.21, 26.07, 25.95, 25.09, 23.45, 14.38. HPLC > 95%, t_R = 14.888 min (method A); HRMS (ESI-TOF) m/z: [M + H]$^+$ calcd for $C_{49}H_{77}ClN_7O_9S_2^+$, 1006.4907; found, 1006.4905.

N[1]*-(3-(2-chloro-N-((R)-1,1-dioxidotetrahydrothiophen-3-yl)acetamido)-2,2-dimethylpropyl)-N*[4]*-(12-((2-(2,6-dioxopiperidin-3-yl)-1,3-dioxoisoindolin-5-yl)amino)dodecyl)succinamide* (**3**)

Yellow solid (84% yield). ^{1}H NMR (500 MHz, Methanol-d_4) δ 7.55 (d, *J* = 8.4 Hz, 1H), 6.97 (d, *J* = 2.2 Hz, 1H), 6.82 (dd, *J* = 8.4, 2.2 Hz, 1H), 5.03 (dd, *J* = 12.7, 5.5 Hz, 1H), 4.32 – 4.21 (m, 2H), 4.15 – 3.98 (m, 1H), 3.68 – 3.49 (m, 2H), 3.40 – 3.32 (m, 2H), 3.23 – 3.17 (m, 2H), 3.16 – 3.04 (m, 5H), 2.90 – 2.80 (m, 1H), 2.78 – 2.65 (m, 2H), 2.57 – 2.51 (m, 2H), 2.49 (s, 4H), 2.13 – 2.06 (m, 1H), 1.71 – 1.59 (m, 2H), 1.52 – 1.40 (m, 4H), 1.37 – 1.26 (m, 16H), 0.98 (s, 6H). ^{13}C NMR (126 MHz, Methanol-d_4) δ 173.90, 173.30, 172.99, 170.37, 168.97, 168.29, 167.93, 154.79, 134.55, 124.83, 116.54, 115.19, 105.34, 57.90, 57.82, 50.52, 49.17, 48.90, 42.78, 42.08, 39.10, 37.50, 30.81, 30.59, 29.21, 29.02, 28.99, 28.42, 26.68, 26.57, 26.12, 22.66, 22.46. HPLC > 95%, t_R = 20.200 min (method A); HRMS (ESI-TOF) m/z: [M + H]$^+$ calcd for $C_{40}H_{60}ClN_6O_9S^+$, 835.3826; found, 835.3832.

N[1]*-(3-(2-chloro-N-((R)-1,1-dioxidotetrahydrothiophen-3-yl)acetamido)-2,2-dimethylpropyl)-N*[4]*-(12-((2-(2,6-dioxopiperidin-3-yl)-1,3-dioxoisoindolin-4-yl)amino)dodecyl)succinamide* (**4**)

Yellow solid (87% yield). ^{1}H NMR (500 MHz, Methanol-d_4) δ 7.54 (t, *J* = 7.8 Hz, 1H), 7.03 (d, *J* = 7.3 Hz, 2H), 5.05 (dd, *J* = 12.4, 5.5 Hz, 1H), 4.32 – 4.22 (m, 2H), 4.12 – 4.01 (m, 1H), 3.67 – 3.50 (m, 2H), 3.37 (d, *J* = 15.9 Hz, 1H), 3.33 (d, *J* = 4.7 Hz, 3H), 3.17 – 3.02 (m, 5H), 2.91 – 2.80 (m, 1H), 2.79 – 2.66 (m, 2H), 2.58 – 2.47 (m, 6H), 2.15 – 2.06 (m, 1H), 1.69 – 1.61 (m, 2H), 1.51 – 1.40 (m, 4H), 1.39 – 1.26 (m, 16H), 0.98 (s, 6H). ^{13}C NMR (126 MHz, Methanol-d_4) δ 173.90, 173.26, 172.99, 170.27, 169.40, 168.96, 167.94, 146.91, 135.83, 132.51, 116.60, 110.33, 109.56, 57.91, 57.82, 50.53, 49.18, 48.79, 42.09, 42.02, 39.11, 37.51, 30.83, 30.60, 29.22, 29.18, 29.04, 29.00, 28.98, 28.88, 26.58, 26.52, 26.12, 22.66, 22.41. HPLC > 95%, t_R = 15.267 min (method B); HRMS (ESI-TOF) m/z: [M + H]$^+$ calcd for $C_{40}H_{60}ClN_6O_9S^+$, 835.3826; found, 835.3826.

N^1-(3-(2-chloro-N-((R)-1,1-dioxidotetrahydrothiophen-3-yl)acetamido)-2,2-dimethylpropyl)-N^4-(2-(2-(2-(2-((2-(2,6-dioxopiperidin-3-yl)-1,3-dioxoisoindolin-5-yl)amino)ethoxy)ethoxy)ethoxy)ethyl)succinimide (**5**)

Yellow solid (69% yield). ^{1}H NMR (500 MHz, Chloroform-*d*) δ 9.24 (s, 1H), 7.57 (d, *J* = 8.3 Hz, 1H), 7.07 – 6.98 (m, 2H), 6.91 (t, *J* = 5.2 Hz, 1H), 6.76 (dd, *J* = 8.3, 2.2 Hz, 1H), 4.99 – 4.89 (m, 1H), 4.18 – 4.06 (m, 2H), 3.96 – 3.88 (m, 1H), 3.86 – 3.78 (m, 2H), 3.75 – 3.71 (m, 2H), 3.68 – 3.60 (m, 9H), 3.56 – 3.51 (m, 2H), 3.44 – 3.36 (m, 4H), 3.34 – 3.19 (m, 2H), 3.18 – 2.96 (m, 4H), 2.89 – 2.80 (m, 1H), 2.78 – 2.69 (m, 2H), 2.59 – 2.42 (m, 6H), 2.19 – 2.06 (m, 1H), 0.95 (s, 6H). ^{13}C NMR (126 MHz, Chloroform-*d*) δ 173.53, 172.87, 171.80, 169.35, 168.19, 168.05, 167.46, 153.99, 134.39, 125.62, 117.94, 115.78, 107.08, 70.38, 70.18, 70.06, 69.55, 68.89, 58.14, 57.57, 50.50, 49.09, 48.08, 43.02, 42.41, 39.45, 37.73, 31.66, 31.46, 31.23, 26.55, 23.83, 22.80. HPLC > 95%, t_R = 17.051 min (method C); HRMS (ESI-TOF) m/z: [M + H]$^+$ calcd for $C_{36}H_{52}ClN_6O_{12}S^+$, 827.3047; found, 827.3047.

N^1-(3-(2-chloro-N-((R)-1,1-dioxidotetrahydrothiophen-3-yl)acetamido)-2,2-dimethylpropyl)-N^4-(11-((2-(2,6-dioxopiperidin-3-yl)-1,3-dioxoisoindolin-5-yl)amino)undecyl)succinimide (**6**)

Yellow solid (73% yield). ^{1}H NMR (500 MHz, Methanol-d_4) δ 7.55 (d, *J* = 8.4 Hz, 1H), 6.96 (d, *J* = 2.1 Hz, 1H), 6.82 (dd, *J* = 8.4, 2.1 Hz, 1H), 5.04 (dd, *J* = 12.6, 5.5 Hz, 1H), 4.32 – 4.22 (m, 2H), 4.12 – 4.03 (m, 1H), 3.68 – 3.49 (m, 2H), 3.41 – 3.33 (m, 2H), 3.22 – 3.17 (m, 2H), 3.16 – 3.03 (m, 5H), 2.90 – 2.81 (m, 1H), 2.78 – 2.65 (m, 2H), 2.58 – 2.47 (m, 6H), 2.13 – 2.05 (m, 1H), 1.69 – 1.61 (m, 2H), 1.50 – 1.41 (m, 4H), 1.39 – 1.23 (m, 14H), 0.98 (s, 6H). ^{13}C NMR (126 MHz,

Methanol-d_4) δ 173.45, 172.86, 172.53, 169.94, 168.51, 167.83, 167.46, 154.32, 134.10, 124.38, 116.07, 114.75, 104.89, 57.43, 57.36, 50.07, 48.71, 48.44, 42.32, 41.65, 38.64, 37.06, 30.36, 30.12, 28.75, 28.70, 28.58, 28.53, 27.96, 26.23, 26.11, 25.67, 22.20, 22.00. HPLC > 95%, t_R = 10.333 min (method B); HRMS (ESI-TOF) m/z: $[M + H]^+$ calcd for $C_{39}H_{58}ClN_6O_9S^+$, 821.3669; found, 821.3667.

N^1-(3-(2-chloro-N-((R)-1,1-dioxidotetrahydrothiophen-3-yl)acetamido)-2,2-dimethylpropyl)-N^4-(10-((2-(2,6-dioxopiperidin-3-yl)-1,3-dioxoisoindolin-5-yl)amino)decyl)succinimide (**7**)

Yellow solid (85% yield). ^{1}H NMR (500 MHz, Methanol-d_4) δ 7.55 (d, J = 8.4 Hz, 1H), 6.97 (d, J = 2.2 Hz, 1H), 6.82 (dd, J = 8.4, 2.2 Hz, 1H), 5.03 (dd, J = 12.6, 5.5 Hz, 1H), 4.34 – 4.22 (m, 2H), 4.12 – 4.04 (m, 1H), 3.62 – 3.58 (m, 1H), 3.58 – 3.49 (m, 1H), 3.40 – 3.32 (m, 2H), 3.22 – 3.17 (m, 2H), 3.15 – 3.04 (m, 5H), 2.88 – 2.79 (m, 1H), 2.76 – 2.66 (m, 2H), 2.56 – 2.50 (m, 2H), 2.49 (s, 4H), 2.12 – 2.05 (m, 1H), 1.69 – 1.61 (m, 2H), 1.50 – 1.40 (m, 4H), 1.37 – 1.29 (m, 12H), 0.99 (s, 6H). ^{13}C NMR (126 MHz, Methanol-d_4) δ 173.89, 173.28, 172.97, 170.37, 168.96, 168.28, 167.92, 154.83, 134.57, 124.81, 116.51, 115.16, 105.29, 57.89, 57.82, 50.51, 49.17, 48.90, 42.74, 42.07, 39.07, 37.50, 30.81, 30.58, 29.13, 29.11, 29.02, 28.99, 28.94, 28.42, 26.66, 26.54, 26.12, 22.65, 22.45. HPLC > 95%, t_R = 17.014 min (method A); HRMS (ESI-TOF) m/z: $[M + H]^+$ calcd for $C_{38}H_{56}ClN_6O_9S^+$, 807.3513; found, 807.3519.

N^1-(3-(2-chloro-N-((R)-1,1-dioxidotetrahydrothiophen-3-yl)acetamido)-2,2-dimethylpropyl)-N^4-(9-((2-(2,6-dioxopiperidin-3-yl)-1,3-dioxoisoindolin-5-yl)amino)nonyl)succinimide (**8**)

Yellow solid (89% yield). ^{1}H NMR (500 MHz, Methanol-d_4) δ 7.55 (d, J = 8.4 Hz, 1H), 6.97 (d, J = 2.2 Hz, 1H), 6.82 (dd, J = 8.4, 2.2 Hz, 1H), 5.03 (dd, J = 12.6, 5.4 Hz, 1H), 4.32 – 4.22 (m, 2H), 4.11 – 4.02 (m, 1H), 3.69 – 3.49 (m, 2H), 3.40 – 3.32 (m, 2H), 3.20 (t, J = 7.1 Hz, 2H), 3.16 – 3.04 (m, 5H), 2.90 – 2.80 (m, 1H), 2.77 – 2.64 (m, 2H), 2.58 – 2.46 (m, 6H), 2.13 – 2.05 (m, 1H), 1.69 – 1.61 (m, 2H), 1.51 – 1.41 (m, 4H), 1.40 – 1.26 (m, 10H), 0.98 (s, 6H). ^{13}C NMR (126 MHz, Methanol-d_4) δ 173.90, 173.30, 172.99, 170.38, 168.97, 168.29, 167.93, 154.82, 134.56, 124.82, 116.52, 115.19, 105.32, 57.90, 57.82, 50.52, 49.17, 48.91, 42.75, 42.07, 39.06, 37.51, 30.81, 30.59, 29.12, 29.02, 28.95, 28.88, 28.42, 26.65, 26.52, 26.12, 22.66, 22.45. HPLC > 95%, t_R = 15.567 min (method A); HRMS (ESI-TOF) m/z: $[M + H]^+$ calcd for $C_{37}H_{54}ClN_6O_9S^+$, 793.3356; found, 793.3358.

N1-(3-(2-chloro-N-((R)-1,1-dioxidotetrahydrothiophen-3-yl)acetamido)-2,2-dimethylpropyl)-N4-(4-((2-(2,6-dioxopiperidin-3-yl)-1,3-dioxoisoindolin-5-yl)amino)butyl)succinamide （**9**）

Yellow solid (84% yield). ^{1}H NMR (600 MHz, Methanol-d_4) δ 7.57 (d, *J* = 8.4 Hz, 1H), 6.99 (d, *J* = 2.1 Hz, 1H), 6.85 (dd, *J* = 8.4, 2.2 Hz, 1H), 5.06 (dd, *J* = 12.8, 5.4 Hz, 1H), 4.34 – 4.24 (m, 2H), 4.16 – 3.99 (m, 1H), 3.68 – 3.60 (m, 1H), 3.59 – 3.52 (m, 1H), 3.44 – 3.34 (m, 4H), 3.28 – 3.21 (m, 4H), 3.16 – 3.05 (m, 3H), 2.93 – 2.83 (m, 1H), 2.80 – 2.67 (m, 2H), 2.60 – 2.45 (m, 6H), 2.16 – 2.09 (m, 1H), 1.73 – 1.59 (m, 4H), 1.04 – 0.91 (m, 6H). ^{13}C NMR (151 MHz, Methanol-d_4) δ 173.86, 173.32, 173.15, 170.38, 168.95, 168.25, 167.90, 154.68, 134.55, 124.87, 116.68, 115.26, 105.43, 57.91, 57.80, 50.51, 49.15, 48.92, 42.39, 42.09, 38.59, 37.52, 30.82, 30.70, 30.53, 26.62, 26.12, 25.65, 22.63, 22.44. HPLC > 95%, t_R = 15.997 min (method D); HRMS (ESI-TOF) m/z: [M + H]$^+$ calcd for $C_{32}H_{44}ClN_6O_9S^+$, 723.2574; found, 723.2574.

N1-(3-(2-chloro-N-((R)-1,1-dioxidotetrahydrothiophen-3-yl)acetamido)-2,2-dimethylpropyl)-N4-(5-((2-(2,6-dioxopiperidin-3-yl)-1,3-dioxoisoindolin-5-yl)amino)pentyl)succinamide (**10**)

Yellow solid (78% yield). ^{1}H NMR (600 MHz, Methanol-d_4) δ 7.57 (d, *J* = 8.4 Hz, 1H), 7.00 (d, *J* = 2.2 Hz, 1H), 6.85 (dd, *J* = 8.4, 2.2 Hz, 1H), 5.06 (dd, *J* = 12.8, 5.5 Hz, 1H), 4.36 – 4.22 (m, 2H), 4.14 – 4.02 (m, 1H), 3.69 – 3.60 (m, 1H), 3.60 – 3.52 (m, 1H), 3.41 – 3.32 (m, 4H), 3.25 – 3.17 (m, 4H), 3.15 – 3.05 (m, 3H), 2.93 – 2.83 (m, 1H), 2.81 – 2.66 (m, 2H), 2.61 – 2.48 (m, 6H), 2.16 – 2.08 (m, 1H), 1.72 – 1.65 (m, 2H), 1.61 – 1.53 (m, 2H), 1.51 – 1.43 (m, 2H), 1.05 – 0.92 (m, 6H). ^{13}C NMR (151 MHz, Methanol-d_4) δ 173.88, 173.35, 173.10, 170.41, 168.96, 168.28, 167.91, 154.71, 134.54, 124.86, 116.61, 115.25, 105.40, 57.91, 57.80, 50.51, 49.16, 48.91, 42.66, 42.12, 38.79, 37.54, 30.83, 30.69, 30.51, 28.78, 27.99, 26.14, 23.89, 22.64, 22.45. HPLC > 95%, t_R = 16.853 min (method D); HRMS (ESI-TOF) m/z: [M + H]$^+$ calcd for $C_{33}H_{46}ClN_6O_9S^+$, 737.2730; found, 737.2729.

N1-(3-(2-chloro-N-((R)-1,1-dioxidotetrahydrothiophen-3-yl)acetamido)-2,2-dimethylpropyl)-N4-(6-((2-(2,6-dioxopiperidin-3-yl)-1,3-dioxoisoindolin-5-yl)amino)hexyl)succinamide (**11**)

Yellow solid (67% yield). ^{1}H NMR (600 MHz, Methanol-d_4) δ 7.57 (d, *J* = 8.4 Hz, 1H), 6.99 (d, *J* = 2.1 Hz, 1H), 6.85 (dd, *J* = 8.4, 2.2 Hz, 1H), 5.06 (dd, *J* = 12.8, 5.5 Hz, 1H), 4.38 – 4.24 (m, 2H), 4.14 – 4.03 (m, 1H), 3.68 – 3.61 (m, 1H), 3.60 – 3.53 (m, 1H), 3.42 – 3.33 (m, 4H), 3.22 (t, *J* = 7.0 Hz, 2H), 3.19 (t, *J* = 7.0 Hz, 2H), 3.14 – 3.06 (m, 3H), 2.92 – 2.84 (m, 1H), 2.79 – 2.68 (m, 2H), 2.60 – 2.48 (m, 6H), 2.15 – 2.08 (m, 1H), 1.72 – 1.64 (m, 2H), 1.59 – 1.49 (m, 2H), 1.49 –

1.37 (m, 4H), 1.06 – 0.88 (m, 6H). ^{13}C NMR (151 MHz, Methanol-d_4) δ 173.89, 173.34, 173.04, 170.40, 168.96, 168.28, 167.92, 154.74, 134.55, 124.86, 116.56, 115.24, 105.37, 57.91, 57.80, 50.52, 49.17, 48.91, 42.63, 42.11, 38.92, 37.52, 30.82, 30.74, 30.55, 28.97, 28.31, 26.33, 26.22, 26.13, 22.65, 22.45. HPLC > 95%, t_R = 17.802 min (method D); HRMS (ESI-TOF) m/z: [M + H]$^+$ calcd for $C_{34}H_{48}ClN_6O_9S^+$, 751.2887; found, 751.2890.

N1-(3-(2-chloro-N-((R)-1,1-dioxidotetrahydrothiophen-3-yl)acetamido)-2,2-dimethylpropyl)-N4-(7-((2-(2,6-dioxopiperidin-3-yl)-1,3-dioxoisoindolin-5-yl)amino)heptyl)succinimide (**12**)

Yellow solid (65% yield). ^{1}H NMR (600 MHz, Methanol-d_4) δ 7.57 (d, *J* = 8.4 Hz, 1H), 6.99 (d, *J* = 2.2 Hz, 1H), 6.84 (dd, *J* = 8.4, 2.2 Hz, 1H), 5.06 (dd, *J* = 12.8, 5.5 Hz, 1H), 4.37 – 4.20 (m, 2H), 4.14 – 4.04 (m, 1H), 3.69 – 3.61 (m, 1H), 3.60 – 3.51 (m, 1H), 3.43 – 3.33 (m, 4H), 3.21 (t, *J* = 7.1 Hz, 2H), 3.17 (t, *J* = 7.1 Hz, 2H), 3.14 – 3.05 (m, 3H), 2.94 – 2.83 (m, 1H), 2.80 – 2.68 (m, 2H), 2.61 – 2.48 (m, 6H), 2.18 – 2.09 (m, 1H), 1.71 – 1.63 (m, 2H), 1.56 – 1.49 (m, 2H), 1.48 – 1.42 (m, 2H), 1.42 – 1.32 (m, 4H), 1.05 – 0.90 (m, 6H). ^{13}C NMR (151 MHz, Methanol-d_4) δ 173.88, 173.32, 173.00, 170.40, 168.96, 168.28, 167.91, 154.78, 134.55, 124.84, 116.52, 115.20, 105.32, 57.89, 57.80, 50.52, 49.18, 48.90, 42.72, 42.11, 39.00, 37.52, 30.82, 30.78, 30.57, 28.95, 28.68, 28.35, 26.61, 26.45, 26.13, 22.65, 22.45. HPLC > 95%, t_R = 18.779 min (method D); HRMS (ESI-TOF) m/z: [M + H]$^+$ calcd for $C_{35}H_{50}ClN_6O_9S^+$, 765.3043; found, 765.3042.

N1-(3-(2-chloro-N-((R)-1,1-dioxidotetrahydrothiophen-3-yl)acetamido)-2,2-dimethylpropyl)-N4-(8-((2-(2,6-dioxopiperidin-3-yl)-1,3-dioxoisoindolin-5-yl)amino)octyl)succinamide (**13**)

Yellow solid (74% yield). ^{1}H NMR (600 MHz, Methanol-d_4) δ 7.57 (d, *J* = 8.4 Hz, 1H), 6.99 (d, *J* = 2.1 Hz, 1H), 6.85 (dd, *J* = 8.5, 2.1 Hz, 1H), 5.06 (dd, *J* = 12.8, 5.5 Hz, 1H), 4.35 – 4.26 (m, 2H), 4.14 – 4.03 (m, 1H), 3.68 – 3.62 (m, 1H), 3.60 – 3.53 (m, 1H), 3.42 – 3.33 (m, 4H), 3.22 (t, *J* = 7.1 Hz, 2H), 3.17 (t, *J* = 7.1 Hz, 2H), 3.14 – 3.05 (m, 3H), 2.92 – 2.84 (m, 1H), 2.80 – 2.67 (m, 2H), 2.61 – 2.49 (m, 6H), 2.15 – 2.08 (m, 1H), 1.72 – 1.63 (m, 2H), 1.56 – 1.26 (m, 12H), 1.04 – 0.92 (m, 6H). ^{13}C NMR (151 MHz, Methanol-d_4) δ 173.90, 173.34, 173.00, 170.41, 168.96, 168.28, 167.92, 154.75, 134.55, 124.84, 116.54, 115.24, 105.33, 57.88, 57.80, 50.51, 49.16, 48.90, 42.77, 42.14, 39.05, 37.54, 30.83, 30.77, 30.55, 29.01, 28.95, 28.90, 28.39, 26.63, 26.47, 26.14, 22.65, 22.46. HPLC > 95%, t_R = 19.877 min (method D); HRMS (ESI-TOF) m/z: [M + H]$^+$ calcd for $C_{36}H_{52}ClN_6O_9S^+$, 779.3200; found, 779.3203.

N^1-(3-(2-chloro-N-((R)-1,1-dioxidotetrahydrothiophen-3-yl)acetamido)-2,2-dimethylpropyl)-N^4-(12-((2-(1-methyl-2,6-dioxopiperidin-3-yl)-1,3-dioxoisoindolin-5-yl)amino)dodecyl)succinimide (**P1D-34N1**)

Yellow solid (81% yield). ^{1}H NMR (500 MHz, Methanol-d_4) δ 7.55 (d, J = 8.4 Hz, 1H), 6.97 (d, J = 2.2 Hz, 1H), 6.83 (dd, J = 8.4, 2.2 Hz, 1H), 5.06 (dd, J = 12.9, 5.4 Hz, 1H), 4.35 – 4.21 (m, 2H), 4.12 – 4.02 (m, 1H), 3.66 – 3.50 (m, 2H), 3.40 – 3.33 (m, 2H), 3.20 (t, J = 7.1 Hz, 2H), 3.16 – 3.12 (m, 5H), 3.11 – 3.03 (m, 3H), 2.90 – 2.83 (m, 2H), 2.72 – 2.61 (m, 1H), 2.57 – 2.47 (m, 6H), 2.13 – 2.04 (m, 1H), 1.70 – 1.61 (m, 2H), 1.52 – 1.39 (m, 4H), 1.39 – 1.21 (m, 16H), 0.99 (s, 6H). ^{13}C NMR (126 MHz, Methanol-d_4) δ 173.89, 172.97, 172.32, 170.17, 168.96, 168.31, 167.95, 154.84, 134.56, 124.84, 116.50, 115.17, 105.31, 57.89, 57.82, 50.52, 49.56, 49.17, 42.77, 42.07, 39.09, 37.50, 31.10, 30.81, 30.59, 29.22, 29.04, 28.99, 28.44, 26.69, 26.57, 26.12, 25.92, 22.66, 21.70. HPLC > 95%, t_R = 15.210 min (method B); HRMS (ESI-TOF) m/z: [M + H]$^+$ calcd for $C_{41}H_{62}ClN_6O_9S^+$, 849.3982; found, 849.3983.

N^1-(3-(N-((R)-1,1-dioxidotetrahydrothiophen-3-yl)acetamido)-2,2-dimethylpropyl)-N^4-(12-((2-(2,6-dioxopiperidin-3-yl)-1,3-dioxoisoindolin-5-yl)amino)dodecyl)succinimide (**P1D-34N2**)

Yellow solid (90% yield). ^{1}H NMR (500 MHz, Methanol-d_4) δ 7.55 (d, J = 8.4 Hz, 1H), 6.97 (d, J = 2.2 Hz, 1H), 6.82 (dd, J = 8.4, 2.2 Hz, 1H), 5.03 (dd, J = 12.6, 5.5 Hz, 1H), 4.06 – 3.95 (m, 1H), 3.72 – 3.63 (m, 1H), 3.58 – 3.46 (m, 1H), 3.41 – 3.34 (m, 1H), 3.29 – 3.24 (m, 1H), 3.23 – 3.17 (m, 2H), 3.17 – 3.09 (m, 4H), 3.08 – 3.01 (m, 1H), 2.90 – 2.80 (m, 1H), 2.78 – 2.64 (m, 2H), 2.61 – 2.54 (m, 1H), 2.53 – 2.45 (m, 5H), 2.11 (s, 3H), 2.09 – 2.05 (m, 1H), 1.64 (q, J = 7.2 Hz, 2H), 1.45 (dt, J = 17.8, 7.2 Hz, 4H), 1.37 – 1.24 (m, 16H), 0.98 (s, 6H). ^{13}C NMR (126 MHz, Methanol-d_4) δ 173.82, 173.67, 173.29, 172.99, 170.36, 168.28, 167.92, 154.83, 134.56, 124.82, 116.52, 115.15, 105.30, 58.74, 57.26, 50.57, 49.44, 48.90, 42.75, 39.08, 37.63, 30.81, 30.66, 29.21, 29.02, 28.43, 26.68, 26.57, 26.40, 22.76, 22.46, 22.15. HPLC > 95%, t_R = 18.348 min (method A); HRMS (ESI-TOF) m/z: [M + H]$^+$ calcd for $C_{40}H_{61}N_6O_9S^+$, 801.4215; found, 801.4212.

Synthesis of *2-chloro-N-(1,1-dioxidotetrahydrothiophen-3-yl)-N-neopentylacetamide* (**Sulfopin**)

3-aminosulfolane hydrochloride (200 mg, 1.166 mmol) was added to a solution of TEA (146 µL) in dry DMF (3 ml) and stirred for 1 h at RT. Afterwards, pivaldehyde (140 µL, 1.282 mmol)

and one drop of HOAC (13 μL, 0.233 mmol, 0.2 eq) was added to the reaction mixture and stirred at RT for 1 h. Then STAB (520 mg, 2.446 mmol, 2.1 eq) was added at once to the mixture and stirred overnight at RT. After evaporation of the solvent, the residue was dissolved with saturated $NaHCO_3$ (5 ml) and was extracted with EA (2 × 5 ml). The organic layers were combined, dried over Na_2SO_4 and filtered. Evaporation of the solvent yielded **21** as white solid, which was used without purification in the next step.

21 (100 mg, 0.452 mmol) was dissolved in DCM (3 ml) at 0 ℃, chloroacetyl chloride (43 μL, 0.543 mmol, 1.2 eq) and TEA (75 μL, 0.543 mmol, 1.2 eq) were added dropwise at 0 ℃ and stirred for 30 min. Afterwards the reaction mixture was allowed to reach RT and stirred for 2 h. The reaction was quenched at 0 ℃ by addition of water.The residue was purified by HPLC (mobile phase: CH_3CN containing 0.1% TFA and H_2O containing 0.1% TFA) to afford the product **Sulfopin**.

White solid (87% yield).^{1}H NMR (600 MHz, Chloroform-*d*) δ 4.17 – 4.01 (m, 2H), 3.99 – 3.87 (m, 1H), 3.77 – 3.66 (m, 2H), 3.27 (d, *J* = 15.5 Hz, 1H), 3.17 – 3.07 (m, 2H), 3.06 – 2.99 (m, 1H), 2.58 – 2.47 (m, 2H), 1.01 (s, 9H). ^{13}C NMR (201 MHz, Chloroform-*d*) δ 167.87, 62.51, 57.70, 50.39, 49.11, 42.25, 33.76, 28.19, 26.73 (spectral data obtained were identical with those reported in literature[1]). HPLC > 95%, t_R = 20.676 min (method C); HRMS (ESI-TOF) m/z: [M + H]$^+$ calcd for $C_{11}H_{21}ClNO_3S^+$, 282.0925; found, 282.0926.

3.2. ^{1}H and ^{13}C of compound 1 – 13, P1D-34N1 ,P1D-34N2 and Sulfopin.

^{1}H NMR and ^{13}C NMR of compound **1**

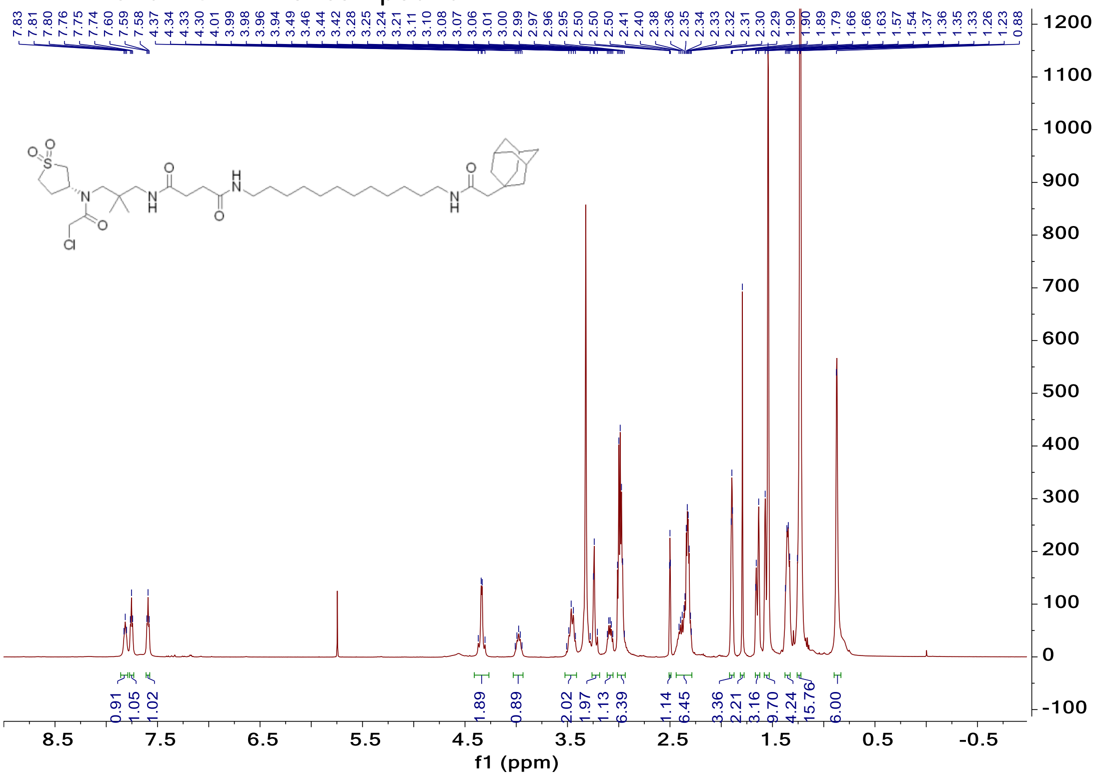

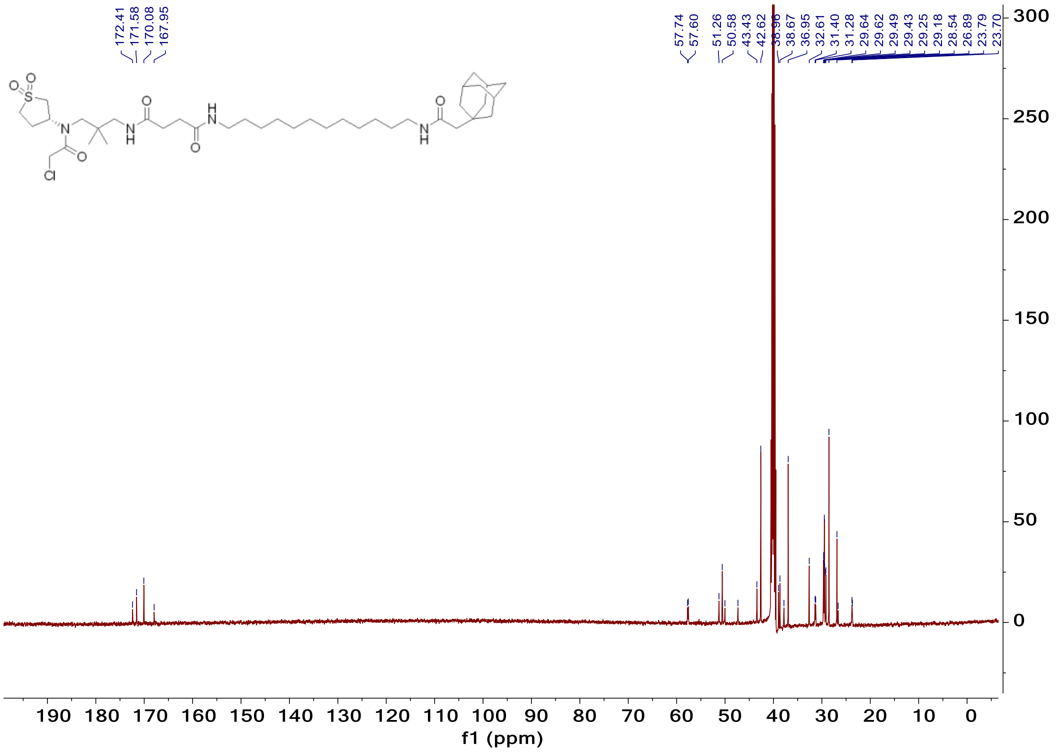

^{1}H NMR and ^{13}C NMR of compound **2**

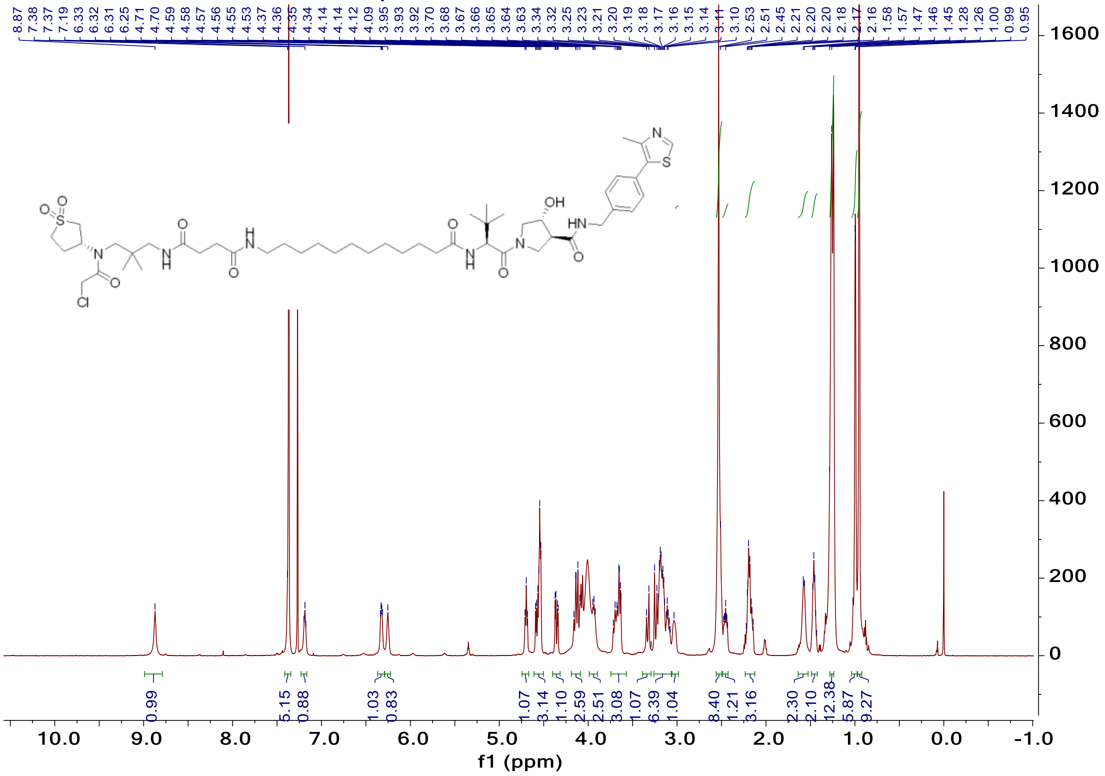

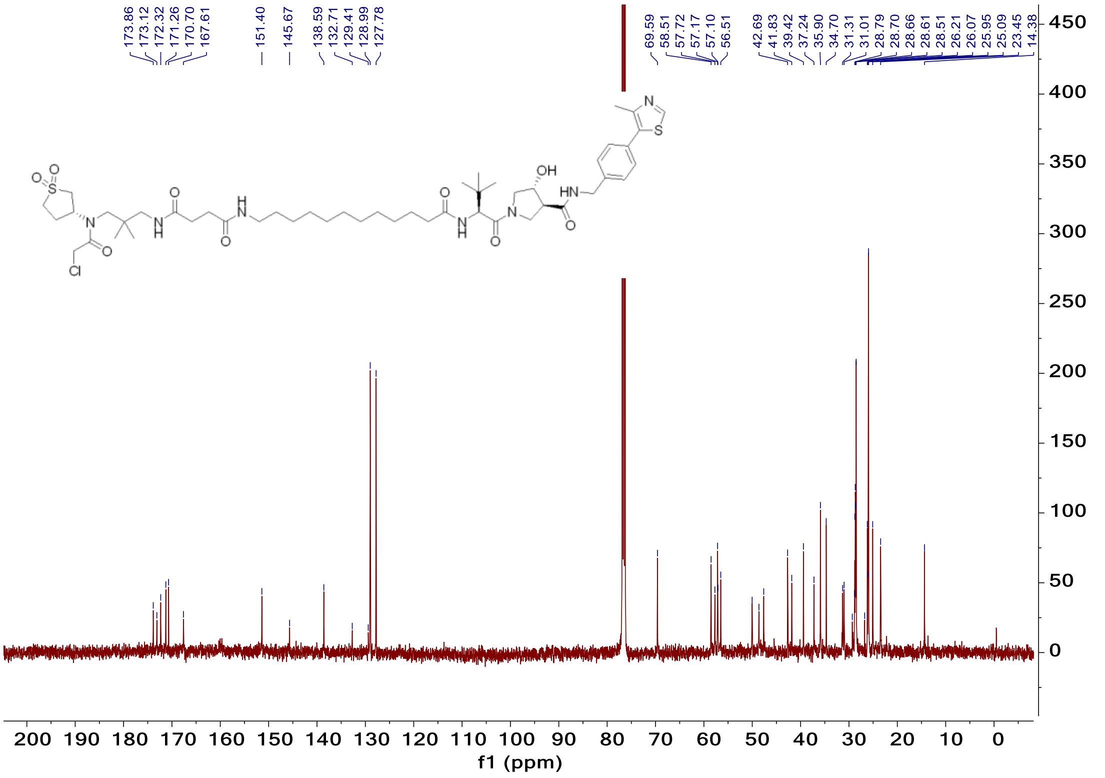

^{1}H NMR and ^{13}C NMR of compound **3** (**P1D-34**)

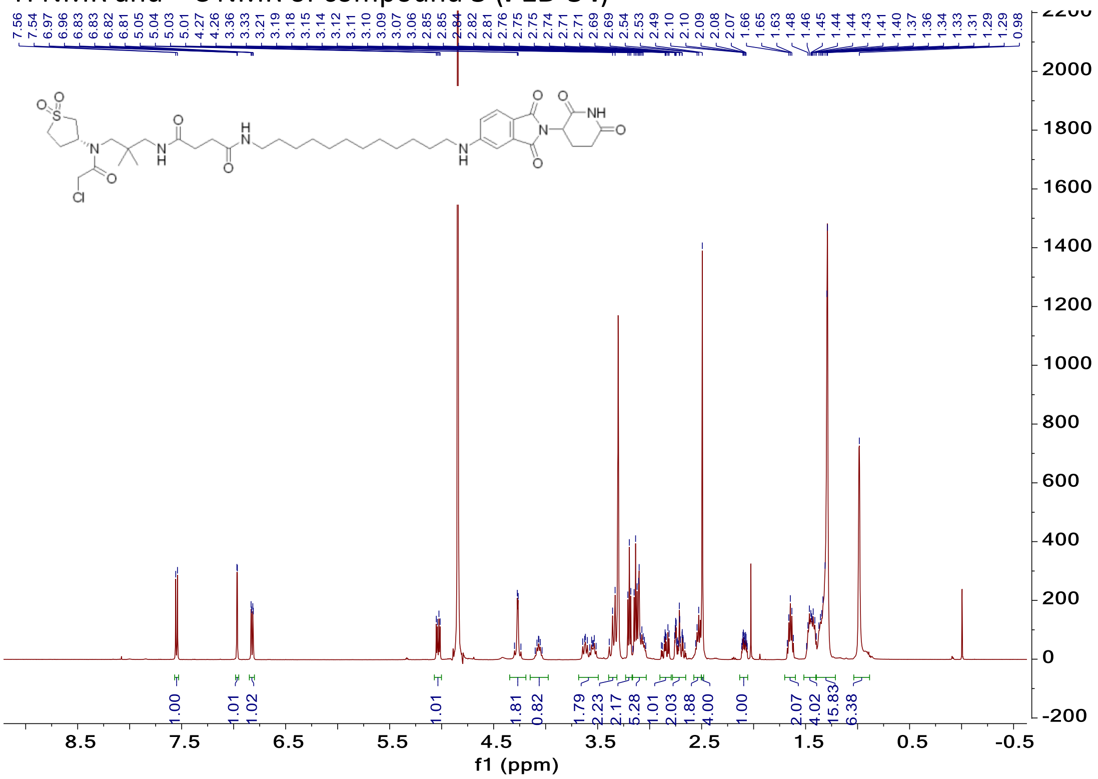

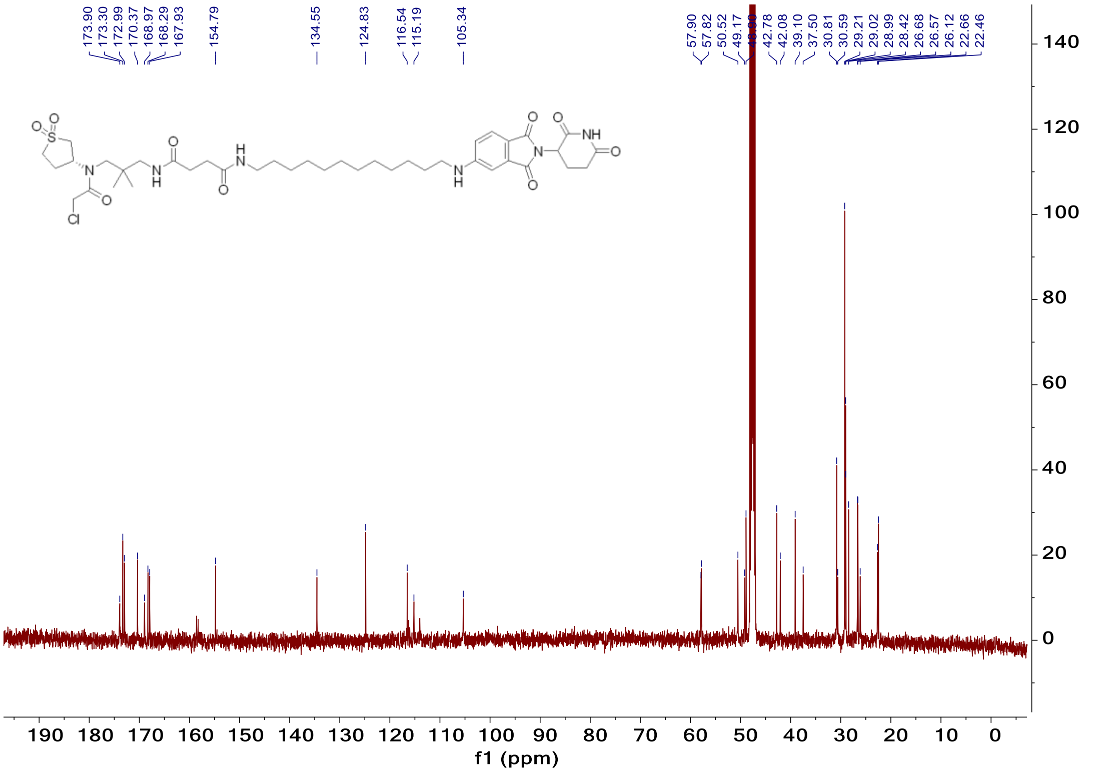

^{1}H NMR and ^{13}C NMR of compound **4**

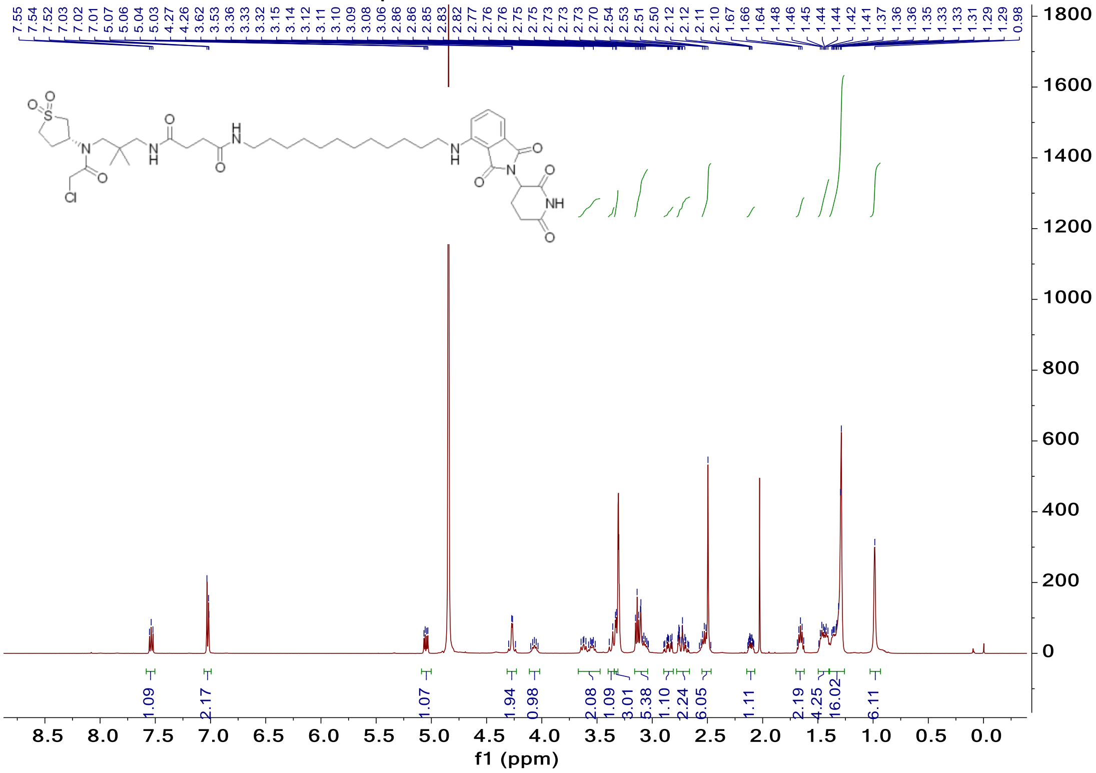

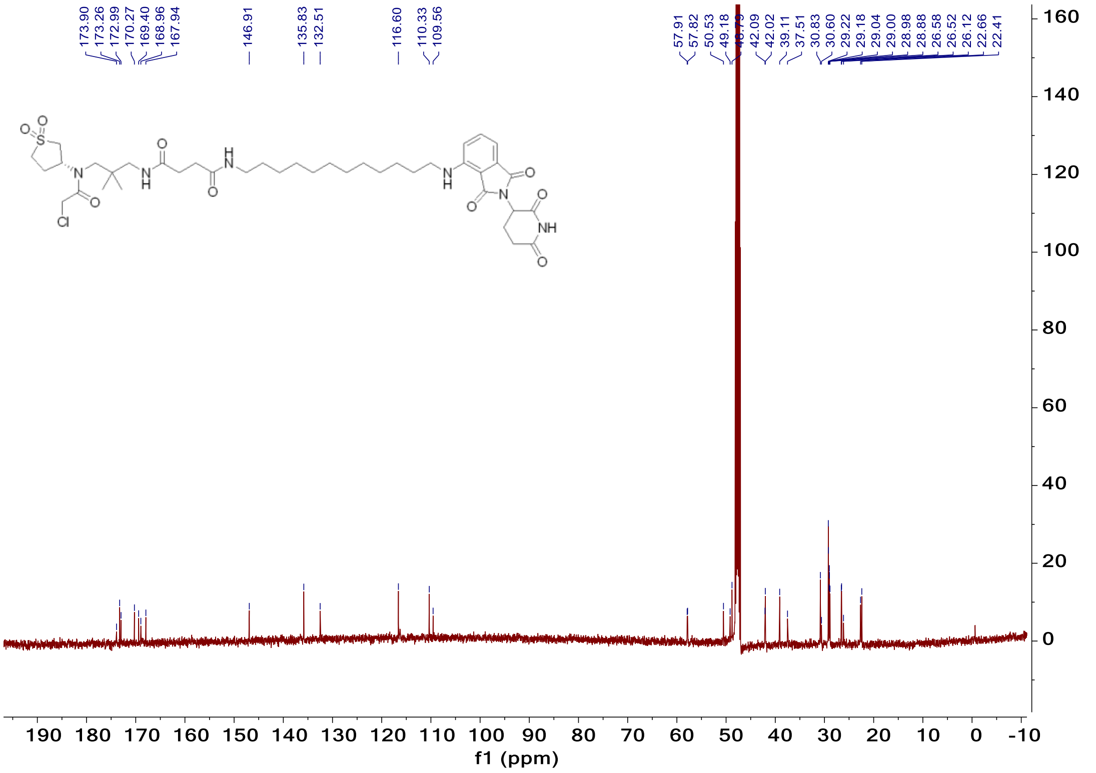

^{1}H NMR and ^{13}C NMR of compound **5**

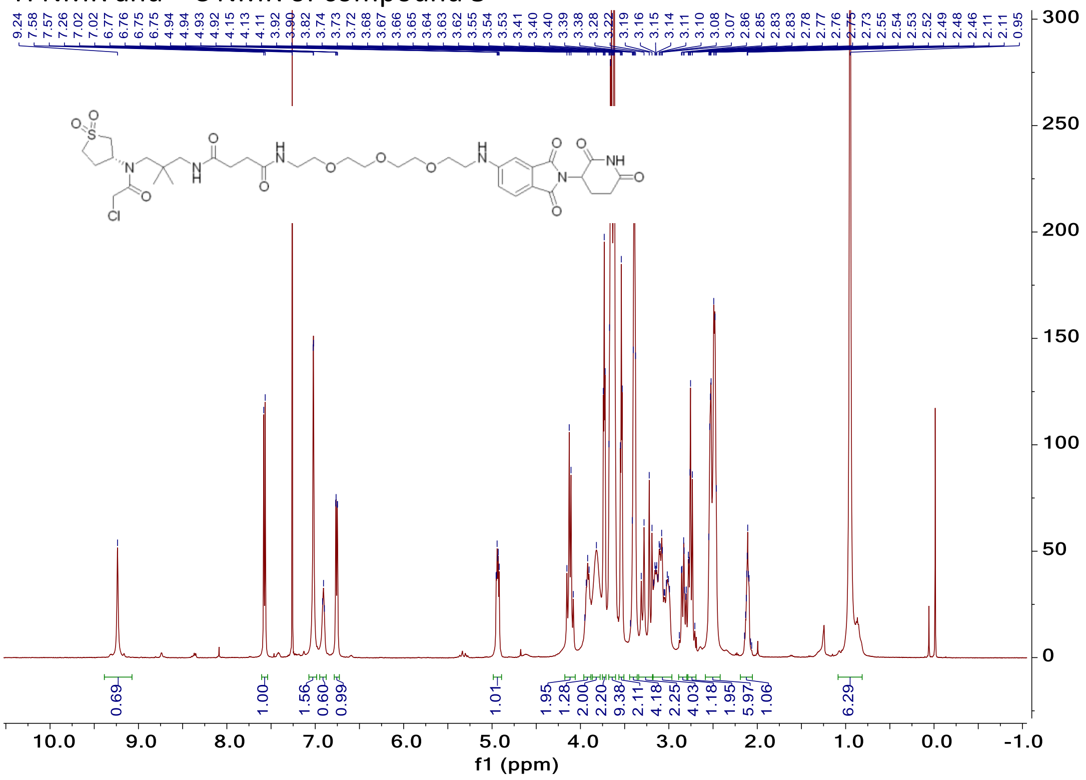

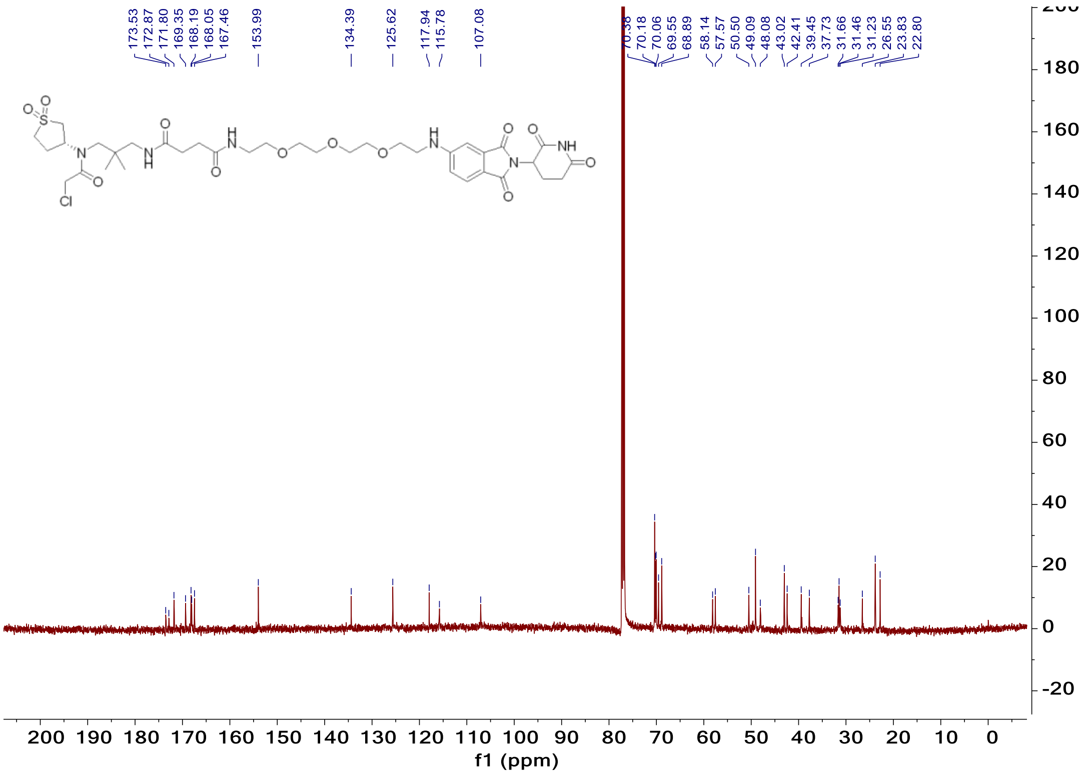

^{1}H NMR and ^{13}C NMR of compound **6**

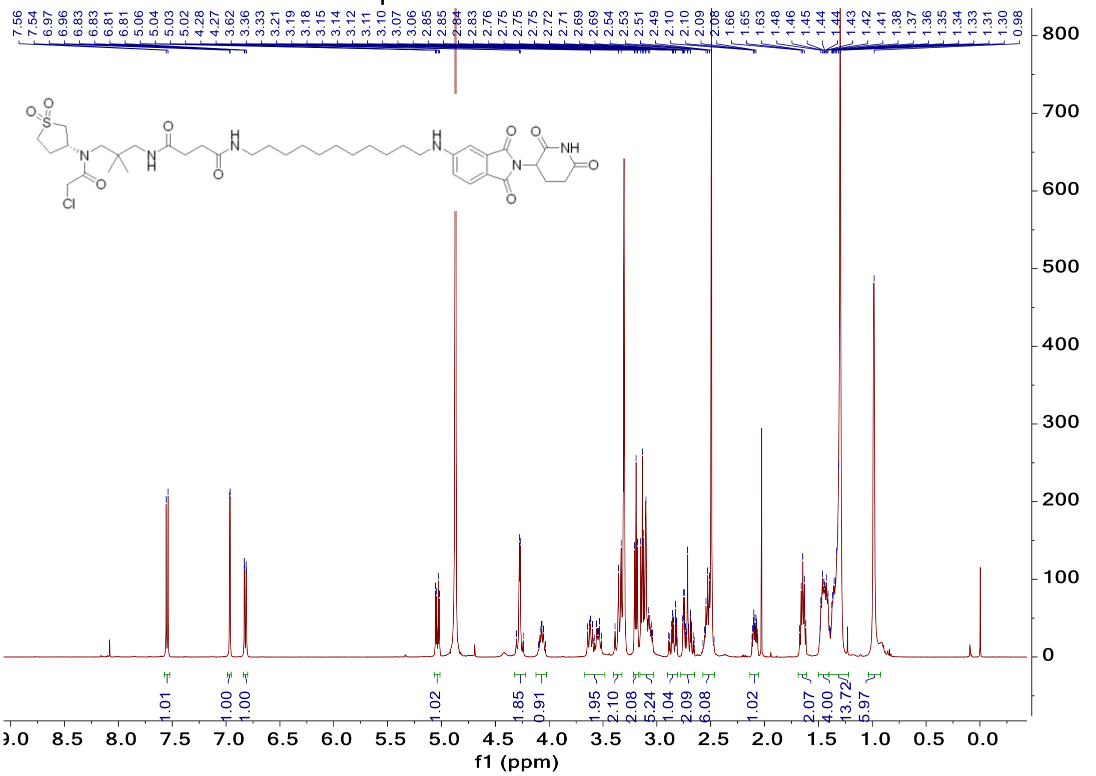

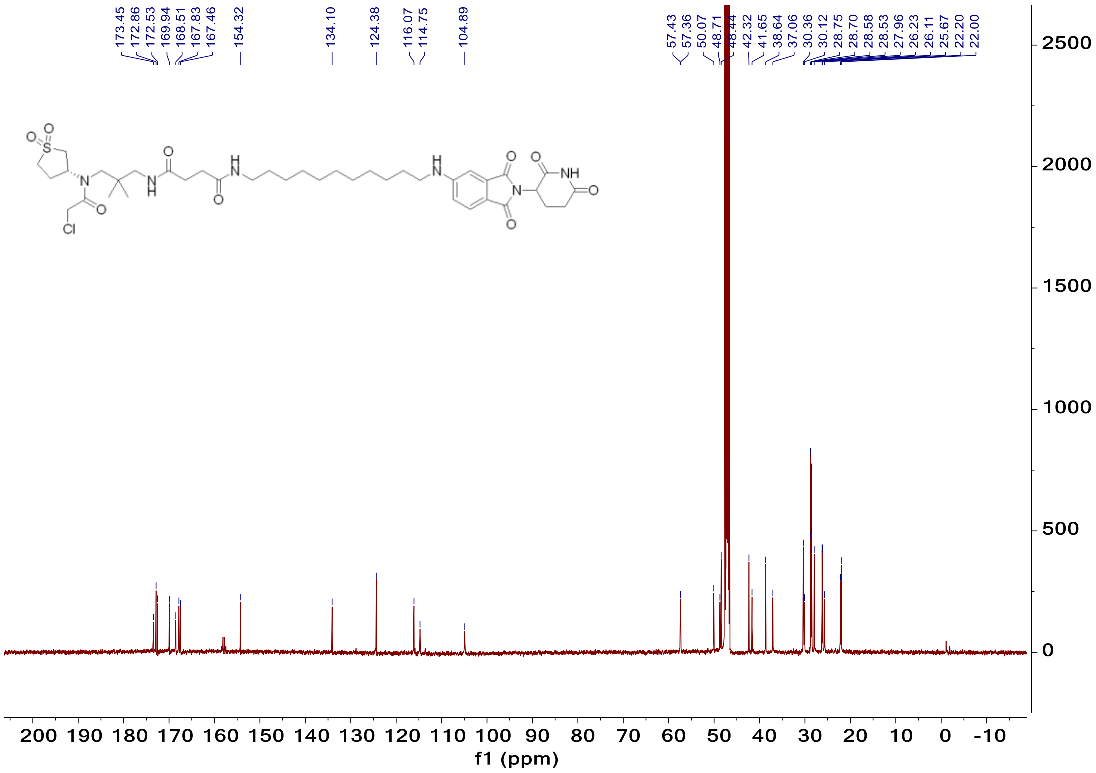

^{1}H NMR and ^{13}C NMR of compound **7**

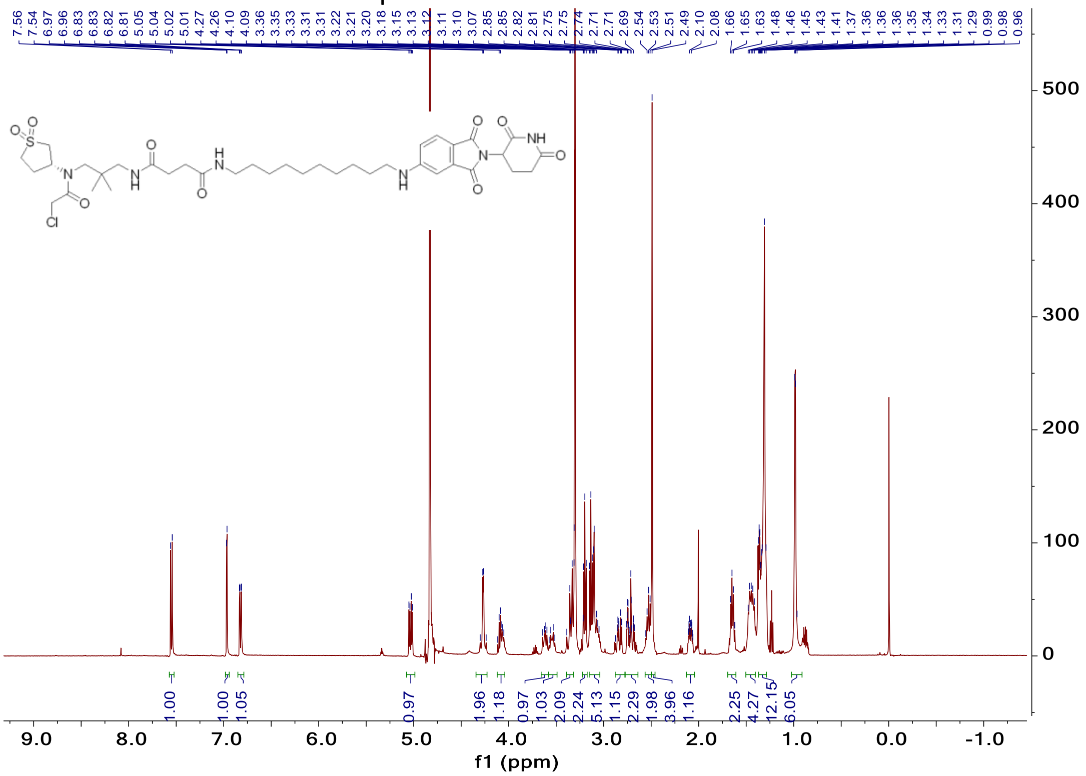

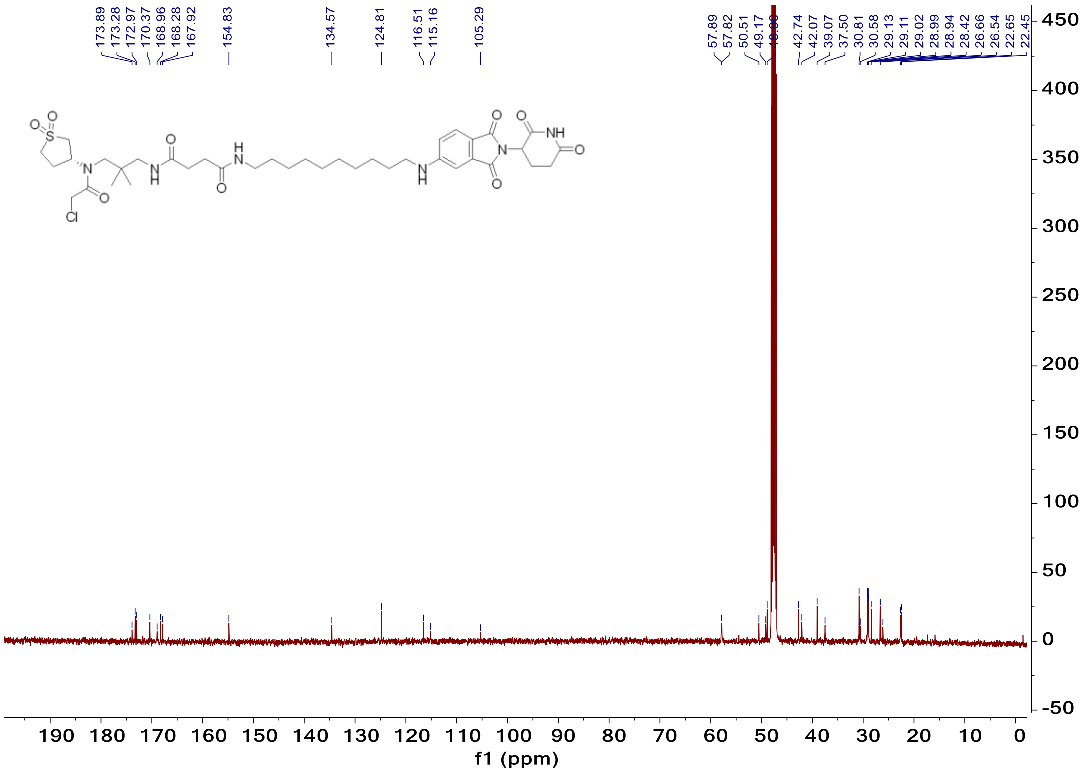

^{1}H NMR and ^{13}C NMR of compound **8**

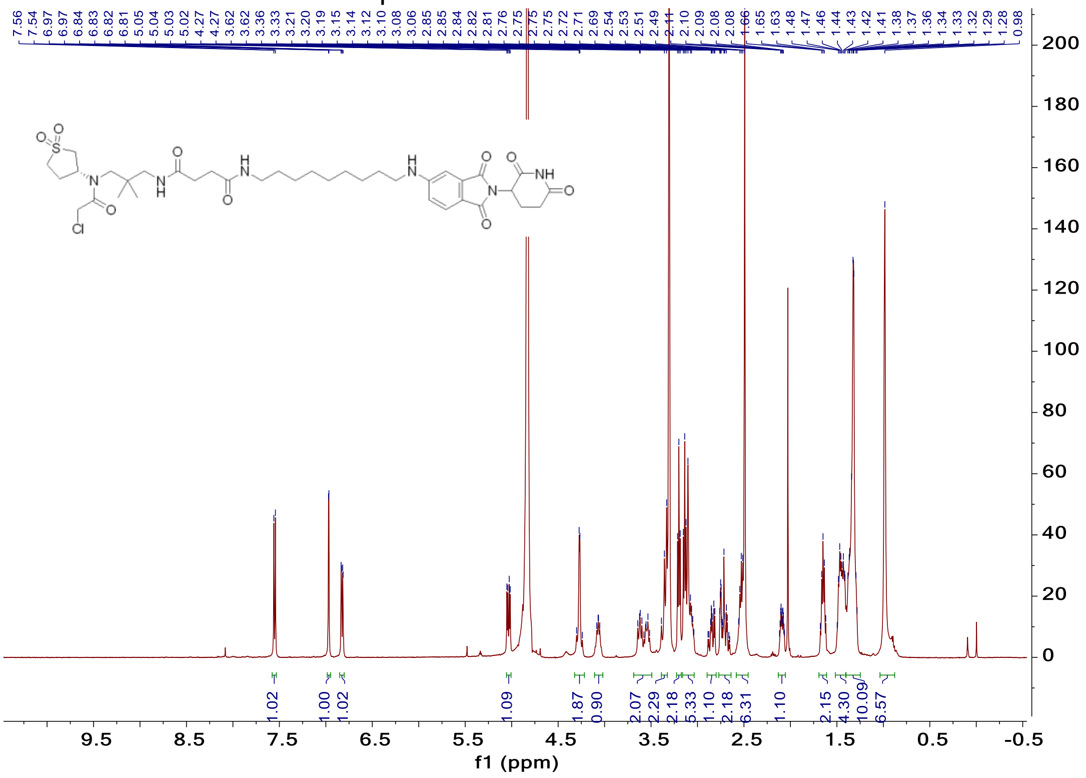

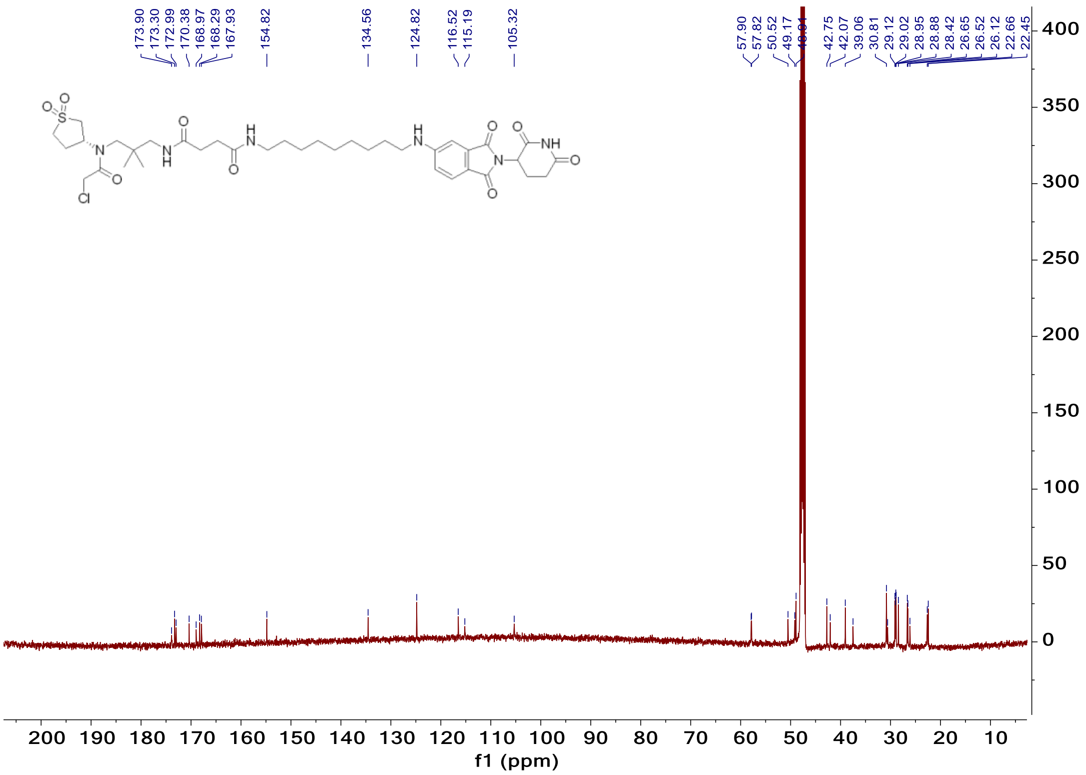

^{1}H NMR and ^{13}C NMR of compound **9**

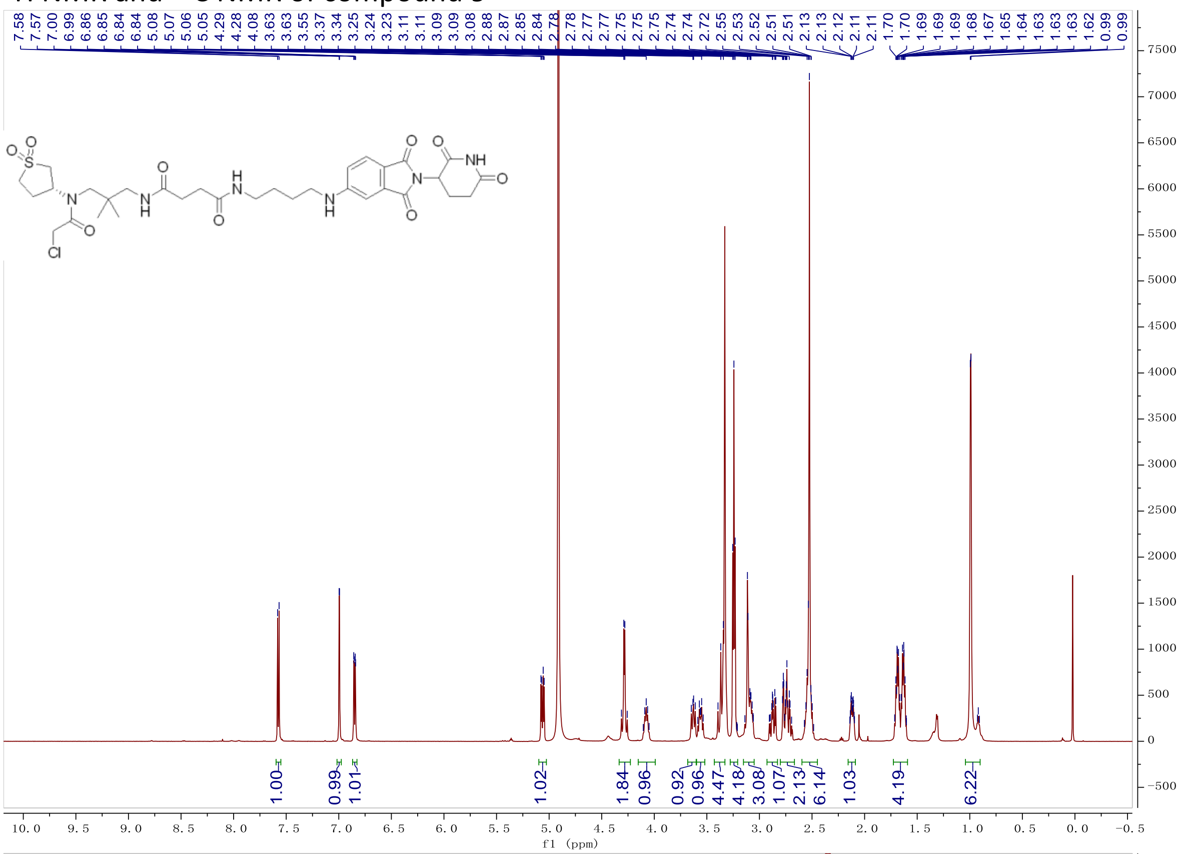

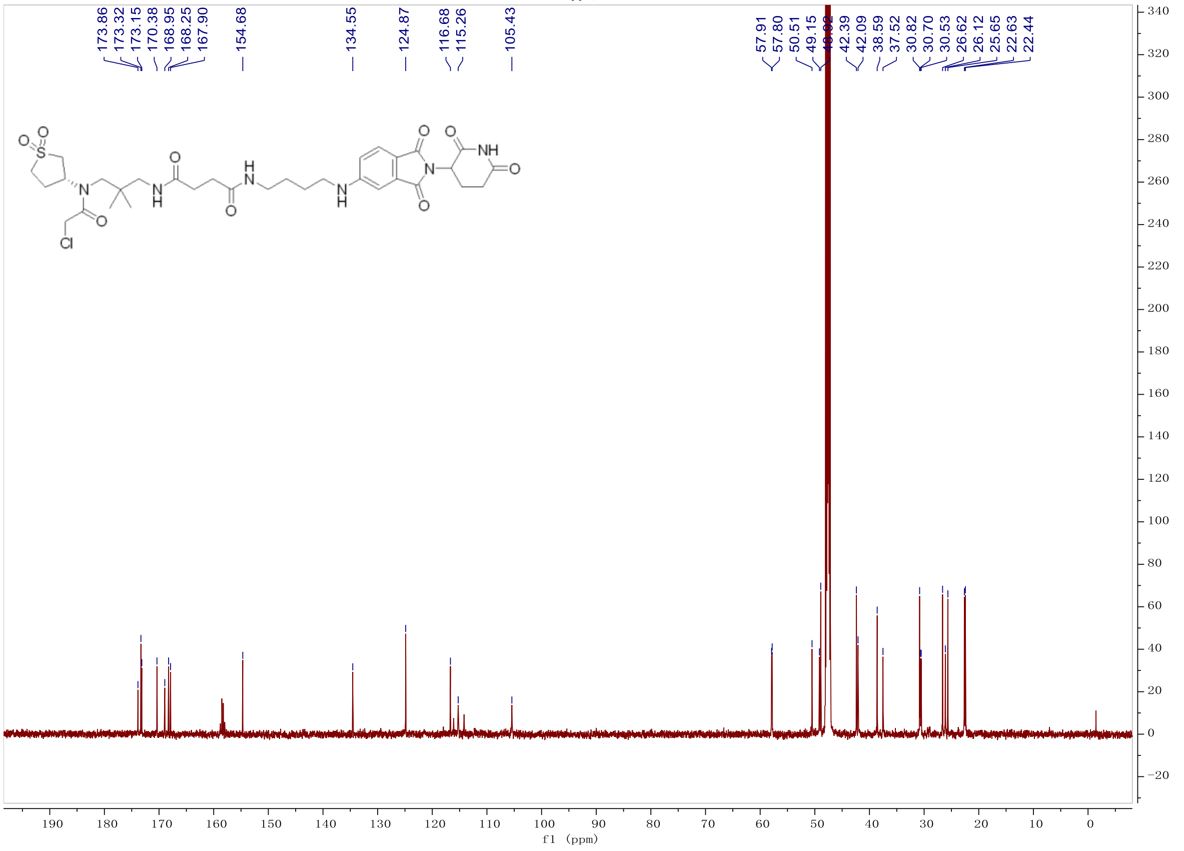

^{1}H NMR and ^{13}C NMR of compound **10**

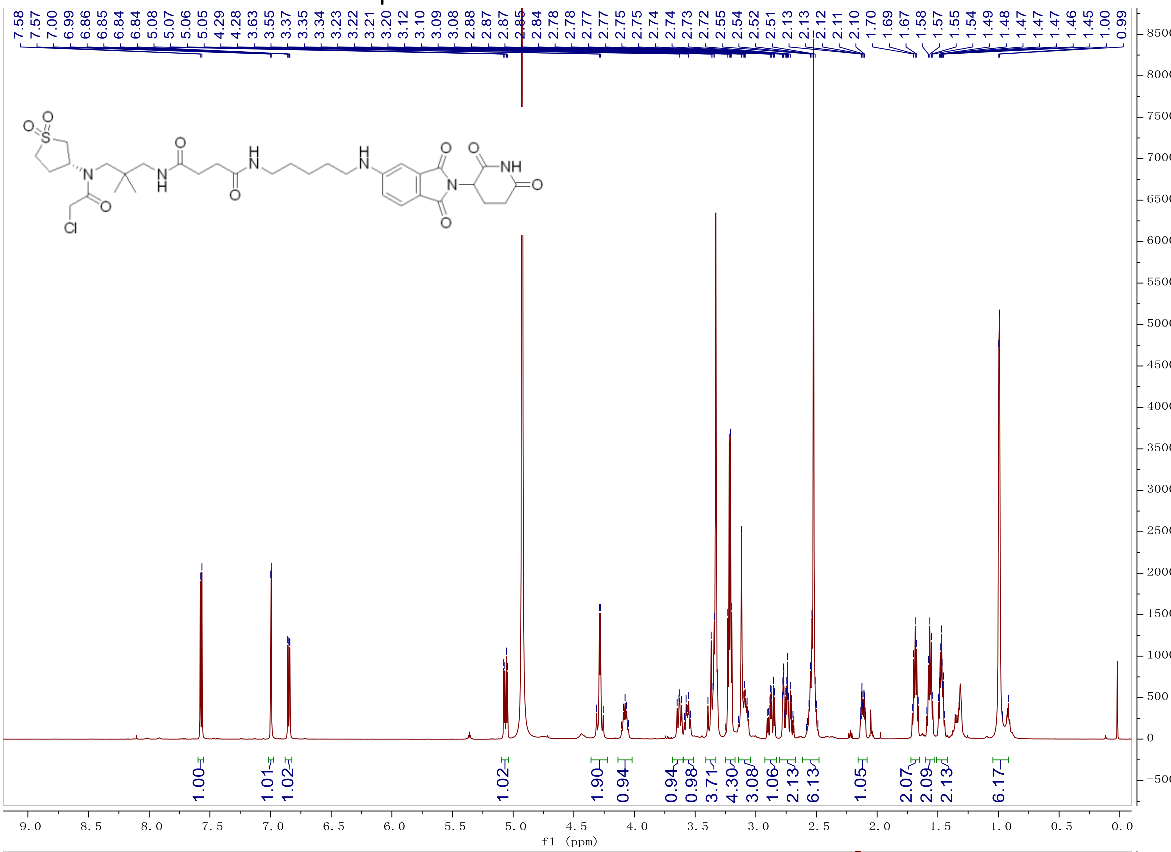

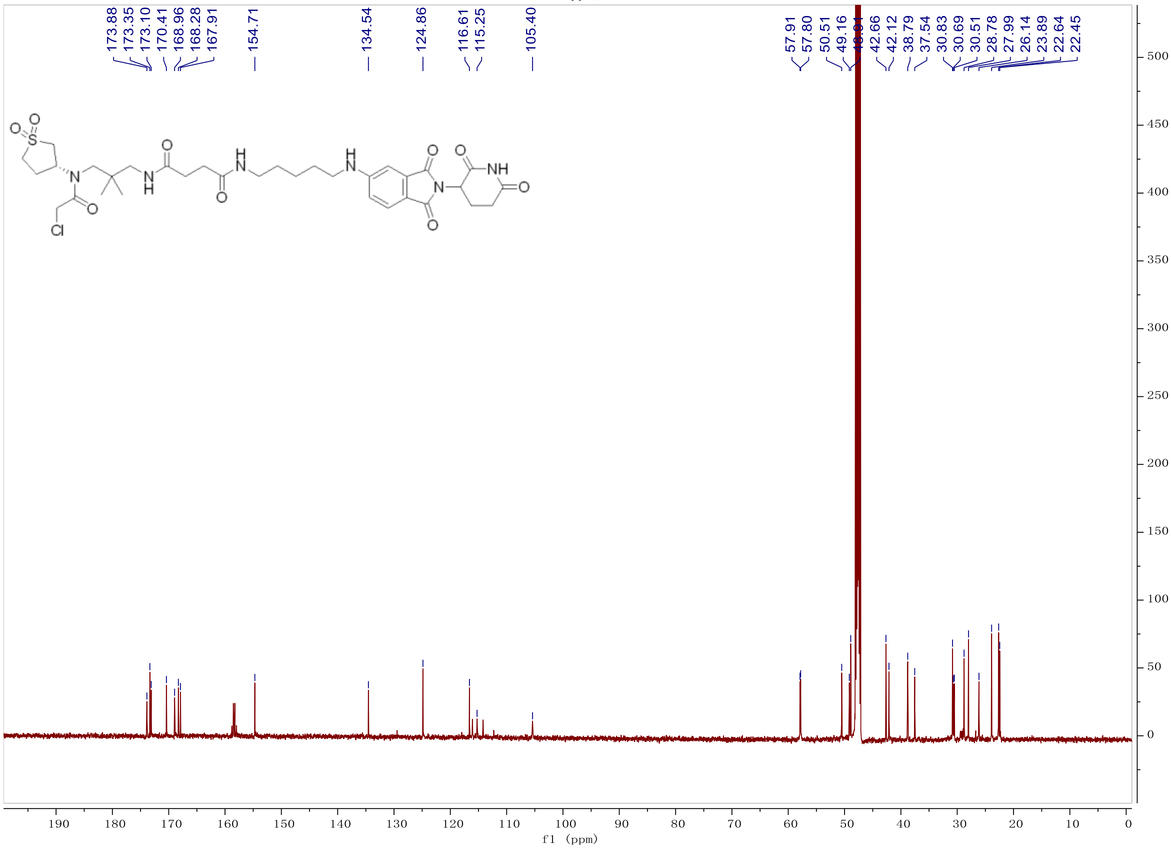

^{1}H NMR and ^{13}C NMR of compound **11**

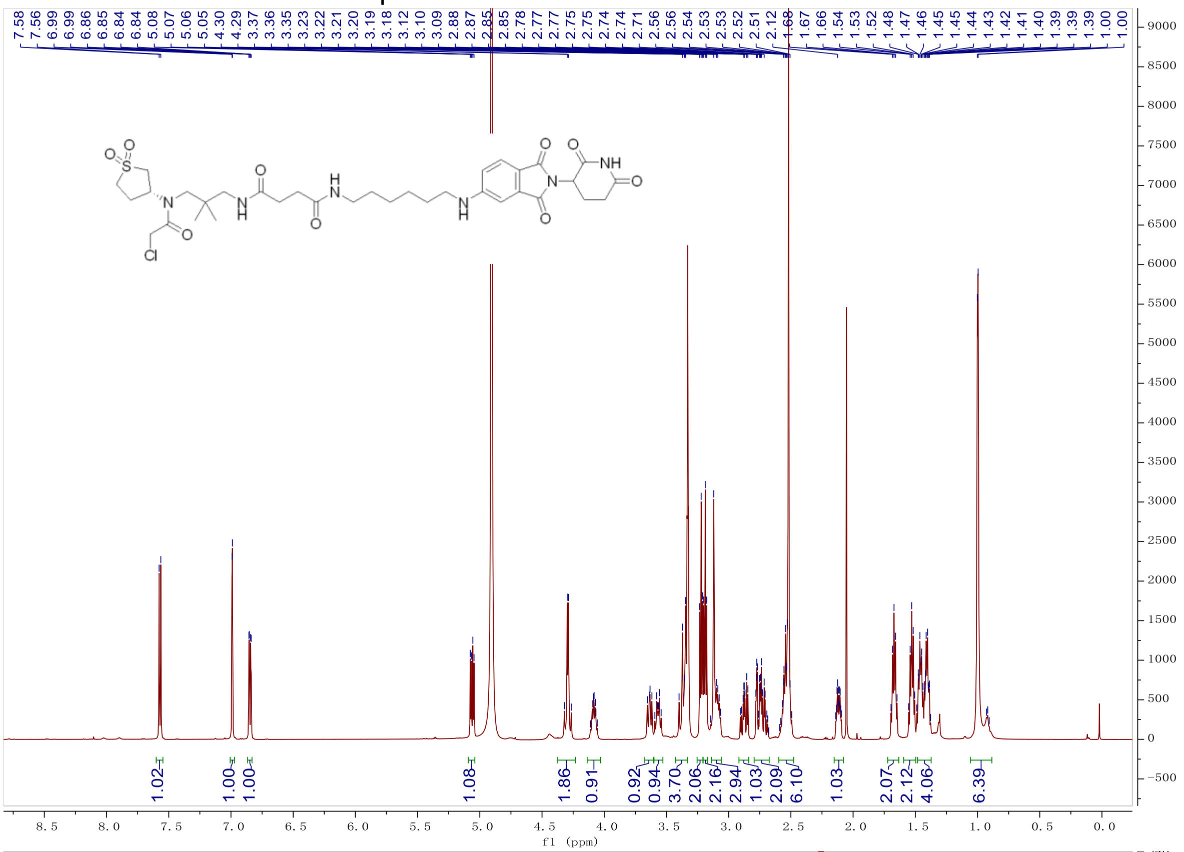

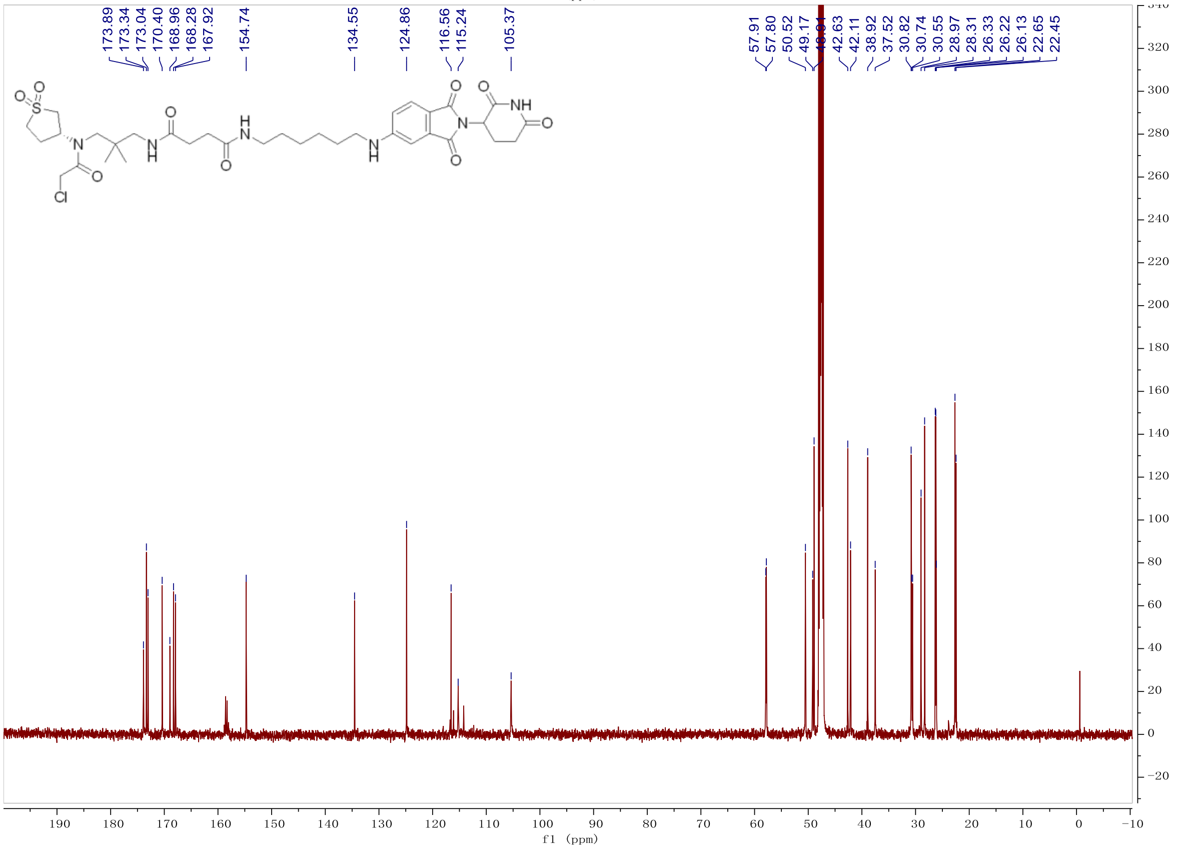

^{1}H NMR and ^{13}C NMR of compound **12**

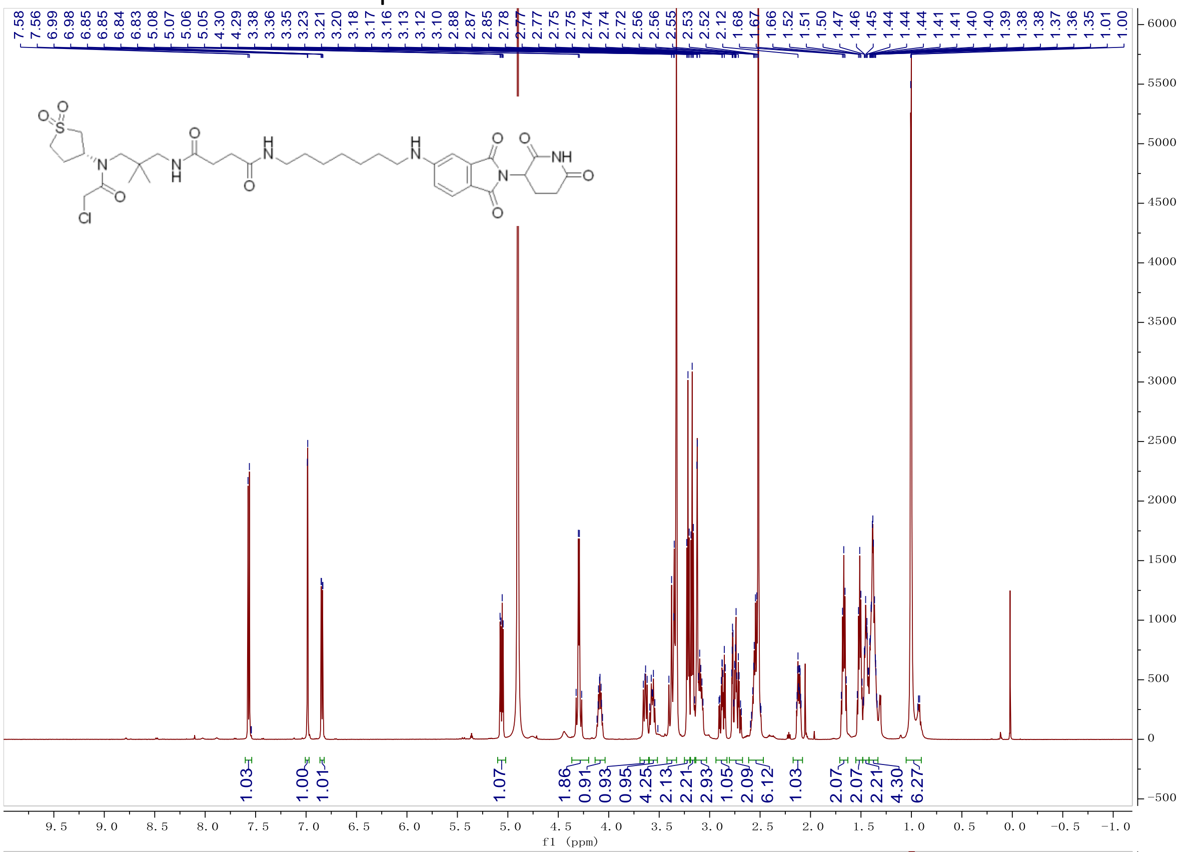

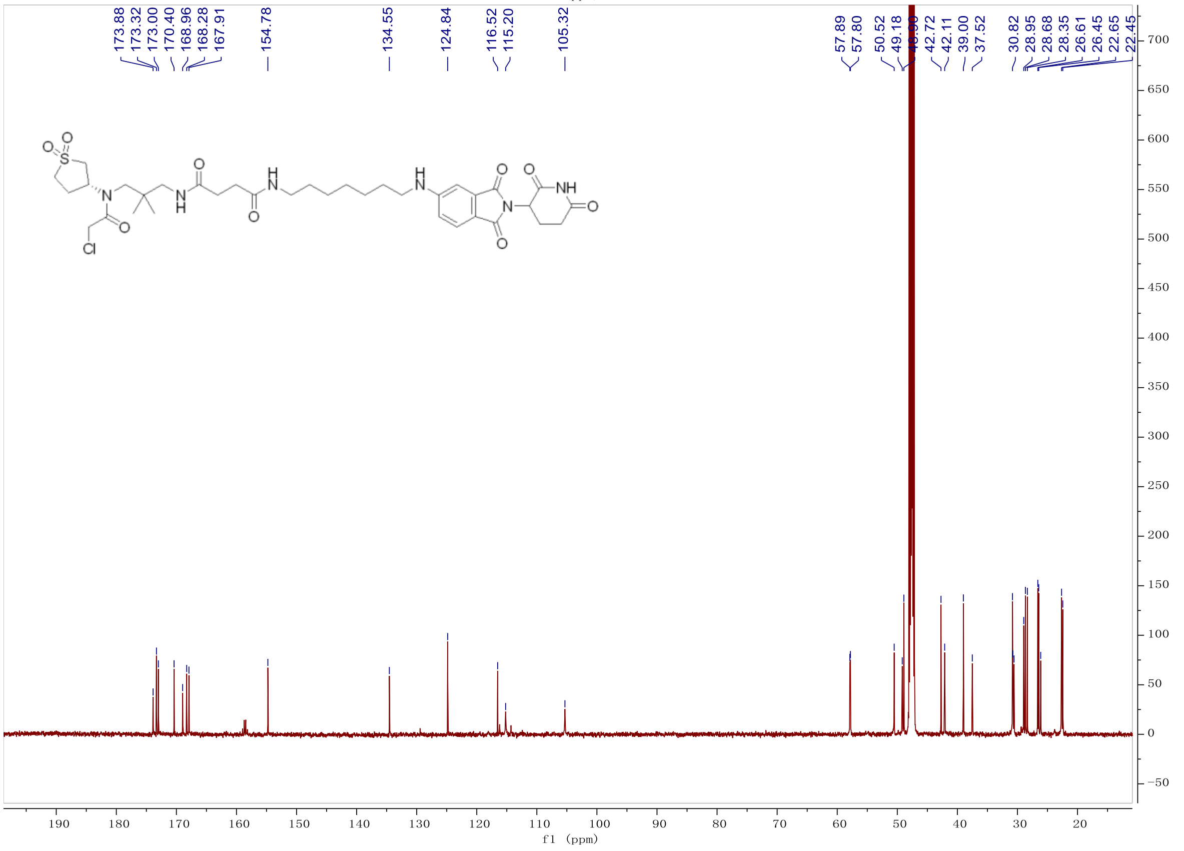

^{1}H NMR and ^{13}C NMR of compound **13**

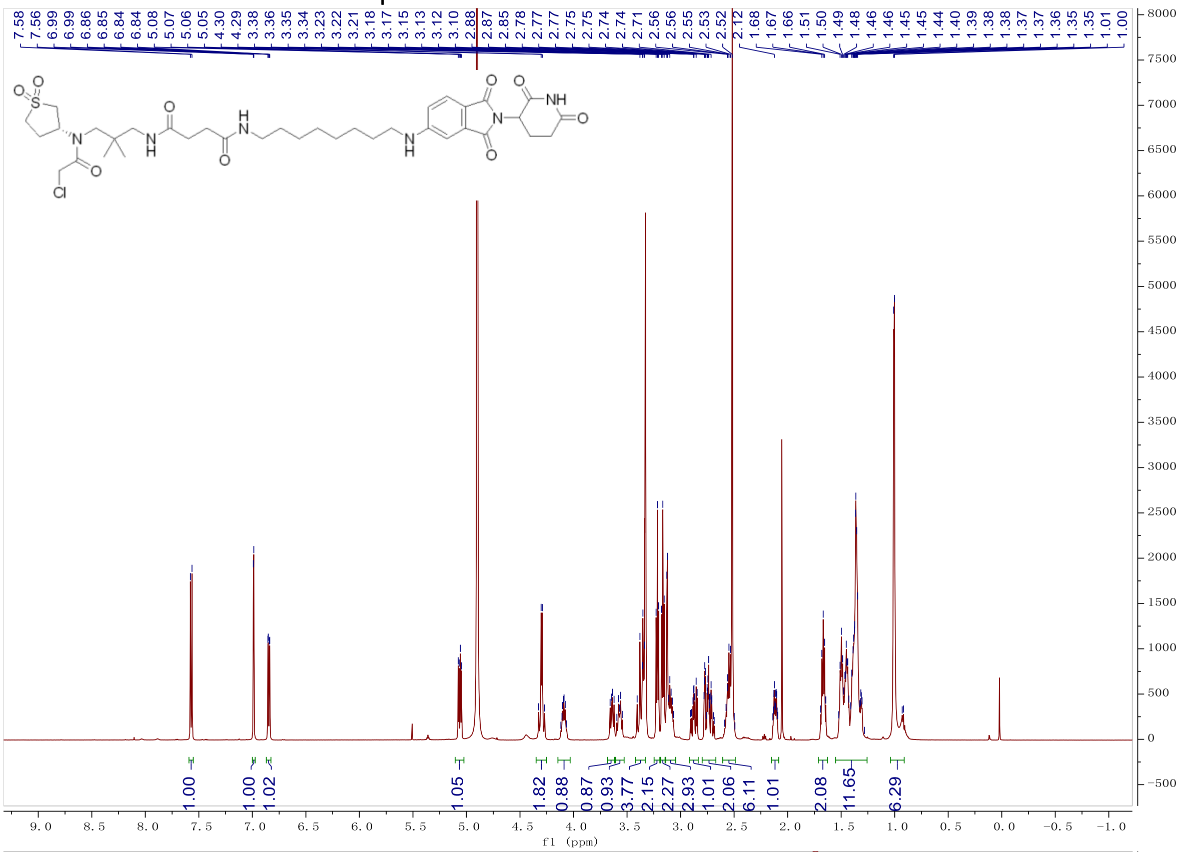

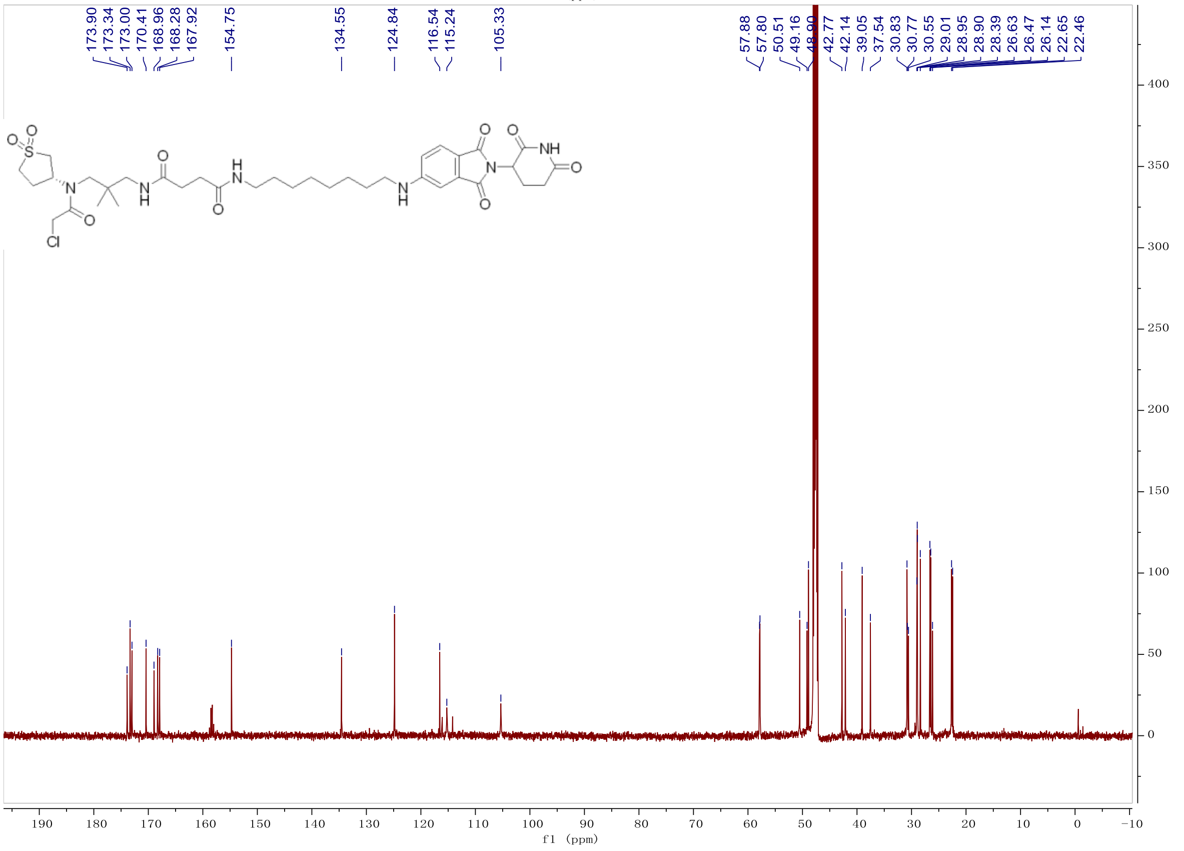

^{1}H NMR and ^{13}C NMR of compound **P1D-34N1**

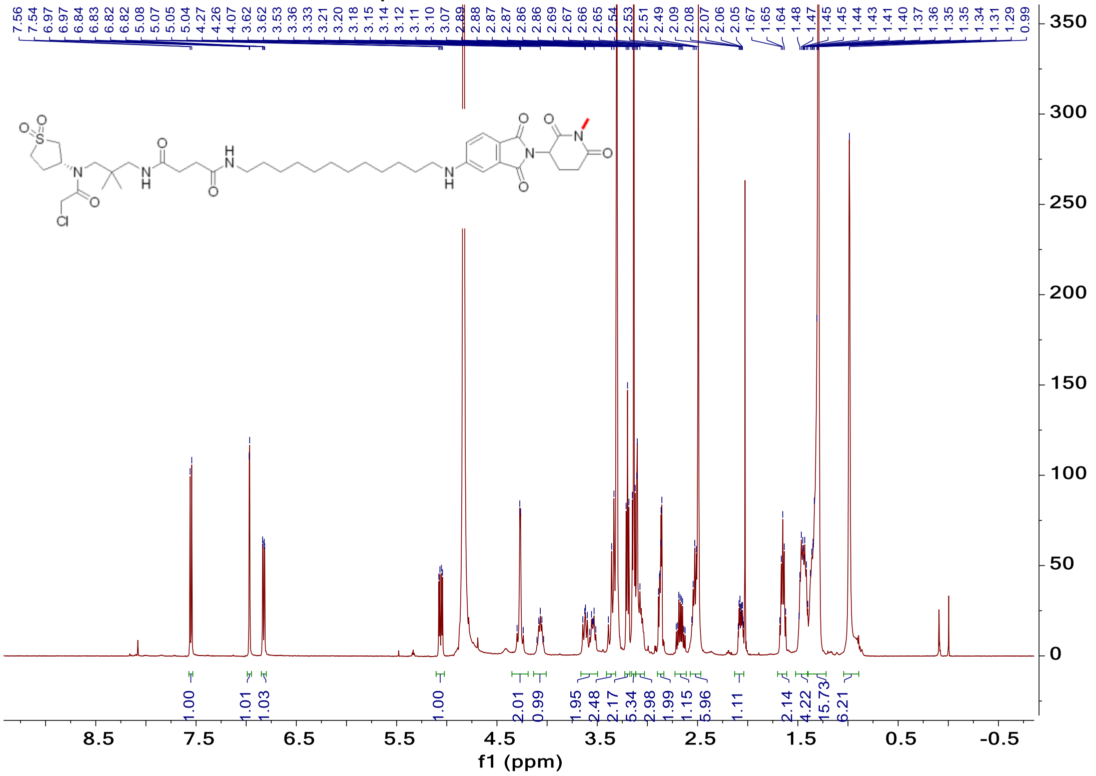

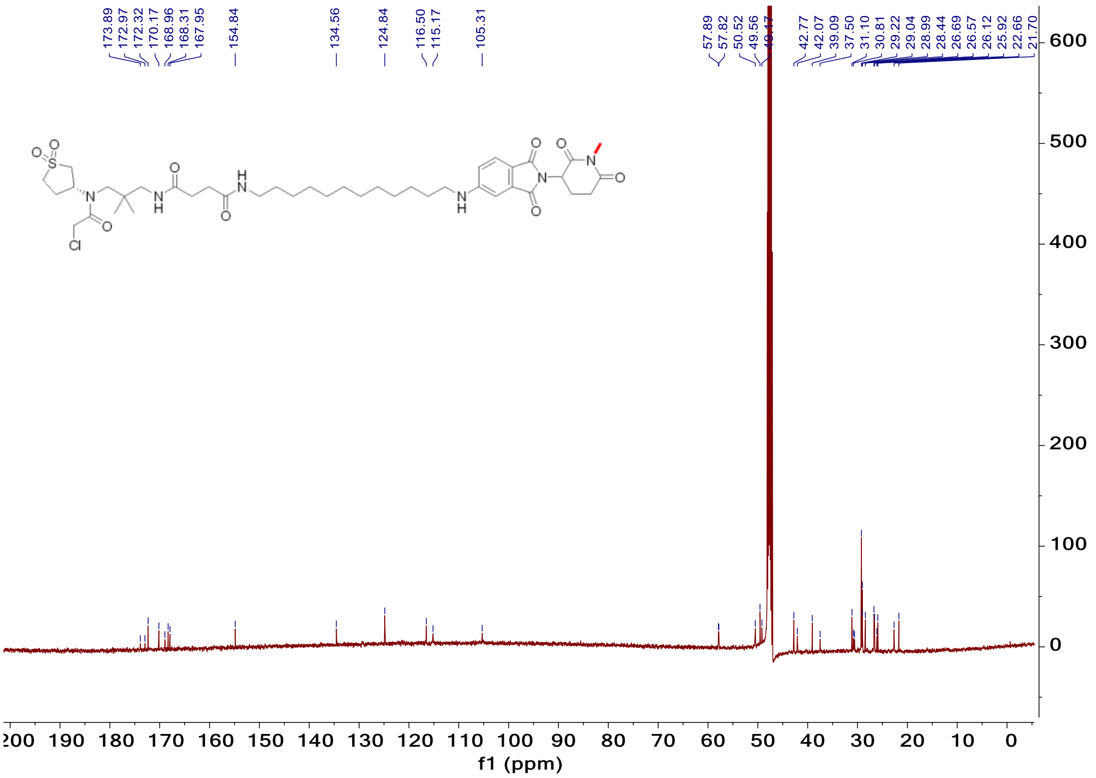

^{1}H NMR and ^{13}C NMR of compound **P1D-34N2**

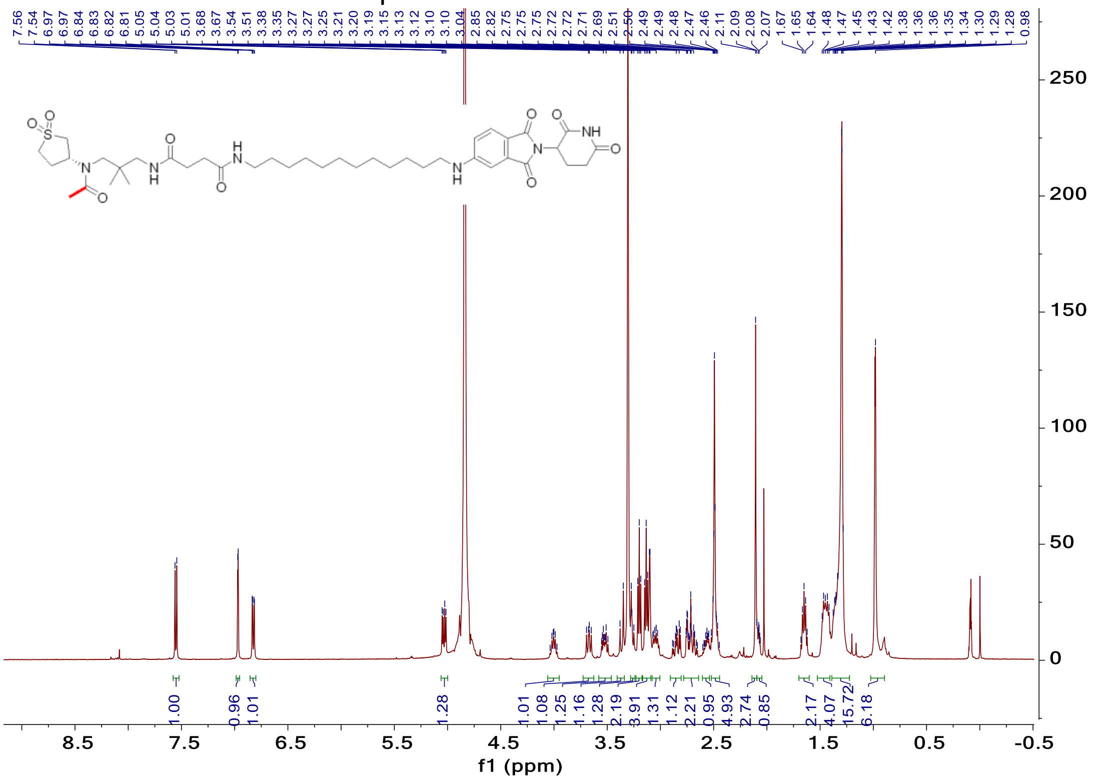

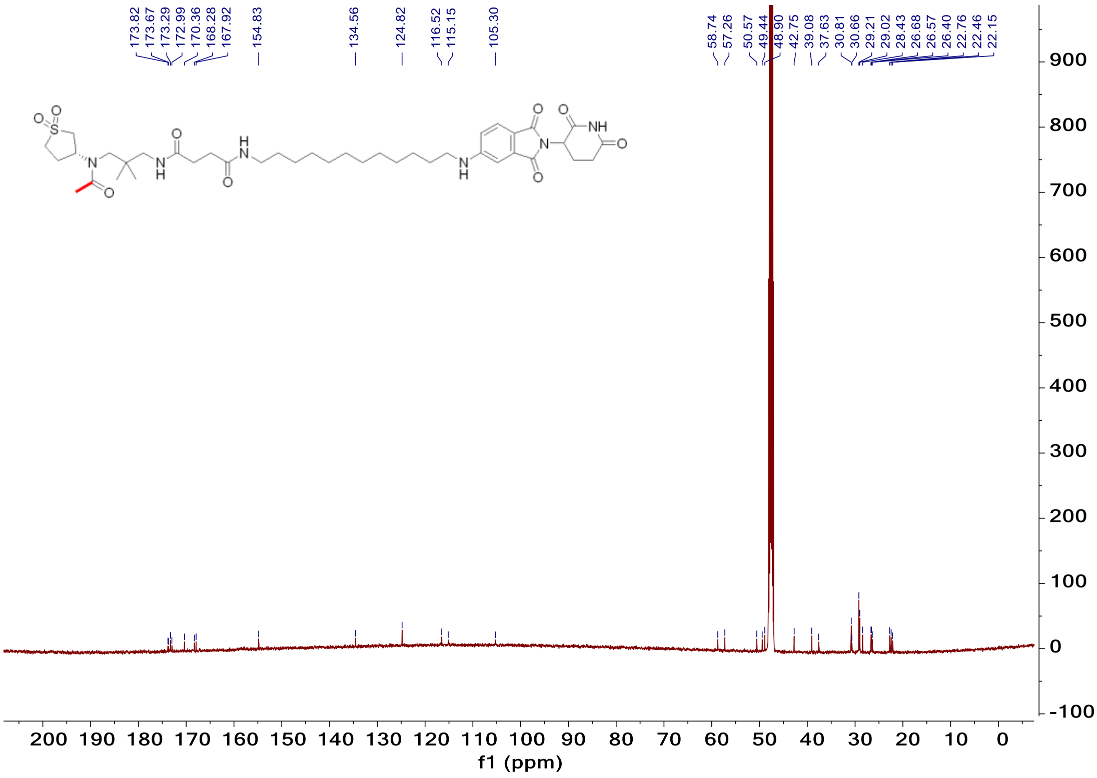

^1H NMR and ^{13}C NMR of compound **Sulfopin**

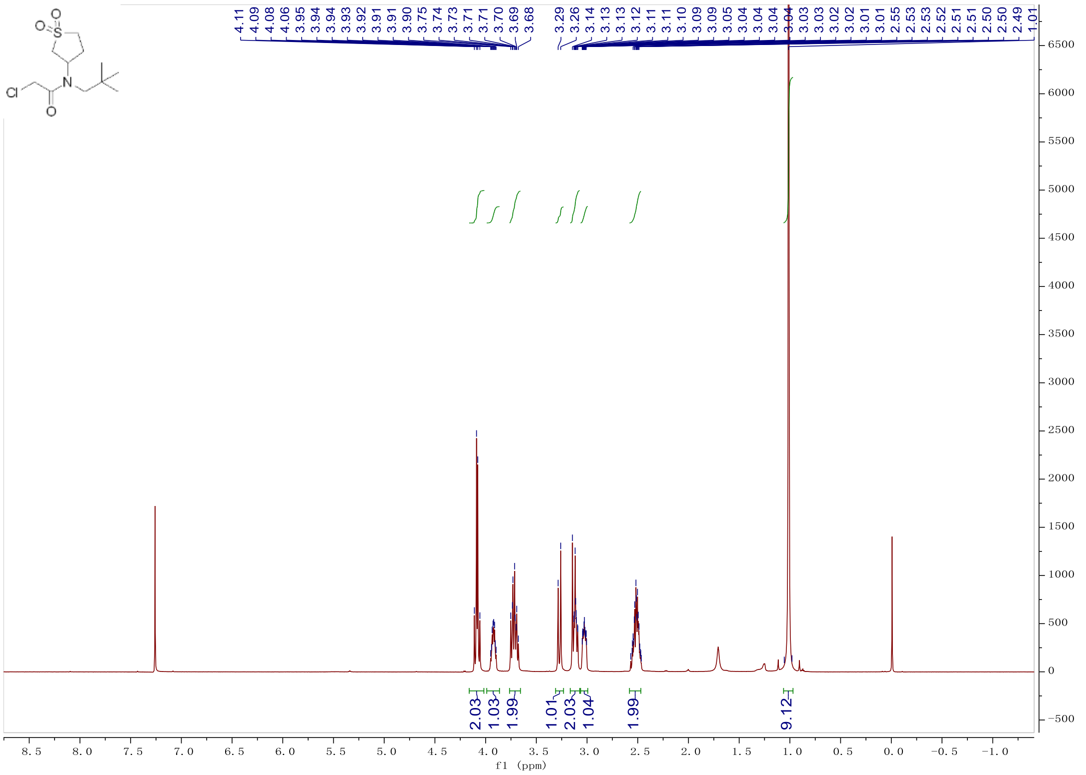

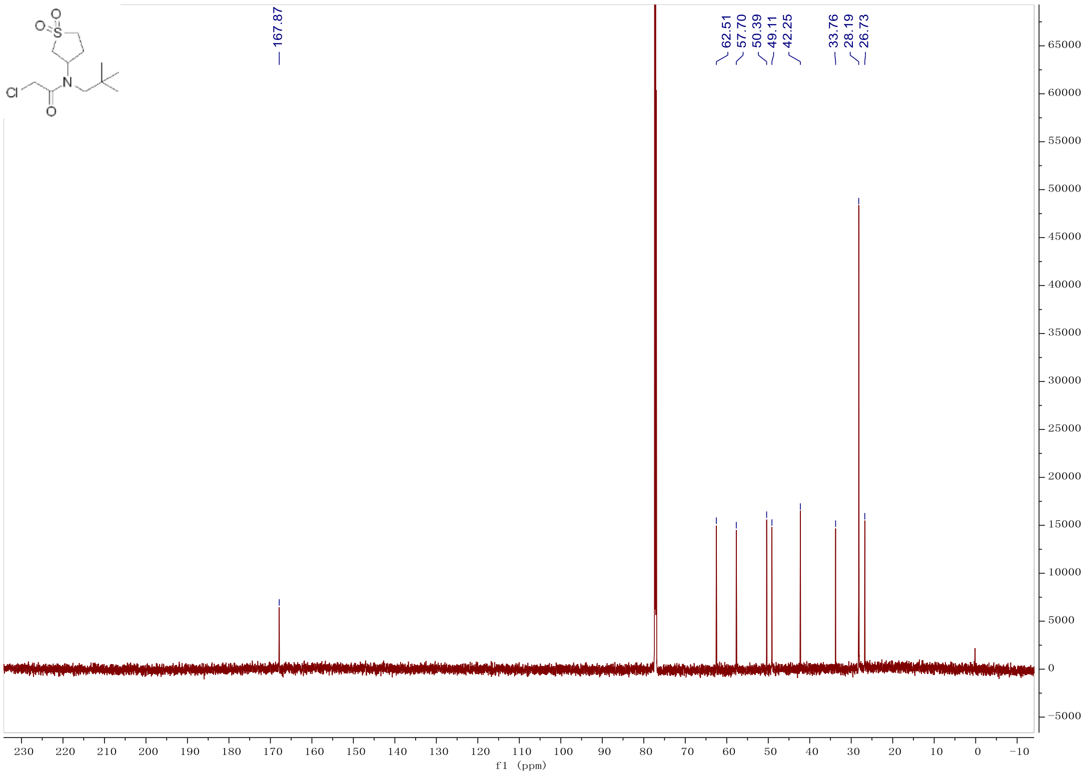

4. Experimental Section – Biological Assays

4.1. Cell lines and cell culture

MV-4-11 (IMDM + 10% FBS), HL-60 (IMDM + 20% FBS), THP-1 (1640 + 10% FBS), Kasumi-1 (1640 + 20% FBS), BDCM (1640 + 10% FBS), were from American Type Culture Collection (ATCC). MOLM-13 (1640 + 20% FBS), OCI-AML3 (1640 + 10% FBS) were kindly gifts from Changhai Hospital. CRBN knock-out MV-4-11 and ABT-199 resistant MV-4-11 (MV-4-11 R) were built on the basis of normal MV-4-11. 100 U/mL penicillin and 100 μg/mL streptomycin were added to the medium while cell culture. Cell cultures were maintained at 37 °C in an incubator containing 5% carbon dioxide.

4.2. Cell viability assay

Cell viability was measured with MTS assay. AML cells were seeded onto 96-well plate for 8000-20000 cells/well, compounds were added with various concentration. Cell plates were incubated at 37 °C with 5% CO_2 in incubator for 3 days. 20μL MTS reagent were added to each well of plated after incubation. At the end of the reaction, data was obtained by using a SpectraMax 190 microplate reader. The difference in absorbance values between 490 nm and 690 nm was used as the final data. Finally, the IC_{50} value of the compound was calculated by nonlinear regression method using GraphPad 8 software. Data results are from three independent replicate experiments.

4.3. Immunobloting

Cells were seeded in 12-well culture plates, 5 × $10^{\wedge 5}$ cells per well, and cultured with 1 mL culture medium containing the corresponding concentrations of the compounds. Protein lysates were made by 4 × Laemmli Sample Buffer (BIORAD, Richmond, USA). Appropriate amounts of protein samples were loaded and seperated by Tricine-SDS-PAGE, followed by Western blotting on Nitrocellulose membranes and probed with primary antibody. Antibodies used for the immunoblots: β-actin (am1021b, Abcepta), Pin1 (#3722S, CST), Mcl-1 (#94296, CST), CyclinD1 (#55506S, CST), Rb (#9309S, CST), phospho-Rb (#8516S, CST), NF-κB (#8242S, CST), Akt (#4691S, CST), Cleaved-caspase3 (#9664S, CST), γH2AX (#9718S, CST), Bip (#3177S, CST), eIF2α (#5324T, CST), phospho- eIF2α (#3398T, CST), ATF4 (#11815S, CST), PARP (#9532S, CST).

4.4. Apoptosis assay

Apoptosis was detected by Annexin V-FITC/PI Apoptosis Detection Kit (40302ES60, Yeasen), followed by flow cytometry analysis ((CytoFLEX flow cytometer, Beckman Coulter). The apoptosis rate of cells were counted in Annexin V+ gates. The bar graph was drawn by GraphPad 8 software.

4.5. Cell cycle assay

Cell cycle was detected by dying with PI and using flow cytometry to measure DNA contents. Image data was processed with ModFit LT5.0 and GraphpPad 8 software.

4.6. RT-qPCR

Reverse transcription followed by quantitative polymerase chain reaction (RT-qPCR). RNA was extracted using the TRIzol (Cat NO: 9109, Takara) according to manufacturer's manual. 625ng of total RNA was subjected to reverse transcription using cDNA Reverse Transcription kit according to the manufacturer's protocols (Takara). Then, real-time PCR using AceQR qPCR SYBRR Green Master Mix (Cat NO: Q131, vazyme) was performed on Stratagene MX3005P^{TM} (agilent). The relative gene expression was calculated by using the $2^{-\Delta\Delta CT}$ method.

4.7. Reactive oxygen species valuation

The ROS production was detected by Reactive Oxygen Species Assay Kit (50101ES01, YEASEN). Briefly, Intracellular reactive oxygen species can oxidize non-fluorescent DCFH to produce fluorescent DCF, and the amount of intracellular ROS can be reflected by flow cytometry. The image data was processed with FlowJo and GraphPad software.

4.8. RNA-seq and data analysis

MV-4-11 cells were seeded at 2 x 10^6 cells/2 mL in the 6-well plates and treated with 0.2% DMSO or 5 μM degrader for 20 and 36 hours. Cells were harvested and washed three times with 1 x PBS. Then the cells were centrifuged at 2000 rpm for 3 minutes to collect cell pellets in a refrigerated microfuge and frozen at -80 °C until further analysis. The cells were then lysed to extract RNA and sequenced using a second-generation sequencing method.

RNA-seq data were aligned to the hg19 genome reference. Count matrix was normalized by count2tpm function from the "IOBR" R package.[2] Differentially expressed genes (DEGs) were identified by "limma" R package.[3] Then, differentially expressed genes were defined as log2 fold change >1 or <−1 and FDR-adjusted p value <0.05. GSEA was carried out using the GSEA 4.2.1 software for testing enrichment of hallmark gene sets. Meanwhile, according to the

same dataset (hallmark), Gene set variation analysis (GSVA) was performed to demonstrate the signaling pathways alteration between the three groups using the "GSVA" R package.[4]

RNA-seq data discussd in this publication have been deposited in (Genome Sequence Archive for Human) GSA-human database and can be accessed in https://ngdc.cncb.ac.cn/gsa-human/browse/HRA004505.

4.9. Statistical analysis

The results were presented as means ± SEM. The significance analysis was conducted by two-tailed *t* test. Statistical analyses were performed using GraphPad Prism software 8.0. $P < 0.05$ was considered statistically significant (*$p< 0.05$, **$p <0.01$, ***$p < 0.001$, ****$p < 0.0001$).

5. Raw data

Figure 1

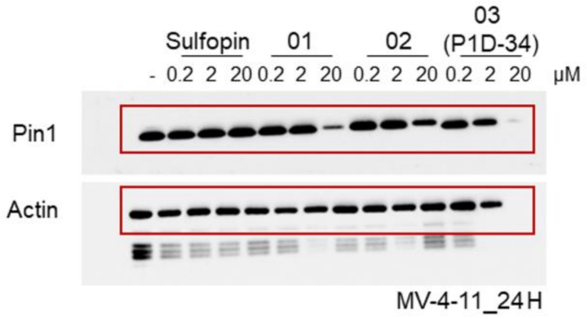

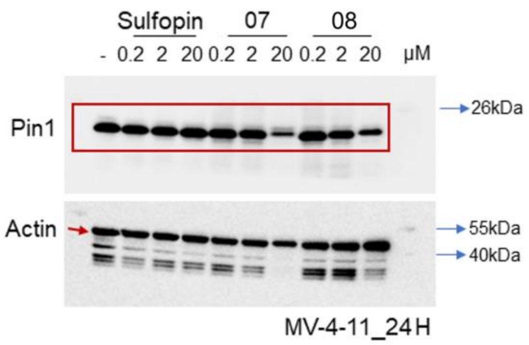

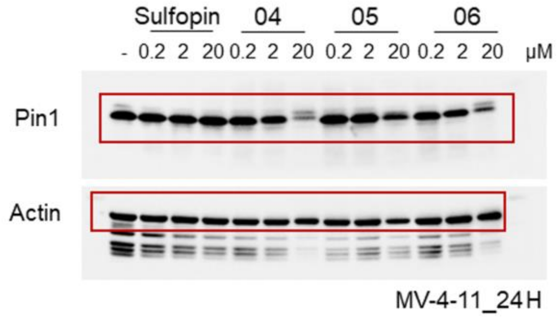

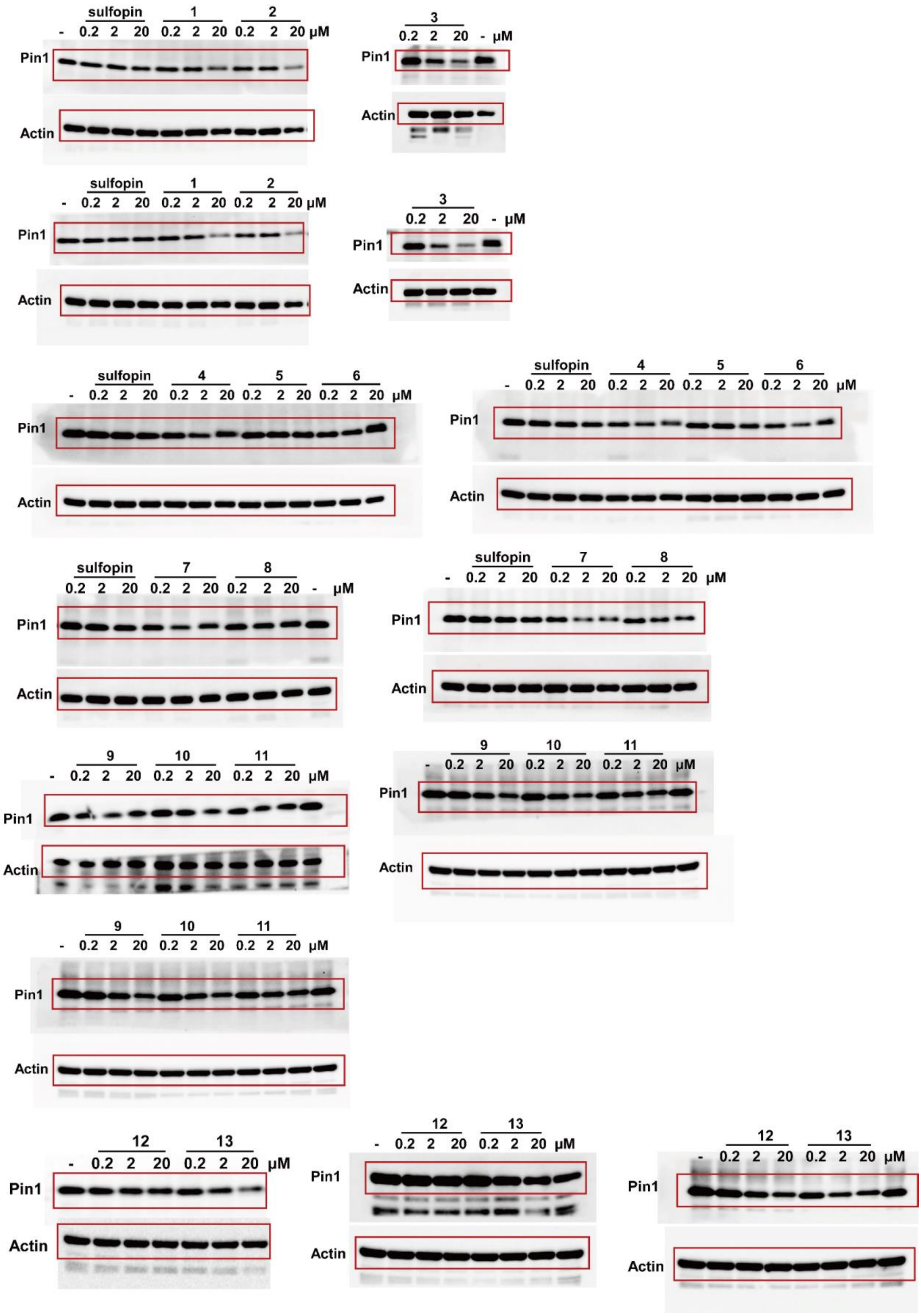
sulfopin 1 2
- 0.2 2 20 0.2 2 20 0.2 2 20 μM
Pin1
Actin
3
0.2 2 20 - μM
Pin1
Actin
sulfopin 1 2
- 0.2 2 20 0.2 2 20 0.2 2 20 μM
Pin1
Actin
3
0.2 2 20 - μM
Pin1
Actin
sulfopin 4 5 6
- 0.2 2 20 0.2 2 20 0.2 2 20 0.2 2 20 μM
Pin1
Actin
sulfopin 4 5 6
- 0.2 2 20 0.2 2 20 0.2 2 20 0.2 2 20 μM
Pin1
Actin
sulfopin 7 8
0.2 2 20 0.2 2 20 0.2 2 20 - μM
Pin1
Actin
sulfopin 7 8
- 0.2 2 20 0.2 2 20 0.2 2 20 μM
Pin1
Actin
9 10 11
- 0.2 2 20 0.2 2 20 0.2 2 20 μM
Pin1
Actin
9 10 11
- 0.2 2 20 0.2 2 20 0.2 2 20 μM
Pin1
Actin
9 10 11
- 0.2 2 20 0.2 2 20 0.2 2 20 μM
Pin1
Actin
12 13
- 0.2 2 20 0.2 2 20 μM
Pin1
Actin
12 13
- 0.2 2 20 0.2 2 20 μM
Pin1
Actin
12 13
- 0.2 2 20 0.2 2 20 μM
Pin1
Actin

Figure 2

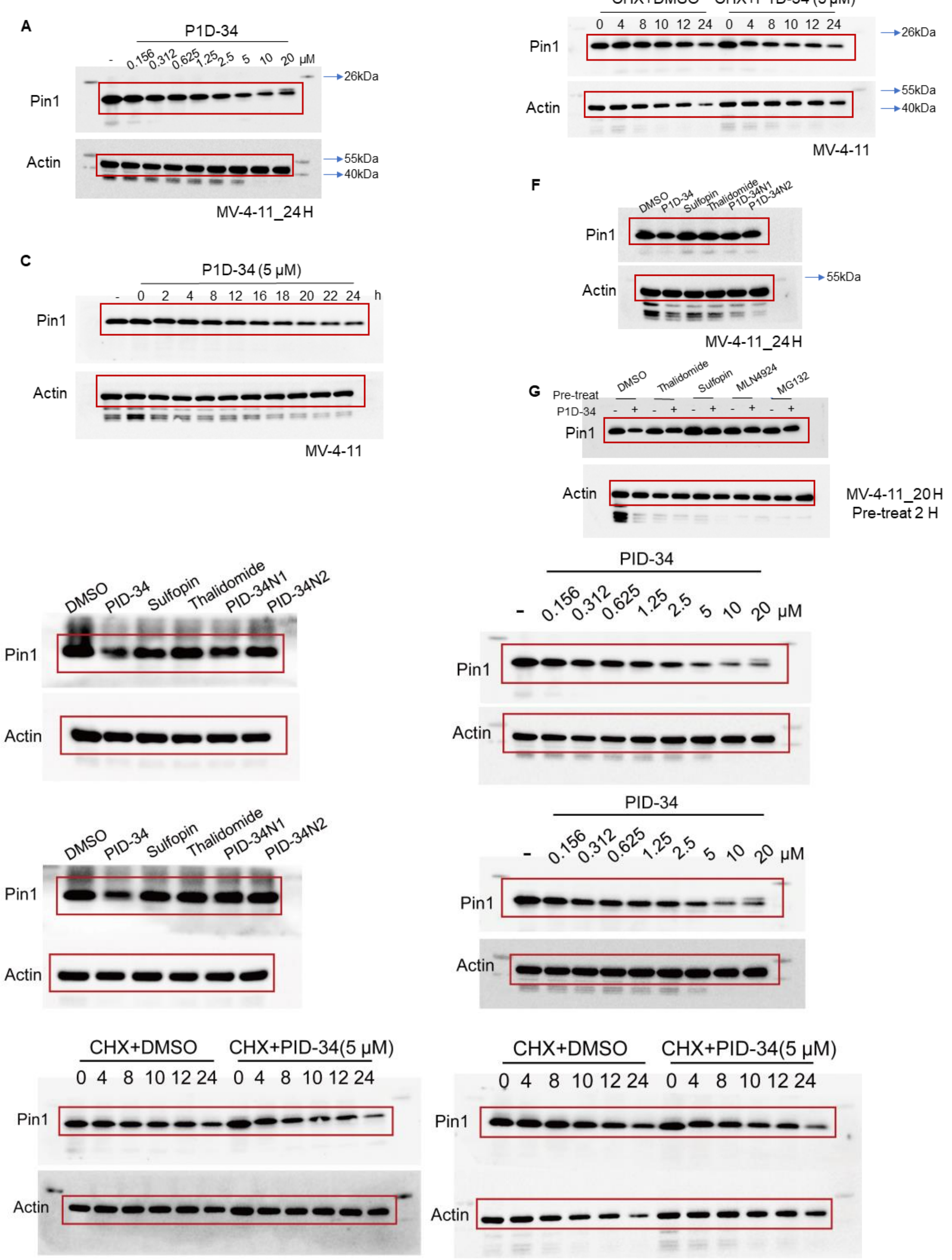
A
P1D-34
- 0.156 0.312 0.625 1.25 2.5 5 10 20 μM
Pin1
Actin
26kDa
55kDa
40kDa
MV-4-11_24H
C
P1D-34 (5 μM)
- 0 2 4 8 12 16 18 20 22 24 h
Pin1
Actin
MV-4-11
D
CHX+DMSO
CHX+P1D-34 (5 μM)
0 4 8 10 12 24 0 4 8 10 12 24
Pin1
Actin
26kDa
55kDa
40kDa
MV-4-11
F
DMSO P1D-34 Sulfopin Thalidomide P1D-34N1 P1D-34N2
Pin1
Actin
55kDa
MV-4-11_24H
G
Pre-treat
P1D-34
DMSO Thalidomide Sulfopin MLN4924 MG132
- + - + - + - + - +
Pin1
Actin
MV-4-11_20H
Pre-treat 2 H
DMSO PID-34 Sulfopin Thalidomide PID-34N1 PID-34N2
Pin1
Actin
PID-34
- 0.156 0.312 0.625 1.25 2.5 5 10 20 μM
Pin1
Actin
DMSO PID-34 Sulfopin Thalidomide PID-34N1 PID-34N2
Pin1
Actin
PID-34
- 0.156 0.312 0.625 1.25 2.5 5 10 20 μM
Pin1
Actin
CHX+DMSO
CHX+PID-34(5 μM)
0 4 8 10 12 24 0 4 8 10 12 24
Pin1
Actin
CHX+DMSO
CHX+PID-34(5 μM)
0 4 8 10 12 24 0 4 8 10 12 24
Pin1
Actin

Figure 3

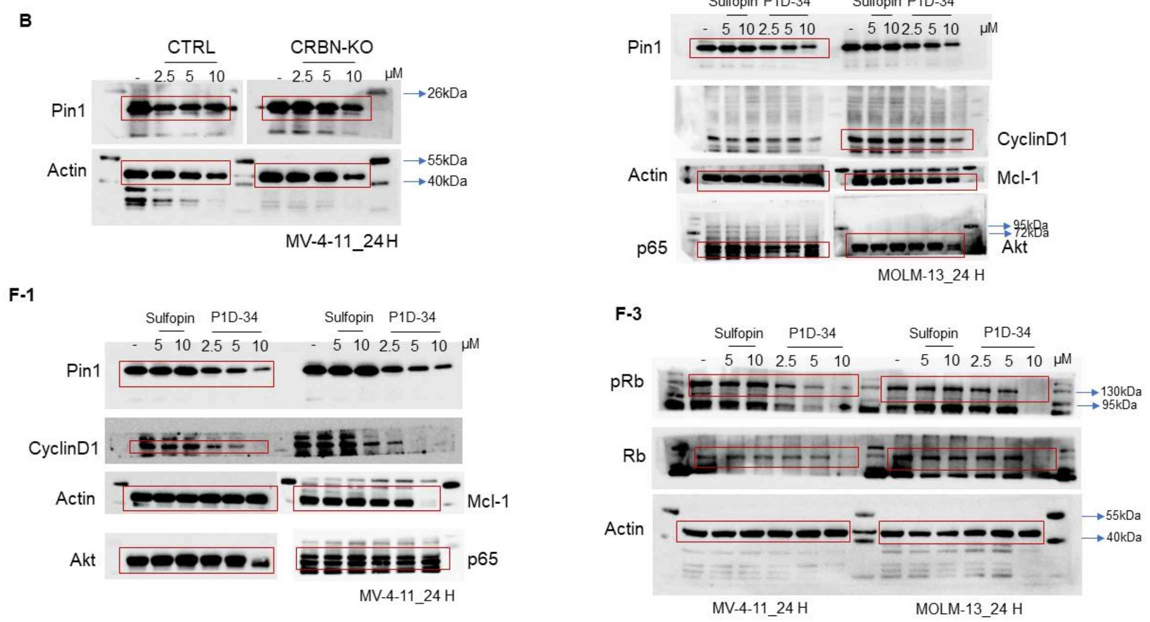

Figure 4

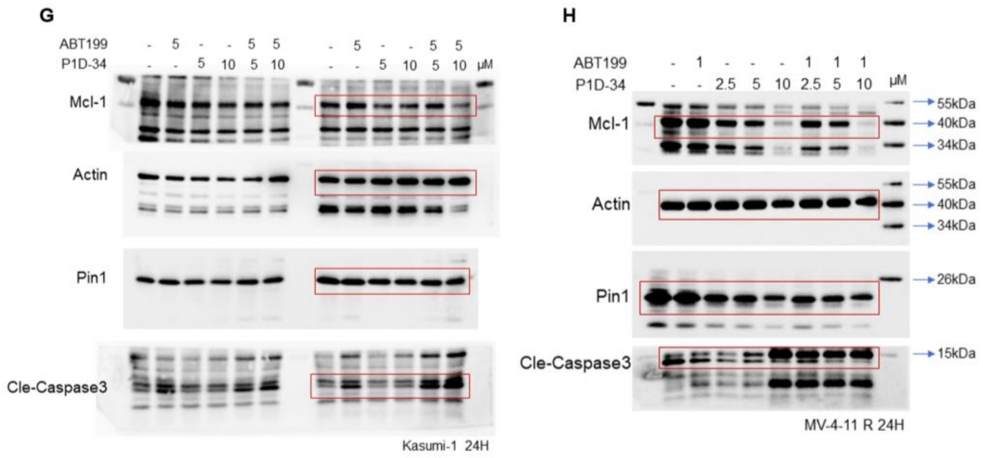

Figure 5

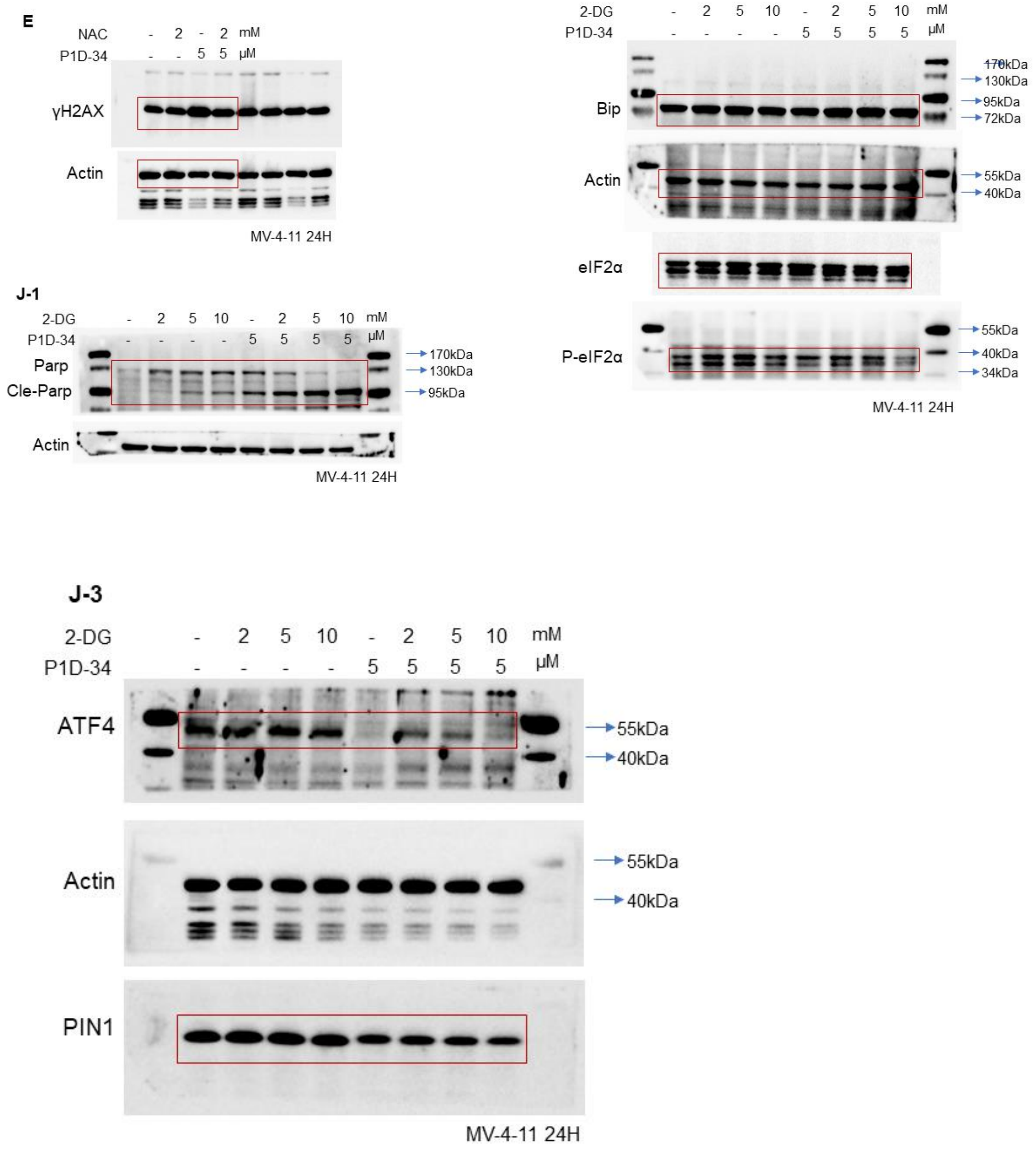

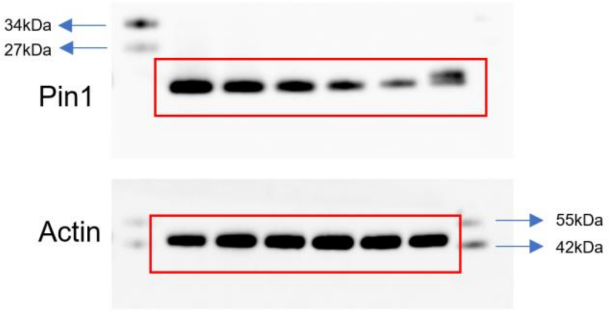

(Supplementary Fig. S3B)

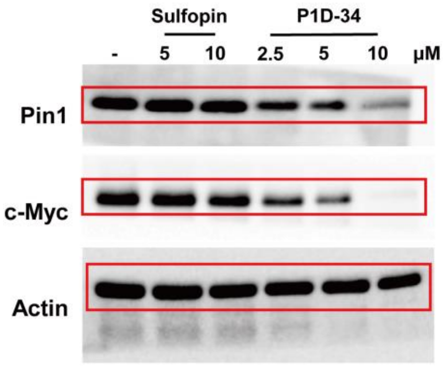

(Supplementary Fig. S7)

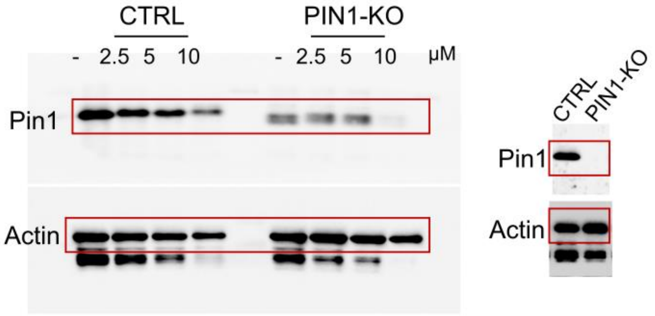

(Supplementary Fig. S5C-D)

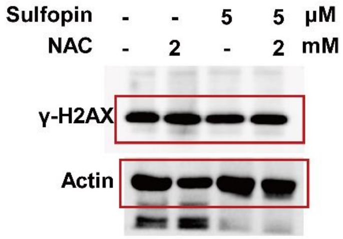

(Supplementary Fig. S9F)

References

(1) C. Dubiella; B. J. Pinch; K. Koikawa; D. Zaidman; E. Poon; T. D. Manz; B. Nabet; S. He; E. Resnick; A. Rogel; E. M. Langer; C. J. Daniel; H. S. Seo; Y. Chen; G. Adelmant; S. Sharifzadeh; S. B. Ficarro; Y. Jamin; B. Martins da Costa; M. W. Zimmerman; X. Lian; S. Kibe; S. Kozono; Z. M. Doctor; C. M. Browne; A. Yang; L. Stoler-Barak; R. B. Shah; N. E. Vangos; E. A. Geffken; R. Oren; E. Koide; S. Sidi; Z. Shulman; C. Wang; J. A. Marto; S. Dhe-Paganon; T. Look; X. Z. Zhou; K. P. Lu; R. C. Sears; L. Chesler; N. S. Gray; N. London. *Nat Chem Biol* 2021, ***17*** (9), 954-963.

(2) Zeng, D.; Ye, Z.; Shen, R.; Yu, G.; Wu, J.; Xiong, Y.; Zhou, R.; Qiu, W.; Huang, N.; Sun, L.; Li, X.; Bin, J.; Liao, Y.; Shi, M.; Liao, W. *Front Immunol* 2021, ***12***, 687975.

(3) Ritchie, M. E.; Phipson, B.; Wu, D.; Hu, Y.; Law, C. W.; Shi, W.; Smyth, G. K. *Nucleic Acids Res* 2015, ***43*** (7), e47.

(4) Hanzelmann, S.; Castelo, R.; Guinney, J. *BMC Bioinformatics* 2013, ***14***, 7.
